# The Role of CAF‐derived Vitronectin in Promoting Colorectal Cancer Progression and Immunosuppression

**DOI:** 10.1002/advs.202505769

**Published:** 2025-06-20

**Authors:** Jiahua Yu, Mengxi Xiu, Shijun Yu, Zhiqin Chen, Yandong Li, Yong Gao

**Affiliations:** ^1^ Department of Oncology Shanghai East Hospital School of Medicine Tongji University Shanghai 200120 China; ^2^ Department of Radiation Oncology Shanghai Chest Hospital Shanghai Jiao Tong University School of Medicine Shanghai 200030 China

**Keywords:** cancer‐associated fibroblasts, colorectal cancer, macrophages, SLC6A8, vitronectin

## Abstract

Cancer‐associated fibroblasts (CAFs) dominate the tumor stroma in colorectal cancer (CRC), fostering an immunosuppressive microenvironment that supports tumor growth, metastasis, and therapy resistance. Targeting CAF‐derived cytokines and secreted proteins offers potential new avenues for CRC treatment. Here, vitronectin (VTN) is identified as a significantly up‐regulated gene in CAFs, correlated with poor prognosis in CRC patients, through multi‐dataset analysis and multiplex immunofluorescence. Comprehensive analyses, including cytometry by time ‐of‐ flight (CyTOF), in vitro co‐culture assays, transgenic mouse models, and azoxymethane (AOM)/dextran sodium sulfate (DSS)‐induced CRC models, demonstrate that VTN enhances CRC proliferation, metastasis, and resistance to therapy. CyTOF analysis shows a reduced proportion of M2‐type macrophages in fibroblast‐specific VTN knockout mice (*Vtn^fl/fl^ S100a4‐Cre^+^*), with VTN promoting M2 macrophage polarization in vitro. Mechanistically, VTN upregulates SLC6A8 expression in CRC cells and macrophages by enhancing FAK phosphorylation, which increases creatine and ATP uptake, promoting cancer progression and macrophage polarization. Targeted VTN knockout in fibroblasts significantly improves the efficacy of immunotherapies and reduces CRC progression. These findings highlight the dual role of CAF‐derived VTN in driving CRC malignancy and modulating immune responses, positioning VTN as a promising therapeutic target in CRC management.

## Graphical abstract

Cancer‐associated fibroblast (CAF)‐derived VTN upregulates SLC6A8 expression in colorectal cancer (CRC) cells and macrophages through enhanced FAK phosphorylation. This modulation significantly increases the cellular uptake of creatine and ATP, promoting cancer progression and macrophage polarization. Targeted knockout of VTN in fibroblasts augments immunotherapy efficacy and markedly suppresses the progression of CRC, which offers potential therapeutic strategies.

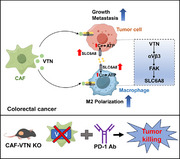

## 1 Introduction

Colorectal cancer (CRC) represents a significant global health challenge, imposing a substantial burden in terms of both morbidity and mortality. It ranks as the third most frequently diagnosed malignancy worldwide and stands as the second leading cause of cancer‐related deaths.^[^ ^1^, ^2^ ^]^ Patients diagnosed with metastatic CRC face grim prognoses, with a median overall survival (OS) of merely 25–30 months.^[^ ^3^, ^4^ ^]^ Compounded by the limited efficacy of chemotherapy and targeted therapies, the development of effective treatment strategies for CRC remains a formidable challenge.

Immunotherapy has emerged as a promising avenue in the treatment of CRC, particularly benefiting patients with metastatic CRC characterized by microsatellite instability (MSI).^[^ ^5^, ^6^ ^]^ The tumor microenvironment (TME) significantly influences the response to immunotherapy in CRC.^[^ ^7^, ^8^ ^]^ Among its constituents, cancer‐associated fibroblasts (CAFs) predominate in the CRC stroma, creating a suppressive milieu conducive to tumor growth and immune evasion.^[^ ^9^, ^10^ ^]^ In various cancers, including CRC, patients exhibiting a high mesenchymal tumor component generally experience poorer prognoses compared to those with lower mesenchymal levels.^[^ ^11^, ^12^, ^13^, ^14^ ^]^ CAFs exert their influence by modulating tumor and stromal cell biology through the release of various proteomes, including cytokines, chemokines, and extracellular matrix proteins, and thus affect the progression and therapeutic resistance of CRC.^[^ ^15^, ^16^, ^17^, ^18^, ^19^ ^]^ For instance, CAF‐derived WNT2 plays a crucial role in the oncogenic progression of CRC, and monoclonal antibodies targeting WNT2 have been shown to significantly enhance the efficacy of immune checkpoint inhibitors (ICIs).^[^ ^15^ ^]^ Additionally, netrin‐1, which is upregulated in CAFs, facilitates neoplastic interactions between CAFs and CRC cells. Inhibiting netrin‐1 could thus reduce this pro‐neoplastic cross‐talk, effectively curtailing cancer plasticity.^[^ ^19^ ^]^ Targeting these secreted proteins holds promise for advancing early diagnosis and treatment strategies for CRC, offering innovative avenues for comprehensive therapeutic interventions.

Through the use of public databases and bioinformatic analysis, we have identified vitronectin (VTN) as the most highly up‐regulated gene specifically expressed in CAFs compared to normal fibroblasts (NFs). VTN, a multifunctional glycoprotein commonly found in the extracellular matrix and plasma, plays pivotal roles in regulating cell adhesion, migration, proliferation, and differentiation.^[^ ^20^, ^21^ ^]^ Instead of directly contacting cells, VTN typically interacts with cells by transmitting biphasic signals through specific binding to integrins in the cell membrane.^[^ ^22^, ^23^ ^]^ Secreted forms of VTN in human plasma have been identified as potential biomarkers for the diagnosis and prognosis of various cancers, including CRC.^[^ ^24^, ^25^, ^26^ ^]^ It has been implicated in promoting the proliferation and metastasis of certain tumors, such as hepatocellular carcinoma, ovarian cancer, and breast cancer.^[^ ^27^, ^28^, ^29^ ^]^ However, the mechanisms by which CAF‐secreted VTN functionally contributes to CRC progression remain unclear.

In this study, primary human CAFs and NFs were isolated and subjected to a series of in vivo and in vitro experiments to elucidate the pro‐cancer effects of VTN in CRC. Cytometry by Time‐of‐Flight (CyTOF) analysis was utilized to detect azoxymethane (AOM)/dextran sodium sulfate (DSS)‐induced CRC in transgenic mice, revealing the impact of VTN on tumor‐associated macrophages (TAMs) within the immune microenvironment. Furthermore, combination therapy targeting VTN with immunotherapy demonstrated significant efficacy in inhibiting CRC growth, highlighting VTN as a promising therapeutic target for CRC treatment.

## 2 Materials and Methods

### 2.1 Cell Lines

Cell lines were all obtained from Shanghai Stem Cell Bank, Chinese Academy of Sciences (Shanghai, China). RKO, DLD1, Caco2, MC38, and RAW264.7 cells were maintained in DMEM (Sigma, USA) containing 10% fetal bovine serum (FBS) supplemented with antibiotics at 37 °C in a humidified atmosphere with 5% CO_2_. SW620 cells were cultured in Leibovitz's L‐15 medium (Gibco, USA). Lovo cells were maintained in Ham's F‐12K medium (Gibco, USA). HCT116 and HT29 cells were cultured in McCoy's 5A medium (Gibco, USA). THP‐1, NIH3T3, and CT26 cells were cultured in RPMI 1640 medium (Corning, USA), under the same growth conditions.

### 2.2 Patients and Samples

Primary CRC tissues and their adjacent normal tissues were collected immediately after surgical resection from Shanghai East Hospital (Shanghai, China) to isolate primary NFs, CAFs, and patient‐derived tumor organoids. Blood samples were collected from 36 colorectal cancer patients who had not yet received any treatment. For comparative analysis, plasma samples from 35 healthy individuals were also obtained. A CRC tissue microarray (#LD‐COC1602) was obtained from Outdo Biotech Co., Ltd. (Shanghai, China), which consists of 80 pairs of tumors and adjacent normal tissues. All clinical samples used in this study were approved by the Medical Ethics Committees of Shanghai East Hospital. Informed consent was obtained from all participants involved in the study.

### 2.3 Isolation and Culture of Primary NFs and CAFs

A total of 34 primary CAFs were isolated from fresh human CRC tissue, while NFs were isolated from non‐cancerous regions at least 5 cm from the tumor margin in the same patient undergoing surgical resection. The tissues were minced and dissociated in DMEM with collagenase Type I (400 U mL^−1^, Thermo Fisher, USA) for 1 h at 37 °C. The cell precipitates were washed twice with PBS and then incubated with DMEM/F12 in 10% FBS containing antibiotics (100 U mL^−1^) and amphotericin B (2 µg mL^−1^) in a 37 °C, 5% CO_2_ incubator.

### 2.4 Cell Proliferation Assay

Cell viability was evaluated using a Cell Counting Kit‐8 (CCK‐8) kit (Dojindo, Japan). 3000 cells were seeded per well in a 96‐well plate with 100 µL DMEM and incubated overnight. CCK‐8 reagent was added to each well and incubated for 75 min. Optical density (OD) values at 450 nm were measured. The colony formation assay was performed by incubating 2000 cells per well in a 6‐well plate for approximately2 to 3 weeks. Colonies were then stained with Crystal Violet and photographed.

### 2.5 Cell Migration Assays

Cell migration was detected using Transwell chamber assays (Millipore, USA). The upper chamber was seeded with cells in 400 µL of serum‐free medium, while the lower chamber was supplemented with 800 µL DMEM and 10% FBS. After incubation for 24–48 h, non‐migrated cells were removed from the upper chamber using a cotton swab. Migrated cells adhering to the bottom surface of the membrane were subsequently stained with crystal violet and enumerated using microscopy.

### 2.6 Western Blot Analysis

Total protein was extracted from the cell lysates using RIPA buffer (Millipore, USA) supplemented with protease inhibitors. Equal amounts of protein were separated by SDS‐PAGE and transferred onto a NC membrane (Bio‐Rad, USA). After blocking with 5% non‐fat milk, the membrane was probed with primary antibodies overnight at 4 °C, followed by incubation with corresponding secondary antibodies. Protein bands were visualized using the Odyssey Digital Infrared Imaging System (LiCoR Biosciences, USA) or enhanced chemiluminescence (Bio‐Rad, USA). All antibodies used for western blot are listed in Table S1 (Supporting Information).

### 2.7 qRT‐PCR

Total RNA was isolated from cells using TRI Reagent (Sigma‐Aldrich, USA) according to the manufacturer's manual. For quantitative real‐time PCR (qRT‐PCR), cDNA was synthesized from the extracted RNA using a reverse transcription kit (Takara Bio, Japan), and gene expression levels were analyzed using specific primers and the SYBR Green master mix (Takara Bio, Japan). All sequences of primers used in this study for qRT‐PCR are listed in Table S2 (Supporting Information).

### 2.8 RNA‐Sequencing

Total RNA was extracted from DLD1‐LV‐VEC and DLD1‐LV‐VTN cells following the manufacturer's protocol. RNA integrity was assessed using the Agilent 2100 Bioanalyzer (Agilent Technologies, USA). RNA sequencing was conducted by Biomarker Technologies Co. Ltd (Beijing, China) using the Hieff NGS Ultima Dual‐Mode mRNA Library Prep Kit for Illumina according to the manufacturer. The raw reads were further processed with a bioinformatic pipeline tool, BMKCloud(www.biocloud.net) online platform. Differential expression analysis was conducted using DESeq2 (Love et al., 2014). Genes with a p‐value < 0.05 and an absolute log2 fold change (|log2FC|) greater than 1.5 were considered differentially expressed. The top 14 genes with the highest absolute log2FC values were selected for further analysis, while undefined novel genes were excluded from the final list to ensure reliability and relevance of the results.

### 2.9 Apoptosis Analysis

The impact of different cell supernatants or recombinant human vitronectin (rhVTN, MedChemExpress, USA) on oxaliplatin‐induced apoptosis in RKO and DLD1 cells was assessed using the annexin V‐FITC/propidium iodide (PI) apoptosis detection kit (Dojindo, Japan) followed by flow cytometry. RKO and DLD1 cells were cultured in conditioned media from CAF‐shNC or CAF‐shVTN, or supplemented with rhVTN. Subsequently, cells were treated with oxaliplatin/5‐ Fluorouracil(5‐FU)/SN‐38 for 24 or 48 h. Following treatment, cells were harvested, centrifuged, and incubated with 5 µL of Annexin V and 5 µL of PI for 15 min at room temperature. Data analysis was performed using FlowJo software (FlowJo, LLC).

### 2.10 Immunofluorescent Staining

Cells grown on coverslips or tissue sections were fixed with paraformaldehyde, permeabilized with 0.1% NP40, and blocked with serum. Samples were then incubated with primary antibodies overnight at 4 °C, followed by incubation with fluorescently labeled secondary antibodies. Primary antibodies were listed in Table S1 (Supporting Information). Nuclei were counterstained with DAPI. Images were acquired using a fluorescence microscope and analyzed using image analysis software.

### 2.11 Flow Cytometry

Single‐cell suspensions were prepared from cultured cells and stained with fluorescent‐conjugated antibodies against F4/80, CD11b, and CD206 (Biolegend, USA) for 30 min at 4 °C. Samples were then analyzed using a Beckman CytoFLEX flow cytometer equipped with appropriate lasers and detectors.

C57BL/6J mice bearing subcutaneous tumors were euthanized, and tumors were excised and minced into small pieces. The tissues were incubated in DMEM medium (Gibco, USA), 1% penicillin‐streptomycin, 50 mg mL^−1^ collagenase I (Solarbio, China), and 20 mg/mL DNase I (Solarbio, China) at 37 °C for 30 min. After digestion, the suspension was filtered through a 70 µm cell strainer, and the cell pellet was washed with RBC lysis buffer (eBioscience, USA) to remove red blood cells. The cells were resuspended in PBS.For intracellular staining, cells were permeabilized with cell permeabilization buffer (eBioscience, USA) for 30 min, washed, and stained with Fixable Viability Stain 510 (BD Pharmingen, USA) for 15 min. After blocking with anti‐CD16/32 (BioLegend, USA), the cells were incubated with anti‐CD11b, anti‐CD45, anti‐F4/80 (BD Pharmingen, USA), and anti‐CD206 (BioLegend, USA) antibodies for 30 min on ice. After washing, cells were resuspended in PBS for flow cytometry. Data analysis was performed using FlowJo v10 to determine cell populations and protein expression levels.

### 2.12 Cytometry by Time‐of‐Flight (CyTOF) Analysis

Mouse tumor samples were subjected to CyTOF analysis and then dissociated according to the manufacturer's instructions. Cells were then collected and stained with 42 antibodies using the Helios3 CyTOF system by PLTTech (Hangzhou, China). Data were processed using the Cytobank platform to identify cell populations and quantify protein expression levels at the single‐cell level. Detailed antibody information is listed in Table S3 (Supporting Information).

### 2.13 Animal Models

The mouse experiments were conducted following the Guidelines on Care and Use of Laboratory Animals and approved by the Animal Experimentation Ethics Committee of Shanghai East Hospital, Tongji University. Female BALB/c‐nu/nu mice and BALB/c mice aged 4–6 weeks were purchased from GerPharmatech (Nanjing, China). *Vtn* ^flox/+^ mice (T019096) and *S100a4*‐CreERT2 mice (T006589) were also purchased from GerPharmatech (Nanjing, China). The two mouse lines were kept on the C57BL/6 J background and were crossed in‐house to create *Vtn^fl/fl^ S100a4‐Cre^+^* mice.After being injected with tamoxifen (75 mg kg^−1^, intraperitoneally) for five consecutive days, the transgenic mice described above were used for subsequent experiments.

To examine the impact of CAF‐secreted VTN on the growth and metastasis of CRC, we injected a single‐cell suspension containing either CT26 (0.5 × 10^6^ cells) alone or CT26 (0.25 × 10^6^ cells) with NIH3T3‐LV‐VEC or NIH3T3‐LV‐VTN (1 × 10^6^ cells) into the skin of the right flank of BALB/c‐nu/nu (n = 18) and BALB/c mice (n = 15). Furthermore, we constructed an AOM/DSS chemically induced intestinal cancer model using VTN CKO mice. The mice were injected intraperitoneally with a single dose of AOM (10 mg kg^−1^, Sigma, USA) and then given drinking water containing 2% DSS (MP Biomedicals, USA) for 7 days, followed by normal drinking water for 14 days, for a total of 3 cycles. The mice were weighed or observed 2–3 times per week. During these observations, any instances of rectal bleeding, diarrhea, or prolapse were noted. For testing the effect of VTN on tumor metastasis, we injected the mixed cells mentioned above into the spleens of BALB/c mice (n = 10) to induce liver metastases.

To investigate the effect of VTN on macrophage M2 polarization, MC38 cells (0.5 × 10^6^) were subcutaneously implanted into the right flank of *Vtn^fl/fl^ S100a4‐Cre^+^* and *Vtn^fl/fl^ S100a4‐Cre^‐^* mice. This setup allowed for subsequent flow cytometry analysis to determine the proportion of M2 macrophages in the different groups. Additionally, NIH3T3‐LV‐VEC and NIH3T3‐LV‐VTN cells (1 × 10^6^ cells) were mixed with CT26 cells (0.5 × 10^6^ cells) at a 2:1 ratio and subcutaneously injected into BALB/c mice (n = 20). On day 1 post‐tumor inoculation, mice in the experimental group were administered an intraperitoneal injection of clodronate liposomes (200 µL per mouse, twice per week), while control mice received the same volume of PBS liposome.

To assess the impact of RGX202 therapy on the tumor‐promoting effects of VTN, we injected NIH3T3‐LV‐VEC and NIH3T3‐LV‐VTN cells (1 × 10^6^ cells) mixed with CT26 cells (0.5 × 10^6^ cells) in a 2:1 ratio subcutaneously into BALB/c mice (n = 20). On the fifth day of subcutaneous tumor formation, each group was randomly divided into two groups. One group was administered RGX202 (Selleck, USA) at a dose of 500 mg/kg once a day by gavage, while the other group served as the control group.

To investigate the effect of VTN on CRC treatment with aPD1, we used the subcutaneous tumor formation model in mice co‐injected with CT26 and NIH3T3 (LV‐VEC and LV‐VTN) cells, as previously described. On day 5 after inoculation with mixed cells, each group was further randomized into two groups. One group received IgG control treatment, and the other group received aPD1 treatment (200 µg/each, intraperitoneal injection, every 3 days). At the same time, MC38 cells (0.5 × 10^6^) were subcutaneously implanted in the right flank of the *Vtn^fl/fl^ S100a4‐Cre^+^* and *Vtn^fl/fl^ S100a4‐Cre^‐^* mice. Five days after injection, we performed grouping and aPD1 treatment as previously described. In addition, we tested the therapeutic effect of PD1 on the *Vtn^fl/fl^ S100a4‐Cre^+^* and *Vtn^fl/fl^ S100a4‐Cre^‐^* mouse models of AOM/DSS‐induced CRC. After completing three rounds of DSS induction, we performed grouping and aPD1 treatment as described above. Mouse aPD1 was obtained from Shanghai Junshi Biosciences Co., Ltd.

At the conclusion of the experiment, the mice from the different models mentioned above were humanely euthanized. Subcutaneous tumors or liver tissues were removed and weighed. For AOM/DSS‐induced CRC mice, the abdominal cavity was opened along the mid‐abdominal line and the intestinal tissues were isolated from the anus to the ileum, the intestines were dissected vertically and then rinsed with PBS, and the intestinal tumor formation was observed and recorded. Following this, the tumor or liver tissue samples were fixed and embedded in paraffin.

### 2.14 Patient‐Derived Tumor Organoid (PDTO) Isolation and Culture

PDTO was isolated from freshly dissected CRC tissue. The tissue was washed, minced, and added to the tumor tissue digestion solution (bioGenous, CN) for 1 h at 37 °C. After digestion into clumps of cells, the sample was filtered through a 100 µm mesh and seeded into Organoid Culture ECM (bioGenous, CN) in 24‐well plates. Once Matrigel polymerization was complete (20 min at 37 °C), 500 µL of Colorectal Cancer Organoid complete medium (bioGenous, CN) was added. To prevent anoikis, 10 µM Y27632 (Selleck, USA) was added for 48 h. The organoids were then infected with either a control lentivirus or VTN overexpression lentivirus using polybrene (10 µg/mL, Millipore, USA). The medium was replaced every 2–3 days.

### 2.15 Immunohistochemistry Staining

Immunohistochemical staining (IHC) was performed on the CRC tissue microarray (#LD‐COC1602) and tumor tissues by standard procedures, and the IHC score for each slide was assessed in a double‐blind manner by two pathologists. The staining intensity was scored as 0 to 3: 0 (negative staining); 1 (weak staining); 2 (moderate staining); and 3 (strong staining). The positive tumor cell proportion was scored 0 to 4: 0 (<10%), 1 (10‐25%), 2 (25‐50%), 3 (50‐75%), and 4 (>75%). An IHC score (“staining intensity” ´or “positive tumor cell percentage”) ≤6 was defined as weak staining and >6 as strong staining. Primary antibodies were listed in Table S1 (Supporting Information).

### 2.16 ELISA

Blood samples and cell culture medium were centrifuged (3000 g for 30 min at 4 °C) within 6 h of collection, and supernatants were collected and stored at 80 °C. The dilution for the plasma sample was 10000‐fold. The concentration of VTN was detected with a Vitronectin Human ELISA Kit (Thermo Fisher, USA) according to the manufacturer's instructions. All sample tests were run in triplicate. The absorbance was measured on an ELISA plate reader set at 450 nm and 550 nm. A standard curve was run with each assay. The final absorbance values were obtained from the average of 3 duplicates (450–550 nm) within 10% of the mean value.

### 2.17 Application of Databases to Screen Genes Highly Expressed in CAF of CRC

The study obtained data on NF and CAF in colorectal cancer patients by searching the GEO database, which includes the gene microarray dataset GSE51257, the RNA‐seq database GSE92945, and the scRNA‐seq database GSE231559. The differentially expressed genes in the NF and CAF groups were analyzed using the online tool GEO2R, with filtering conditions of P<0.15 and |log2FC|≥2. Simultaneously, we downloaded the expression matrix of 6 tumor tissues and 3 adjacent normal tissues for the single‐cell transcriptome sequencing data of CRC from GSE231559. We identified specific cellular subpopulations using the “FindMarkers” function in the Seurat package and Single R. The marker genes for each cell subpopulation were then grouped and annotated. Fibroblasts were extracted and differentially expressed genes (MAST) were analyzed between NF and CAF using the FindMarkers and FindAllMarkers functions. The results of the differential gene analysis from the three datasets were integrated to filter out genes that were highly expressed in CAF of CRC.

### 2.18 Bioinformatics Analysis

Timer2.0 (https://cistrome.shinyapps.io/timer/) was used to analyze the correlation between VTN and different levels of cellular infiltration in CRC. The correlation between VTN expression levels and prognosis and clinical factors in CRC patients was analyzed using BEST (https://rookieutopia.com/app_direct/BEST/#PageHomeAnalysisModuleSelection).

### 2.19 Co‐Culture Experiments and Conditional Medium (CM) Treatment

The cell co‐culture system was constructed by using the Transwell method with 6‐well transwell chambers of 0.4 µm pore size (Corning, USA). Depending on the purpose of the experiment, different cells were added to the upper or lower chambers. For CM collections, NF, CAF, NIH3T3‐LV‐VEC, and NIH3T3‐LV‐VTN were cultured until reaching a cell density of 70%–80%. The medium was then changed to fresh complete medium and cultured for a further 24–48 h, filtered through 0.22 mm filters, and stored at −80°C until needed. The CM was mixed 1:1 with a complete medium for further cell culture.

### 2.20 Expression Plasmids, shRNA, and siRNA

VTN overexpression lentivirus was provided by Hanbio Co., Ltd.(Shanghai, China). pLenti‐CMV‐mVtn‐GFP‐Puro was obtained from the Public Protein/Plasmid Library (Nanjing, China) for the package of lentivirus and overexpression of mVtn. Small interfering RNAs (siRNAs) against VTN, SLC6A8, and Slc6a8 were synthesized by Genepharma (Shanghai, China), the sequences of which are listed in Table S4 (Supporting Information). For the establishment of VTN knockdown cells, LV3‐shVTN‐2 lentiviral particles were used.

### 2.21 Infections and Transfections

Stable cells were established by lentivirus infection and selected with puromycin. Empty vector was used as the control for overexpression and shRNA‐based knockdown. siRNA transfection was performed using Lipofectamine 3000 reagent (Thermo Fisher, USA) according to the manufacturer's instructions.

### 2.22 Stablishment and Induction of a Macrophage M2 Polarisation System

THP1: THP‐1 cells were induced to differentiate into M0 macrophages by adding 100 nM PMA (MedChemExpress, USA) for 48 hs. Afterward, different cell culture supernatants or cytokines were added based on the experimental requirements. To study the effect of CAF‐secreted VTN on the M2 polarisation of THP‐1 cells, we collected supernatants of CAF‐shNC and CAF‐shVTN separately. These were then combined with IL4 and IL13 (MedChemExpress, USA) and added to the medium of M0 macrophages to induce the transformation of THP‐1 macrophages into M2‐like macrophages (THP‐1‐derived TAMs). To investigate the impact of rhVTN on M2 polarisation of THP‐1‐derived TAMs, we added rhVTN (5 mg mL^−1^) + IL4 (20 ng mL^−1^) + IL13 (20 ng mL^−1^) to the rhVTN group, IL4 (20 ng mL^−1^) + IL13 (20 ng mL^−1^) to the positive control group, and an equal volume of PBS to the blank control group. After 24 h, we collected the cells.

RAW264.7: A concentration of 20 ng mL^−1^ IL4 (MedChemExpress, USA) was employed to induce the transformation of RAW264.7 macrophages into M2‐like macrophages (RAW264.7‐derived TAMs). Subsequently, cell supernatants from NIH3T3‐LV‐VEC and NIH3T3‐LV‐VTN were collected and cultured with RAW264.7 cells either by co‐culturing the two via a Transwell or by directly adding rmVTN to the RAW264.7 cells. After 24 h of culture, the cells were harvested.

BMDM: The BMDM cells were harvested and induced to become mature macrophages by treatment with M‐CSF (MedChemExpress, USA) for 7 days. The transformation of BMDM into M2‐like macrophages (BMDM‐derived TAMs) was induced by the addition of 20 ng mL^−1^ IL4. Then, cell supernatants from NIH3T3‐LV‐VEC and NIH3T3‐LV‐VTN were collected and cultured. This was done either by co‐culturing the two through Transwell chambers or by directly adding rmVTN to BMDM cells. The cells were then harvested after 24 h of continuous culture.

### 2.23 Bone Marrow‐Derived Macrophage Isolation and Culture

The femurs and tibiae of male C57BL/6J mice were collected, sterilized, and cleaned. The bone marrow was extracted from the femurs and tibiae using a 1 mL syringe and pre‐cooled induction medium (RPMI‐1640 complete medium containing 20 ng mL^−1^ of M‐CSF). Single‐cell suspensions were obtained by filtering the cells through a 70 µm cell filter. The suspensions were then transferred by centrifugation at 1200 rpm for 5 min, the supernatants were discarded, and the cells were resuspended and plated. The cell culture medium containing M‐CSF was replaced every 2–3 days during cell culture.

### 2.24 Detection of Creatine

For cell samples, we digested the target cells into single‐cell suspensions and transferred 1 × 10^6^ cells from each group into EP tubes. The cells were then broken down by ultrasound, followed by centrifugation to collect the cell supernatant. As for tissue samples, we weighed the same amount of sample for each group, thoroughly ground it, and then centrifuged it to collect the supernatant of the tissue homogenate. The creatine levels in the cells or tissues were then determined using the Creatine Assay Kit (BioAssay Systems, USA) according to the instructions. Briefly, 10 µL of each sample was transferred to a clear 96‐well plate. Then, 90 µL of assay buffer (90 µL Assay Buffer + 1 µL Enzyme A + 1 µL Enzyme B + 1 µL Dye Reagent) was added, and the plate was incubated for 30 min at room temperature. The absorbance OD value of each well was measured at 570 nm, and the corresponding creatine concentration was calculated from a standard curve.

### 2.25 Detection of ATP

For cell samples, the target cells are digested into a single‐cell suspension and transferred to an EP tube at a concentration of 1 × 10^5^ cells/100 µL. For tissue samples, 20 mg of the sample is weighed, ground thoroughly, and centrifuged. The supernatant of the tissue homogenate is then collected.  ATP concentrations in the cells or tissues were then measured using an ATP Assay Kit (BioAssay Systems, USA) according to the instructions. Briefly, 10 µL of each sample is transferred to a white opaque 96‐well plate. Then, 90 µL of working solution (95 µL Assay Buffer + 1 µL substrate + 1 µL ATP Enzyme) is added to each well. The luminescence value should be read on the luminometer immediately after adding the working solution. Finally, the corresponding ATP concentration can be calculated according to the standard curve.

### 2.26 Statistical Analysis

Statistical analysis was conducted using GraphPad Prism 8.0 or SPSS 19.0. Student's t‐tests or one‐way ANOVA tests were used to examine statistical differences among assay groups when appropriate. The association between VTN expression and clinicopathological parameters in CRC patients was examined using the χ2 test. Continuous variables were presented as mean±standard deviation (SD). Survival analysis was performed using the Kaplan‐Meier method and the Log‐rank (Mantel‐Cox) test. The Cox proportional hazards regression model was used to conduct univariate and multivariate analyses. (*, P < 0.05; **, P < 0.01; ***, P < 0.001; and NS means not significant).

## 3 Results

### 3.1 Multi‐Database Joint Screening of Highly Expressed Genes in Colorectal Cancer Cafs

**Figure 1:**
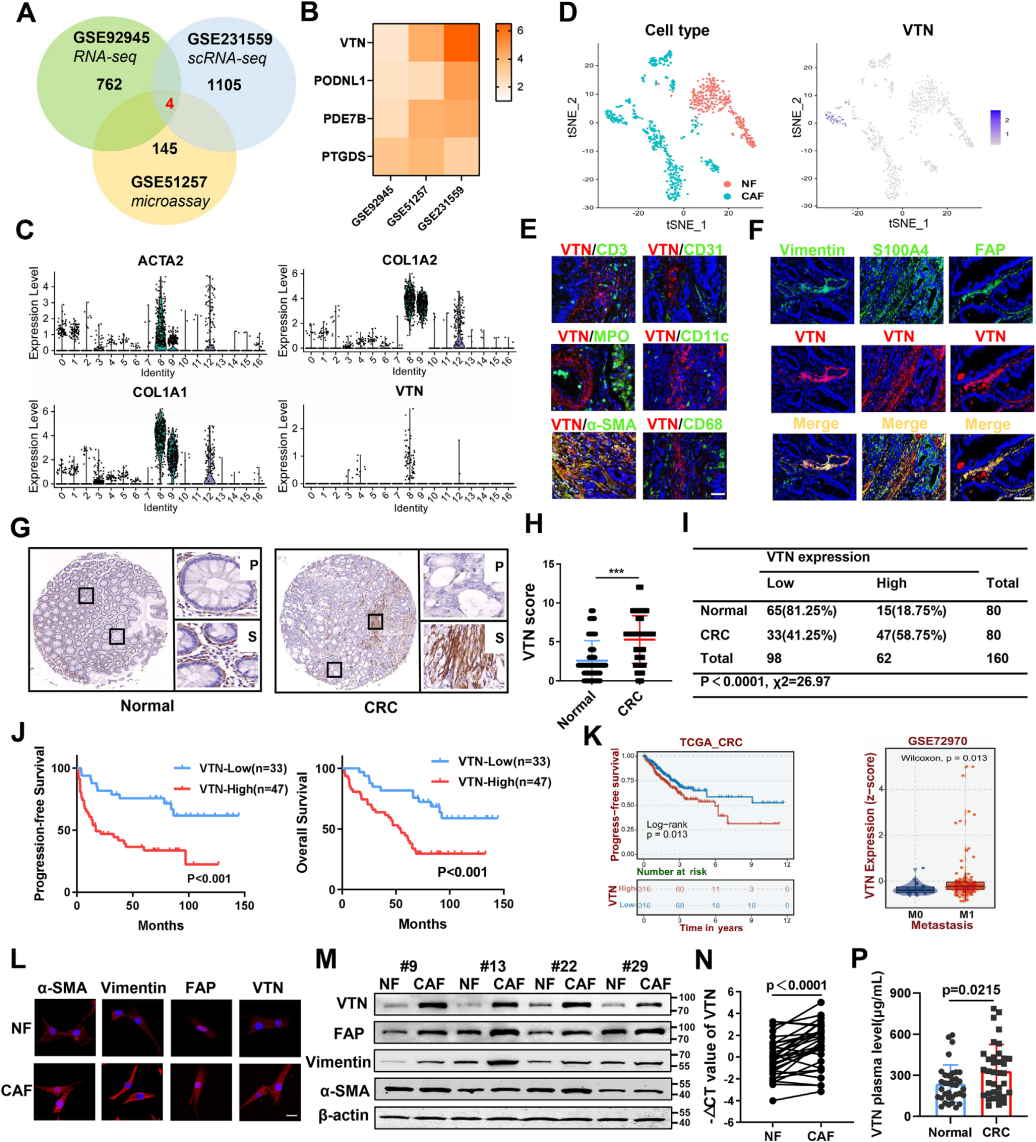
Vitronectin Significantly Up‐regulated in CAFs Correlates with Poor Clinical Outcomes in CRC Patients A) Venn diagram demonstrating the intersection of upregulated differentially expressed genes (DEGs) in cancer‐associated fibroblasts (CAFs) compared to normal fibroblasts (NFs), as identified in GSE51257, GSE92945, and GSE231559 datasets. B) Heatmap showing the expression patterns of the overlapping genes across the three datasets. C) Violin plots illustrating the expression levels of fibroblast marker genes (ACTA2, COL1A1, COL1A2) and VTN within each cluster, based on a single‐cell RNA sequencing dataset (GSE231559) comprising 6 CRC tissues and 3 adjacent normal tissues. D) tSNE plot of total fibroblasts isolated and re‐clustered from GSE231559, color‐coded by sample origin (left) and VTN expression (right). E)  Representative images of immunofluorescence costaining for VTN (red) with cell‐type markers (green) in CRC tissues from patients. Scale bars: 50 µm. F) Representative images of immunofluorescence co‐staining for VTN (red) with Vimentin, S100A4, and FAP (green) in CRC tissues from patients. Scale bars: 100 µm. G) Representative immunohistochemical staining of VTN in CRC tissue microarray. Magnification: 10X (left) and 200X (right). P: Parenchyma; S: Stroma. H) Immunohistochemical Scoring of VTN in CRC tissues and adjacent normal tissues. Student's t‐test. ***p<0.001. I) Classification of CRC and adjacent tissues into high and low VTN expression groups, followed by Chi‐Square test analysis. p<0.0001, χ2 = 26.97. J) Kaplan‐Meier survival analysis of the impact of VTN expression intensity on progression‐free survival (PFS, left) and overall survival (OS, right) of CRC patients. n = 33 patients for VTN‐low group; n = 47 patients for VTN‐high group. Log‐rank test. p<0.001. K) Higher VTN expression is associated with poorer PFS in The Cancer Genome Atlas (TCGA) CRC cohort (left), and increased distant metastasis rates in the GSE72970 CRC cohort (right). L,M) Verification of CAFs and NFs isolated from CRC tissues and paired noncancerous tissues by immunofluorescent staining (L) and western blot (M) for α‐SMA, Vimentin, FAP, and VTN, with higher expression observed in CAFs compared to NFs. Scale bar: 100 µm. N) qRT‐PCR analysis of VTN mRNA levels in 34 pairs of NFs and CAFs isolated from CRC patients. Paired two‐tailed Student's t‐test was used to calculate p value. P) ELISA detection of VTN expression in plasma samples from CRC patients (n = 36) and healthy individuals (n = 35). Student's t‐test was used to calculate p value. CAFs, cancer‐associated fibroblasts; NF, normal fibroblasts; t‐SNE, t‐distributed stochastic neighbor embedding;  CRC, colorectal cancer.

In search of genes specifically highly expressed in CAFs, we first analyzed differential gene expression profiles between NFs and CAFs isolated from CRC tissues using datasets GSE51257 and GSE92945 (Figure S1A, Supporting Information). Subsequently, utilizing scRNA‐seq data from dataset GSE231559, we characterized NF and CAF cell subpopulations and performed differential gene expression analysis. Finally, through intersection analysis of these differential gene sets, we identified VTN as a gene prominently upregulated in CAFs (**Figure** **1**A,B).

Analysis of the scRNA‐seq dataset from GSE231559 delineated 17 clusters, with subclusters 8, 9, and 12 (C8, C9, C12) classified as fibroblast cells (ACTA2, COLA1, and COLA2 positive), and C8 showed enriched expression of VTN (Figure 1C; Figure S1B, Supporting Information). Subsequently, all fibroblasts underwent isolation and dimensionality reduction clustering to classify them according to clusters and sample origins. Results from t‐SNE plot and gene expression profiling revealed significantly higher VTN expression in CAFs compared to NFs, with nearly negligible expression in NFs (Figure 1D; Figure S1C, Supporting Information). To further elucidate the cellular origin of secreted VTN within the TME, we conducted colocalization analysis using tumor tissue sections from CRC patients. Our findings revealed a distinct colocalization of VTN with α‐SMA, a marker of fibroblasts, while no significant overlap was observed with CD3 (T lymphocyte marker), CD31 (endothelial marker), myeloperoxidase (neutrophil marker), CD11c (dendritic cell marker), or CD68 (macrophage marker). These results suggest that CAFs are the primary cellular source of secreted VTN within the TME (Figure 1E). Furthermore, analysis using the TIMER 2.0 database, encompassing various immune infiltration algorithms, consistently demonstrated a high correlation between VTN and CAF infiltration in CRC across multiple modes of analysis (Figure S1D, Supporting Information).

### 3.2 Localization and Prognostic Significance of VTN in Colorectal Cancer Patients

Following a comprehensive database analysis and validation of tumor tissue samples from CRC patients, we identified VTN as highly expressed in CAFs. Notably, Vimentin, S100A4, and FAP were identified as CAF markers, primarily localized in the stromal tissues of tumors and co‐localizing with VTN (Figure 1F). Tissue microarrays of CRC specimens further confirmed elevated VTN expression in tumor stroma, contrasting with low expression in tumor parenchyma and paraneoplastic tissues in most cases (Figure 1G–I). Western blot analysis corroborated these findings, showing low VTN expression in the majority of CRC cell lines, except for Caco2 (Figure S1E, Supporting Information). High VTN expression correlated with worse progression‐free survival (PFS) and overall survival (OS) among CRC patients, along with relations to TNM stage, distant metastasis, and recurrence (Figure 1J; **Table** **1**). Cox regression analysis revealed high VTN expression and distant metastasis as independent factors affecting OS and PFS in CRC patients (**Table** **2**). Furthermore, elevated VTN expression was correlated with poorer PFS, increased rates of distant metastasis, and higher clinical stage (Figure 1K; Figure S1F, Supporting Information).

**Table 1:** The relationship between VTN expression and clinicopathological features in patients with CRC (n = 80).

Variable	VTN expression		P value
	Low expression(n = 33)	High expression(n = 47)	
Age (mean±SD)	60.24±14.22	58.23±12.709	0.504
Gender			
Male	18(54.5%)	26(55.3%)	0.945
Female	15(45.5%)	21(44.7%)	
Differentiation grade			
Highly	2(6.1%)	4(8.5%)	
Moderately	26(78.8%)	42(89.4%)	0.813
Poorly	5(15.2%)	1(2.1%)	0.099
Tumor size (cm)			
<5	16(48.5%)	29(61.7%)	0.242
≥5	17(51.5%)	18(38.3%)	
TNM stage			
I‐II	20(60.6%)	11(23.4%)	0.001
III‐IV	13(39.4%)	36(76.6%)	
T stage			
T1‐2	7(21.2%)	8(17.0%)	0.637
T3‐4	26(78.8%)	39(83.0%)	
Lymphnode metastasis			
No	23(69.7%)	23(48.9%)	0.067
Yes	10(30.3%)	24(51.1%)	
Metastasis			
No metastasis	27(81.8%)	28(59.6%)	0.039
Distant metastasis	6(18.2%)	19(40.4%)	
Recurrence			
No recurrence	22(66.7%)	17(36.2%)	0.008
Local recurrence	11(33.3%)	30(63.8%)	
Neoadjuvant therapy			
No	13(39.4%)	27(57.4%)	0.114
Yes	20(60.6%)	20(42.6%)	
Preoperative CEA			
<2.5 ng ml^−1^	9(27.3%)	15(31.9%)	0.656
≥2.5 ng ml^−1^	24(72.7%)	32(68.1%)	

**Table 2:** Univariate and multivariate analysis of OS and PFS in patients with CRC.

	OS				PFS			
Characteristic	Univariate Analysis		Multivariate Analysis		Univariate Analysis		Multivariate Analysis	
	HR (95%CI)	P	HR (95%CI)	P	HR (95%CI)	P	HR (95%CI)	P
Age	1.004 (0.982‐1.027)	0.713			0.990 (0.968–1.013)	0.411		
Gender								
Male	1.000				1.000			
Female	1.276 (0.710‐2.291)	0.415			1.055 (0.571‐1.951)	0.864		
Differentiation grade								
Highly	1.000				1.000			
Moderately	4.984 (0.683‐36.343)	0.113			5.299 (0.724‐38.760)	0.101		
Poorly	4.974 (0.555‐44.554)	0.152			2.886 (0.261‐31.966)	0.388		
Tumor size (cm)								
<5	1.000				1.000			
≥5	0.798 (0.439‐1.451)	0.460			1.068 (0.578‐1.976)	0.833		
TNM stage								
I‐II	1.000				1.000			
III‐IV	12.641 (4.490‐35.588)	<0.001			10.096 (3.892‐26.188)	<0.001		
T stage								
T1‐2	1.000				1.000			
T3‐4	2.397 (0.945‐6.080)	0.066			4.287 (1.321‐4.287)	0.015		
Lymphnode metastasis								
No	1.000				1.000			
Yes	2.366 (1.306‐4.286)	0.004			2.174 (1.170‐4.040)	0.014		
Metastasis								
No metastasis	1.000		1.000		1.000		1.000	
Distant metastasis	9.186 (4.840‐17.434)	<0.001	6.737 (2.818‐16.107)	<0.001	9.731 (4.894‐19.347)	<0.001	7.179 (2.763‐18.652)	<0.001
Neoadjuvant therapy								
No	1.000				1.000			
Yes	0.784 (0.436–1.410)	0.417			0.861 (0.465–1.594)	0.634		
Preoperative CEA								
<2.5 ng ml^−1^	1.000				1.000			
≥2.5 ng ml^−1^	2.401 (1.117‐5.165)	0.025			1.701 (0.832‐3.478)	0.145		
VTN expression								
Low	1.000		1.000		1.000		1.000	
High	3.034 (1.555‐5.921)	0.001	2.391 (1.170‐4.889)	0.017	3.266 1.619‐6.589)	0.001	3.241 (1.477‐7.111)	0.003

Fresh tumor tissues and corresponding paracancerous tissues from 34 CRC patients were collected to isolate NFs and CAFs (Figure S1G, Supporting Information). Cellular immunofluorescence and western blot results confirmed higher expression of VTN and related markers in CAFs compared to NFs (Figure 1L,M). Additionally, both mRNA levels and the cell supernatant concentration of VTN were significantly higher in CAFs (Figure 1N; Figure S1H, Supporting Information). Analysis of plasma samples revealed significantly higher VTN expression in CRC patient plasma (331.56 ± 32.25 µg/mL) compared to the healthy control group (237.79 ± 23.08 µg/mL) (P = 0.0215) (Figure 1P). The findings suggest that high plasma expression of VTN may serve as a potential risk factor for CRC patients and a valuable biomarker for diagnosis and prognosis.

### 3.3 VTN Promotes Proliferation and Metastasis of Colorectal Cancer Cells

**Figure 2:**
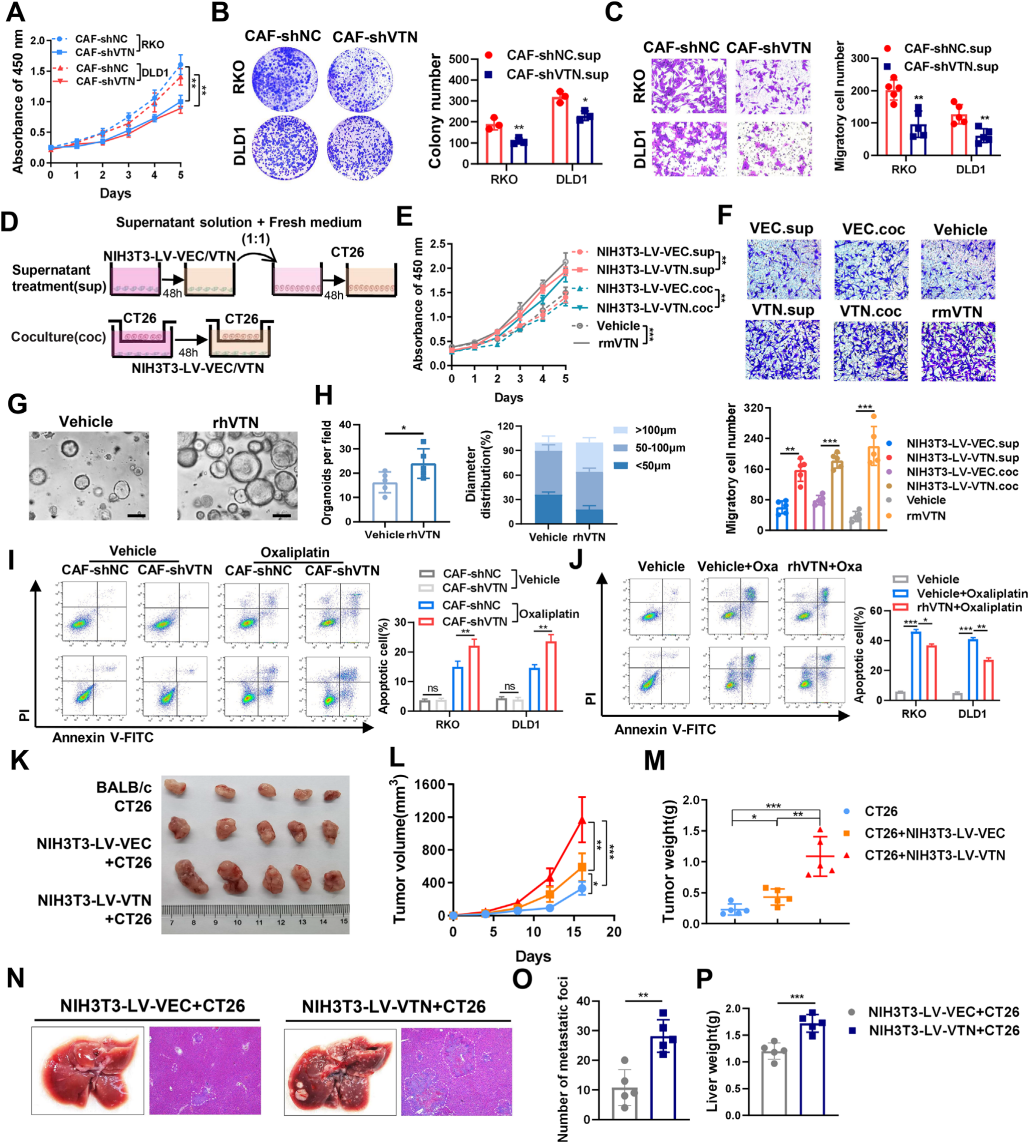
VTN Promotes Proliferation and Metastasis of Colorectal Cancer Cells A,B,C) The effects of CAF‐shNC and CAF‐shVTN conditioned media on proliferation, colony formation, and migration of RKO and DLD1 cells were observed through CCK‐8 assay(A), colony formation assay(B), and transwell assay (C), respectively. D) Schematic representation of the supernatant treatment and cell co‐culture model. (E,F) CCK‐8 assay E) and transwell assay F) were used to examine the effects of co‐culturing with NIH3T3‐LV‐VEC and NIH3T3‐LV‐VTN cells, supernatant treatment, and exogenous addition of rmVTN (5 µg mL^−1^) on proliferation and migration of CT26 cells. (G,H) Patient‐derived tumor organoids (PDTO) were isolated from CRC tissue and treated with vehicle or rhVTN (5 µg mL^−1^). G) Representative images of organoids. scale bar, 100 µm. H) Number of organoids (left) and proportion of different diameter classes of organoids (right) in the indicated two groups. I) Flow cytometry analysis of apoptosis levels in oxaliplatin‐induced RKO and DLD1 cells cultured with CAF‐shNC or CAF‐shVTN conditioned media. J) Percentage of apoptotic cells in RKO and DLD1 cells after being cultured alone, treated with oxaliplatin alone or treated with rhVTN (5 µg mL^−1^) and oxaliplatin. (K,L,M) CT26 cells alone and CT26 cells together with NIH3T3‐LV‐VEC or NIH3T3‐LV‐VTN cells were diluted and subcutaneously implanted into BALB/c mice(n = 5/group). Photographs of subcutaneous tumors (K), tumor volume growth curves (L), and tumor weights (M). (N,O,P) CT26 and NIH3T3‐LV‐VEC or NIH3T3‐LV‐VTN cells were mixed and injected into the spleens of BALB/c mice to induce liver metastases (n = 5/group). Representative images and HE staining results of liver metastasis (N), number of liver metastatic nodules (O), and liver weights (P). The difference between groups was determined by a one‐way analysis of variance or a two‐sided Student's t‐test. *p<0.05; **p<0.01; ***p<0.001; ns, not significant. All experiments were conducted 3 times in triplicate.

To investigate the impact of VTN on CRC cells, we constructed VTN‐silenced CAFs by lentiviral infection (Figure S2A,B, Supporting Information). Subsequently, CRC cells cultured with CAF‐shVTN supernatants exhibited attenuated proliferation, clonogenicity, and migratory abilities (**Figure** **2**A–C). Conversely, direct supplementation of recombinant human VTN (rhVTN) enhanced these capabilities in CRC cells (Figure S2C–E, Supporting Information). Given detectable VTN expression in the tumor parenchyma of some CRC patients and the Caco2 cells, VTN levels were modulated directly in CRC cells (Figure S2F,G, Supporting Information). Consistently, cells with VTN overexpression exert pro‐carcinogenic effects, while VTN knockdown has the opposite effect (Figure S2H–M, Supporting Information). Furthermore, we overexpressed VTN in NIH3T3 fibroblasts and co‐cultured them with CT26 cells or collected supernatant to mimic CAF effects on CRC cells (Figure 2D; Figure S3A, Supporting Information). The function of recombinant mouse VTN protein (rmVTN) was also investigated. Results showed the promotion of proliferation and migration in CT26 cells by both NIH3T3‐LV‐VTN and rmVTN treatment compared to controls (Figure 2E,F). Additionally, CRC organoids isolated from fresh tumor tissues exhibited increased numbers and volumes following treatment with rhVTN or overexpression of VTN (Figure 2G,H; Figure S3B,C, Supporting Information). CAF has been demonstrated to facilitate resistance to tumor therapy via multiple pathways. Following oxaliplatin treatment, we observed an increase in apoptotic CRC cells in the CAF‐shVTN group compared to the CAF‐shNC group (Figure 2I). Conversely, direct supplementation of rhVTN reduced the proportion of apoptotic cells after oxaliplatin administration (Figure 2J). Similar results were also observed following treatment with 5‐FU and SN‐38 (Figure S3D–G, Supporting Information), suggesting a role for VTN in driving chemotherapy resistance in CRC.

In vivo investigations involved mixing NIH3T3‐LV‐VEC or NIH3T3‐LV‐VTN cells with CT26 cells and injecting them subcutaneously into BALB/c mice and BALB/c‐nu mice. The group co‐injected with NIH3T3‐LV‐VTN and CT26 exhibited the largest and heaviest tumor volume, followed by the NIH3T3‐LV‐VEC and CT26 group, and finally the CT26 cells alone group, with statistically significant differences observed between each pair of groups (Figure 2K–M and Figure S3H–J, Supporting Information). Interestingly, the differences in tumor volume and weight between groups were more pronounced in BALB/c mice than in nude mice, indicating that VTN may promote tumor development through synergistic effects with other immune microenvironment components. In a mouse liver metastasis model, the co‐injection group of NIH3T3‐LV‐VTN and CT26 also displayed a greater number of liver metastatic nodules and heavier liver weights compared to the control group (Figure 2N‐P).

These findings collectively suggest that VTN can promote the proliferation, migration, metastasis, and drug resistance of CRC cells both in vitro and in vivo, thereby contributing to the development of CRC.

### 3.4 Fibroblast‐specific Knockout of VTN Inhibits Colorectal Cancer Progression

**Figure 3:**
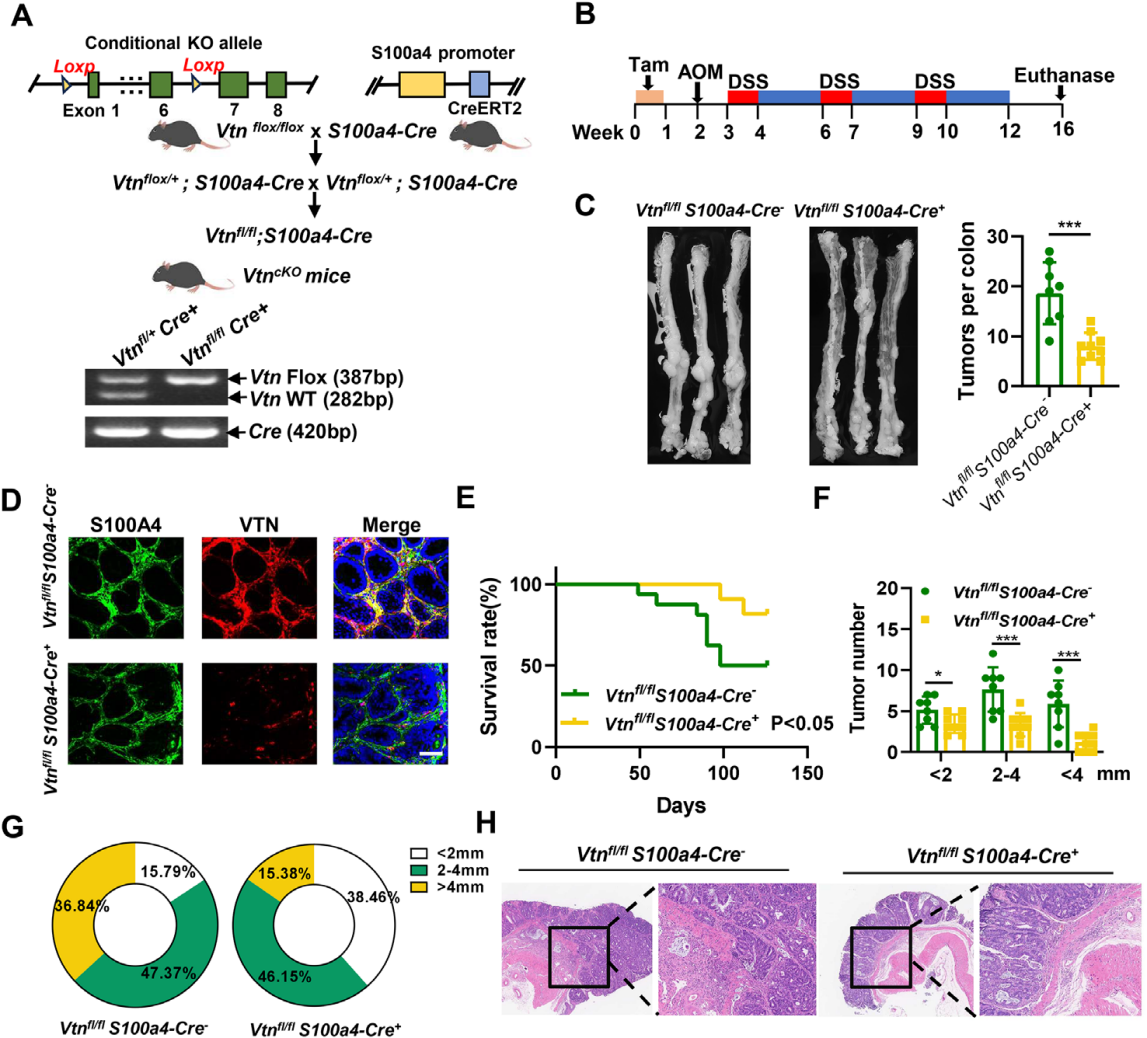
Fibroblast‐specific knockout of VTN inhibits colorectal cancer progression A) Schematic depicting the gene targeting strategy and crosses to generate VTN conditional knockout (*VTN^cKO^*) mice. Green boxes with numbers represent VTN exons, and yellow arrowheads indicate loxP sites. Upper: *Vtn^flox/flox^* (*Vtn^fl/fl^*) mice were crossed with *S100a4‐Cre* mice to produce *Vtn^fl/fl^ S100a4‐Cre^+^* mice. Lower: Genotyping PCR analysis using tail DNAs from *Vtn^fl/fl^ S100a4‐Cre^‐^* and *Vtn^fl/fl^ S100a4‐Cre^+^* mice. bp, base pair. B) Schematic illustration of the AOM/DSS‐induced model in *VTN^cKO^* mice. C) Representative images of tumor‐bearing colons(left) and tumor numbers(right) in *Vtn^fl/fl^ S100a4‐Cre^‐^* (n = 8) and *Vtn^fl/fl^ S100a4‐Cre^+^* group (n = 9). D) Immunofluorescence staining for S100A4 and VTN in intestinal tumor tissues from *Vtn^fl/fl^ S100a4‐Cre^‐^* and *Vtn^fl/fl^ S100a4‐Cre^+^* mice. scale bar, 50 µm. E) Survival curves using Kaplan‐Meier of the two groups. Log‐rank test. p<0.05. F) Measurement of tumor diameters and quantification of tumors by size(<2 mm, 2–4 mm, and >4 mm). G) Percentage of tumors of different sizes relative to the total in each group. H) Representative images of colonic tumor tissues of *Vtn^fl/fl^ S100a4‐Cre^‐^* and *Vtn^fl/fl^ S100a4‐Cre^+^* mice at the end of the experiments (left: 4X; right: 10X). The difference between the two groups was determined by two‐tailed Student's t‐test. *p<0.05; **p<0.01; ***p<0.001.

We investigated the role of CAFs‐derived VTN in CRC progression by generating transgenic mice with fibroblast‐specific knockout of VTN (*Vtn^fl/fl^ S100a4‐Cre^+^*) (**Figure** **3**A). S100A4 has been identified in numerous studies as a reliable marker for CAFs^[^ ^30^, ^31^, ^32^ ^]^, with expression observed in fibroblasts during post‐embryonic development up to day 8.5.^[^ ^33^, ^34^ ^]^ To closely mimic the pathogenesis and tumor microenvironment of human CRC, we established a tumor model in VTN conditional knockout (CKO) mice using the AOM/DSS chemical induction method (Figure 3B). Mice in the *Vtn^fl/fl^ S100a4‐Cre^+^* group demonstrated a notable reduction in both the number and size of colon tumors compared to the *Vtn^fl/fl^ S100a4‐Cre^‐^* group (Figure 3C). Immunofluorescence co‐staining confirmed the absence of VTN in the CAFs of the knockout mice (Figure 3D). However, this depletion did not significantly affect the number of SMA‐positive fibroblasts (Figure S4A, Supporting Information). Survival analysis using Kaplan‐Meier curves revealed that the *Vtn^fl/fl^ S100a4‐Cre^+^* group exhibited enhanced survival rates compared to the control group (Figure 3E). Additionally, tumor size categorization showed significantly fewer tumors measuring 2–4 mm and over 4 mm in the CAF‐specific VTN knockout group (Figure 3F,G).

Histological analysis via hematoxylin and eosin (H&E) staining revealed an increased proportion of high‐grade dysplasia and deeper tissue invasion in the colon of control mice, with extension into the submucosal or muscularis mucosae layers. In contrast, tumor development in the *Vtn^fl/fl^ S100a4‐Cre^+^* group was predominantly restricted to the mucosal layer (Figure 3H; Figure S4B, Supporting Information). These observations indicate that VTN knockout not only reduces tumor growth in the AOM/DSS model but also significantly mitigates the depth of tumor infiltration.

### 3.5 Impact of VTN on Polarization of Macrophages of Colorectal Cancer

**Figure 4:**
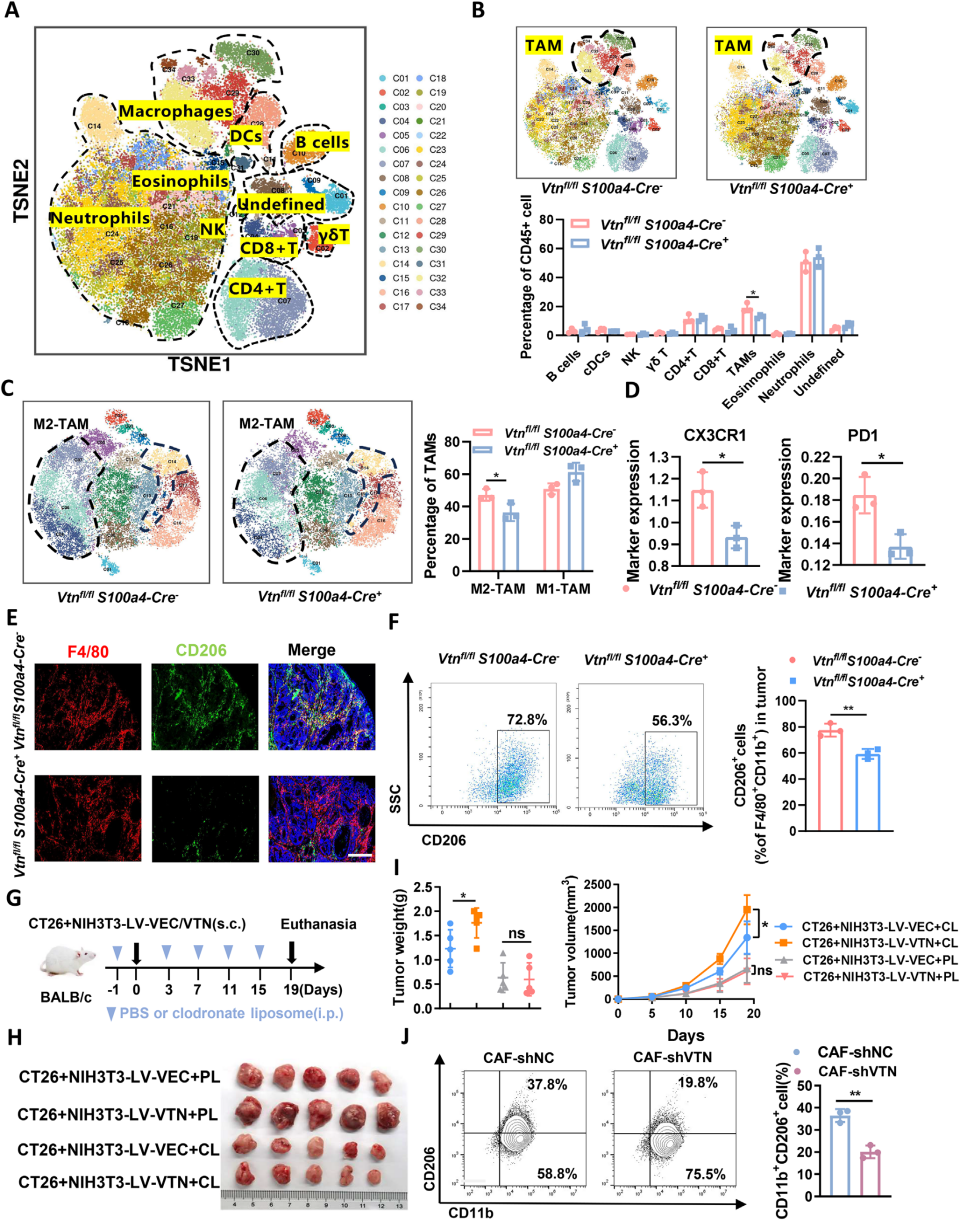
Impact of VTN on Polarization of Macrophages of Colorectal Cancer A) t‐SNE analysis of CyTOF data encompassing all CD45^+^ cells obtained from 6 AOM/DSS‐induced CRC tissues. B) Upper: t‐SNE analysis of CyTOF data representing immune cells from *Vtn^fl/fl^ S100a4‐Cre^‐^* and *Vtn^fl/fl^ S100a4‐Cre^+^* mice. Lower: The corresponding histogram quantifies the tumor‐infiltrating immune cells. C) Further extraction of TAMs followed by re‐clustering analysis, tSNE plots (left) and bar graphs (right) illustrate the differential distribution of M1‐TAMs and M2‐TAMs subclusters in the two groups. D) Expression box plot of CX3CR1 and PD1 expression level among TAMs. E) Representative images of immunofluorescence staining for F4/80 (red) and CD206 (green) in AOM/DSS‐induced CRC tissues obtained from *Vtn^fl/fl^ S100a4‐Cre^‐^* and *Vtn^fl/fl^ S100a4‐Cre^+^* mice. scale bar, 100 µm. F) Flow cytometry was used to quantify the percentage of M2‐TAMs in MC38 tumors between the *Vtn^fl/fl^ S100a4‐Cre^‐^* and *Vtn^fl/fl^ S100a4‐Cre^+^* groups. G,H,I) Subcutaneous tumor‐bearing mice were co‐injected with CT26 and NIH3T3 (LV‐VEC/LV‐VTN) cells and treated with either an PBS liposome (PL) or an clodronate liposome (CL) (n = 5/group). G) Schematic representation of the treatment plan. H) Representative images depicting tumors from different treatment groups. I) Quantification of tumor weight (left) and tumor growth (right). J) Flow cytometry analysis showing the proportion of CD206^+^CD11b^+^ THP‐1‐derived TAMs treatment with conditioned media from CAF‐shNC and CAF‐shVTN. The difference between groups was determined by one‐way ANOVA or two‐sided t‐test. *p<0.05; **p<0.01. CyTOF, mass cytometry by time of flight; TAMs, tumour‐associated macrophages.

To thoroughly explore the potential influence of VTN on the immune microenvironment of CRC, CyTOF analysis was utilized to detect differences in the immune cell composition within AOM/DSS‐induced CRC tissues between *Vtn^fl/fl^S100a4‐Cre^‐^* and *Vtn^fl/fl^S100a4‐Cre^+^* mice (Figure S5A,B, Supporting Information). A total of 34 cell subsets were identified, with the *Vtn^fl/fl^ S100a4‐Cre^+^* group showing a reduced proportion of tumor‐associated macrophages (TAMs) compared to the control group (**Figure** **4**A,B). Further extraction of macrophages for clustering analysis revealed a decrease in the percentage of M2‐TAMs in the *Vtn^fl/fl^ S100a4‐Cre^+^* mice (Figure 4C; Figure S5C,D, Supporting Information). CX3CR1 and PD1, recognized as markers of depleted macrophages closely involved in the pro‐tumor effects of M2‐TAMs, exhibited a discernible attenuation in the *Vtn^fl/fl^ S100a4‐Cre^+^* group. This observation suggests that VTN knockout may decrease the proportion of depleted M2‐TAMs, thereby mitigating the deleterious impact of the immunosuppressive TME (Figure 4D; Figure S5E, Supporting Information). Immunohistochemical validation confirmed a consistent reduction in M2‐TAMs within tumor tissues of *Vtn^fl/fl^ S100a4‐Cre^+^* mice (Figure 4E). Additionally, an examination of M2‐TAMs in tumor tissues co‐injected subcutaneously with CT26 cells and NIH3T3(LV‐VEC/LV‐VTN) cells indicated elevated F4/80 and CD206 expression in the VTN overexpression group (Figure S6A, Supporting Information).

To further explore the effect of VTN on macrophage M2 polarization, the proportion of M2 macrophages was assessed in subcutaneous tumors from VTN CKO mice. A marked reduction in the percentage of CD206+ macrophages was observed in the *Vtn^fl/fl^ S100a4‐Cre^+^* group compared to controls (Figure 4F and Figure S6B,C, Supporting Information). Moreover, to determine whether the pro‐tumorigenic influence of VTN in CRC is macrophage‐dependent, we depleted macrophages using clodronate liposomes. This intervention substantially attenuated the VTN‐driven promotion of CRC growth (Figure 4G–I and Figure S6D, Supporting Information).

Next, experiments using supernatants from CAF‐shNC and CAF‐shVTN revealed a lower proportion of CD206‐positive macrophages in the CAF‐shVTN group, indicating that VTN knockdown inhibits M2 polarization of THP‐1‐derived TAMs (Figure 4J). Meanwhile, the addition of rhVTN further increased the proportion of M2‐like macrophages (Figure S7A, Supporting Information). Furthermore, we employed bone marrow‐derived macrophage (BMDM) isolated from mice and induction by macrophage colony ‐ stimulating factor (M‐CSF) (Figure S7B, Supporting Information). BMDM‐derived TAMs were subsequently cultured in the supernatant of NIH3T3‐LV‐VEC and NIH3T3‐LV‐VTN cells, directly co‐cultured with them, or supplemented with exogenous rmVTN. Flow cytometry analysis showed a significant increase in F4/80+CD206+ M2‐like macrophages in the NIH3T3‐LV‐VTN or rmVTN‐treated groups (Figure S7C,D, Supporting Information). Further investigations using the murine monocyte‐macrophage cell line RAW264.7, involving qRT‐PCR, immunofluorescence staining, and flow cytometry, demonstrated that VTN presence promotes M2 polarization (Figure S7E–I, Supporting Information). These findings collectively suggest that reducing VTN expression in CAFs decreases the proportion of M2‐TAMs and highlights the role of VTN in macrophage polarization toward the M2 phenotype.

### 3.6 VTN Upregulates SLC6A8 Expression via the Integrin‐FAK Signaling Axis

**Figure 5:**
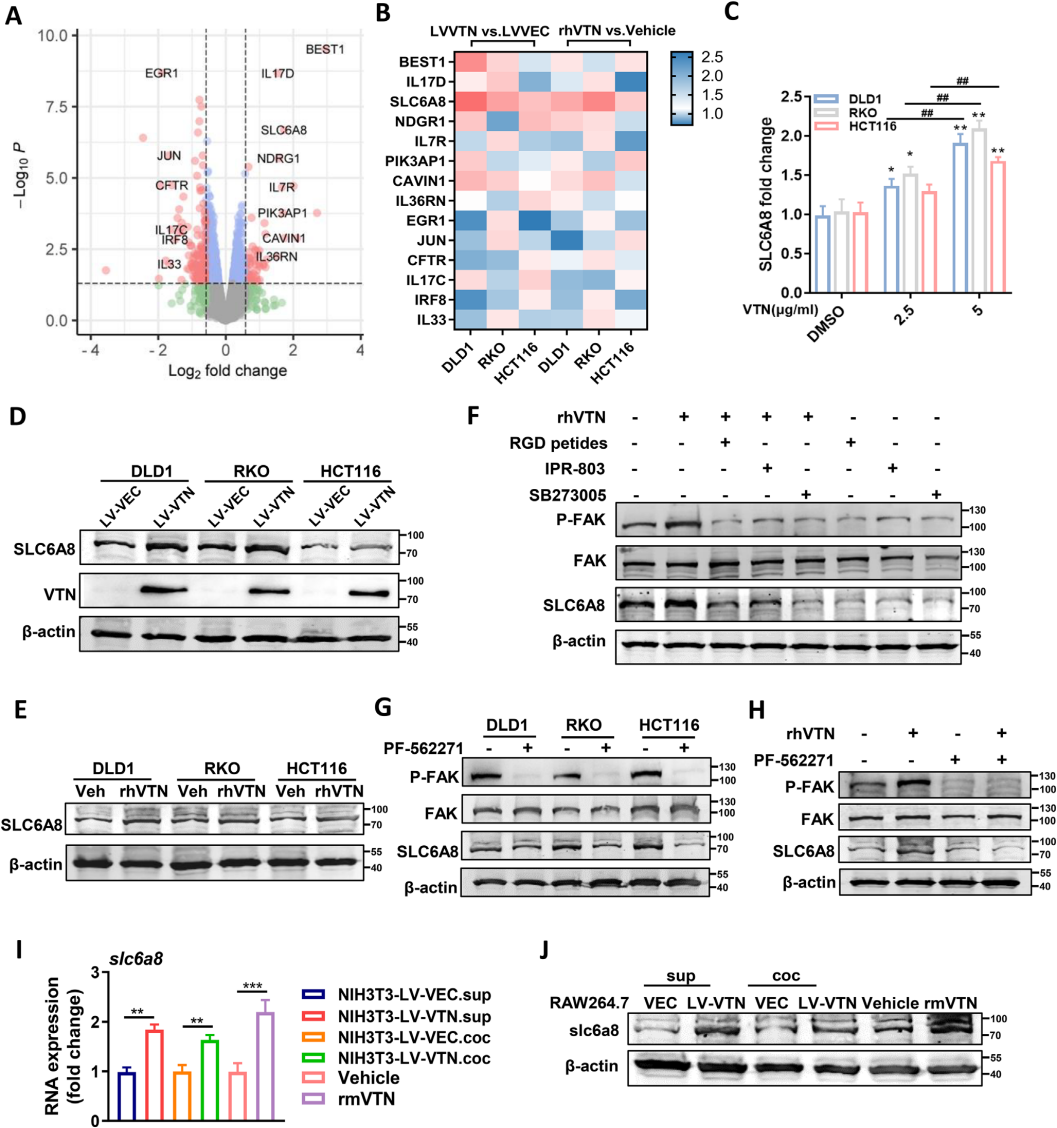
VTN Upregulates SLC6A8 Expression via the Integrin‐FAK Signaling Axis A) Volcanic plot depicting the differentially expressed genes between DLD1‐LV‐VEC and DLD1‐LV‐VTN cells through RNA‐seq analysis. B) Differential expression analysis of candidate genes via qRT‐PCR in CRC cell lines featuring VTN overexpression or supplemented with rhVTN. Heatmap visualization presents the relative mRNA fold changes. C) qRT‐PCR analysis of SLC6A8 mRNA expression in CRC cells exposed to varied concentrations of rhVTN (2.5 or 5 µg mL^−1^). The VTN concentration was selected based on CCK‐8 assay results demonstrating the most significant proliferation‐promoting effects in CRC cells. One‐way ANOVA. *p<0.05; **p<0.01.^##^P<0.01. D,E) SLC6A8 expression in CRC cell lines with VTN overexpression (D) and exogenous VTN supplementation (E) was examined by Western blot. F) Western blot analysis showing the impact of rhVTN (5 µg mL^−1^), RGD peptide (100 µM), integrin receptor inhibitor SB273005 (10 nM), and uPAR receptor inhibitor IPR‐803 (30 µM) on FAK phosphorylation and SLC6A8 expression in RKO cells following the indicated treatment. G) The effect of FAK inhibitors PF‐562271 (5 µM) on SLC6A8 expression was examined by western blot analysis. H) Western blot analysis was conducted to examine the FAK phosphorylation and SLC6A8 expression in RKO cells treated with control DMSO and PF‐562271 (5 µM) in the presence of PBS or rhVTN (5 µg mL^−1^). I,J) Evaluation of SLC6A8 expression in RAW264.7 macrophages following supernatant culture or co‐culture with NIH3T3‐LV‐VEC/VTN cells, and addition of rmVTN, using qRT‐PCR (I) and western blotting(J). Student's t‐test. **p<0.01; ***p<0.001. qRT‐PCR, quantitative real‐time PCR.

To delve deeper into the potential mechanisms driving the oncogenic effects of VTN, we employed RNA sequencing to explore differential gene expression profiles between VTN‐overexpressing and control groups (**Figure** **5**A). Follow‐up validation of 14 differentially expressed genes in CRC cells revealed that SLC6A8 exhibited the most significant upregulation in response to VTN overexpression and exogenous supplementation (Figure 5B). Solute carrier family 6 member 8 (SLC6A8) encodes a creatine transporter protein that modulates cellular creatine (Cr) uptake in a Na+/Cl‐ dependent manner.^[^ ^35^ ^]^ Higher concentrations of VTN can result in a more pronounced elevation of SLC6A8 mRNA levels (Figure 5C). Western blot analysis further corroborated the upregulation of SLC6A8 in response to VTN overexpression and supplementation (Figure 5D,E).

Previous literature indicates that VTN primarily binds to integrin receptors on the cell surface, activating focal adhesion kinase (FAK) and subsequent downstream signaling pathways and transcription factors.^[^ ^21^ ^]^ Additionally, the urokinase plasminogen activator receptor (uPAR) acts as a crucial signaling co‐receptor for integrins, facilitating the activation of downstream pathways.^[^ ^36^ ^]^ Our results revealed that rhVTN introduction increased FAK phosphorylation, concurrent with a rise in SLC6A8 expression. Conversely, the blockade of VTN binding with RGD peptide, integrin, and uPAR receptor inhibitors significantly reduced FAK phosphorylation and SLC6A8 expression (Figure 5F). Direct inhibition of the FAK signaling pathway with PF‐562271 also led to decreased SLC6A8 expression in CRC cells (Figure 5G), and this effect persisted even when rhVTN was supplemented, provided that FAK phosphorylation was inhibited (Figure 5H). Our findings elucidate the regulatory role of VTN in SLC6A8 expression through the integrin/uPAR–FAK signaling pathway.

Additionally, we observed that VTN‐mediated regulation of SLC6A8 expression extends to RAW264.7 macrophages. Co‐culturing with NIH3T3‐LV‐VTN cells, exposure to their supernatants, or supplementation with rmVTN, significantly upregulated SLC6A8 expression in macrophages relative to controls (Figure 5I,J). These results collectively demonstrate the broad impact of VTN on cellular function through modulation of the SLC6A8 pathway.

### 3.7 SLC6A8 Mediates VTN‐Induced Promotion of Oncogenesis and Macrophage Polarization through Enhanced Creatine Uptake

**Figure 6:**
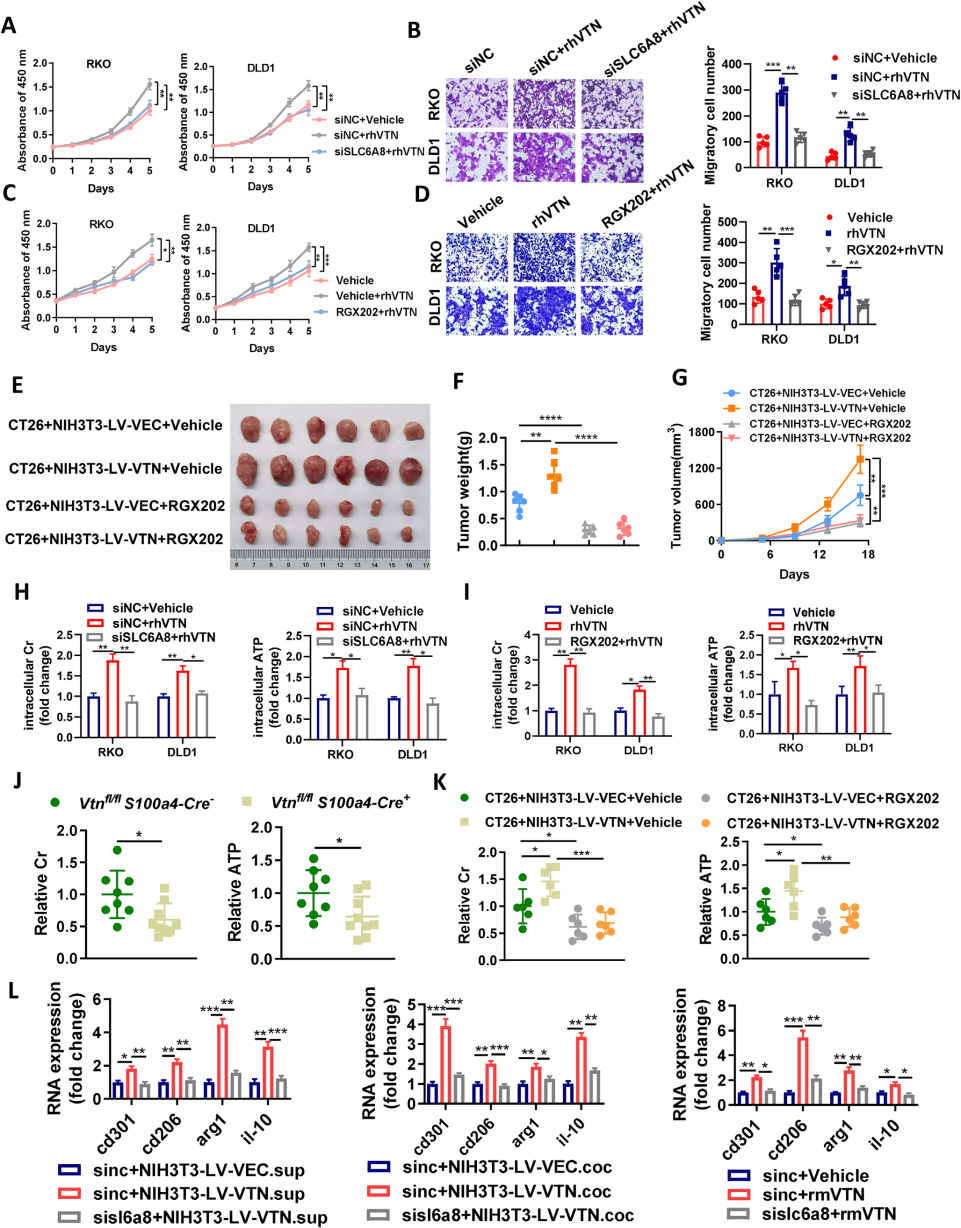
SLC6A8 Mediates VTN‐Induced Promotion of Oncogenesis and Macrophage Polarization through Enhanced Creatine Uptake A,B) CCK‐8 assays (A) and transwell migration assays (B) were conducted on RKO and DLD1 cells transfected with SLC6A8 siRNAs or mock controls, cultured in PBS or rhVTN. C,D) RKO and DLD1 cells were treated with vehicle or the SLC6A8 inhibitor RGX202 under conditions of PBS or rhVTN supplementation for CCK‐8 assays (C) and transwell migration assays (D). E,F,G) CT26 cells and NIH3T3‐LV‐VEC or NIH3T3‐LV‐VTN cells were subcutaneously co‐injected into the flank region of BALB/c mice (n = 6/group), and subjected to treatment with vehicle or RGX202. (E) Representative images of xenograft tumors of each group. Measurements of tumor weights (F), and growth curves (G) were recorded. H) Evaluation of intracellular creatine and ATP relative concentrations of RKO and DLD1 cells with mock controls or SLC6A8 knockdown (siSLC6A8) cultured in PBS or rhVTN. I) The relative concentrations of intracellular creatine and ATP in RKO and DLD1 cells were treated with vehicle or RGX202 in the presence of PBS or rhVTN. J) Relative levels of creatine and ATP in AOM/DSS‐induced tumors of *Vtn^fl/fl^ S100a4‐Cre^‐^* and *Vtn^fl/fl^ S100a4‐Cre^+^* mice were measured. K) Examination of relative levels of creatine and ATP in subcutaneous tumor tissues formed upon co‐injection of CT26 cells with NIH3T3‐LV‐VEC or NIH3T3‐LV‐VTN cells. L) qRT‐PCR analysis of M2 macrophage marker genes in RAW264.7 cells with or without SLC6A8 knockdown, under conditions of supernatant culture or co‐culture with NIH3T3‐LV‐VEC and NIH3T3‐LV‐VTN cells, supplemented with rmVTN. The difference between groups was determined by one‐way analysis of variance or two‐sided Student's t‐test. *p<0.05; **p<0.01; ***p<0.001.

To validate the role of SLC6A8 in mediating VTN‐induced oncogenic effects and M2 polarization, we initially conducted knockdown experiments targeting SLC6A8 expression in CRC cells (Figure S8A, Supporting Information). The results showed that SLC6A8 knockdown significantly reduced the proliferative and migratory effects of rhVTN treatment (**Figure** **6**A,B). Similarly, the introduction of SLC6A8 antagonist RGX202, which inhibits cell surface SLC6A8 receptors, effectively reversed the pro‐oncogenic effects of rhVTN (Figure 6C,D). Consistent results were observed when SLC6A8 was knocked down or RGX202 was administered in VTN‐overexpressing CRC cells (Figure S8B–E, Supporting Information). In vivo experiments further corroborated these findings, where RGX202 treatment in subcutaneous tumor‐bearing mice, co‐injected with CT26 and NIH3T3 (LV‐VEC and LV‐VTN) cells, led to a significant reduction in tumor volume and weight compared to control groups (Figure 6E–G).

SLC6A8 facilitates the transport of creatine into cells, where intracellular creatine can be phosphorylated by creatine kinase to form phosphocreatine, further synthesizing ATP. The cell culture medium was supplemented with 10% FBS, serving as the source of creatine. Both exogenous addition and overexpression of VTN significantly elevated intracellular creatine and ATP levels, while SLC6A8 knockdown or RGX202 treatment impeded this process (Figure 6H,I; Figure S8F,G, Supporting Information). Furthermore, intestinal tumors from the *Vtn^fl/fl^ S100a4‐Cre^+^* group exhibited lower creatine and ATP levels compared to those from the *Vtn^fl/fl^ S100a4‐Cre^‐^* group (Figure 6J). In a co‐injection subcutaneous tumor model, creatine and ATP concentrations were elevated in the CT26+NIH3T3‐LV‐VTN group but decreased following treatment with RGX202 (Figure 6K).

Our studies also assessed the effects of VTN on macrophage creatine and ATP levels by co‐culturing NIH3T3‐LV‐VEC and NIH3T3‐LV‐VTN cells with RAW264.7 macrophages, using cell supernatants for culture or supplemented with rmVTN. This setup resulted in enhanced intracellular creatine and ATP levels in the presence of VTN (Figure S8H, Supporting Information). Additionally, VTN exposure significantly increased the expression of M2 polarization markers (CD301, CD206, ARG1, and IL10) in macrophages, with a reduction observed upon SLC6A8 knockdown (Figure 6L and Figure S8I, Supporting Information). These findings indicate that VTN facilitates M2 macrophage polarization through a SLC6A8‐dependent mechanism that promotes creatine uptake.

### 3.8 Augmentation of PD‐1 Inhibitor Therapy Efficacy in Colorectal Cancer Through Targeted VTN Knockout in Fibroblasts

**Figure 7:**
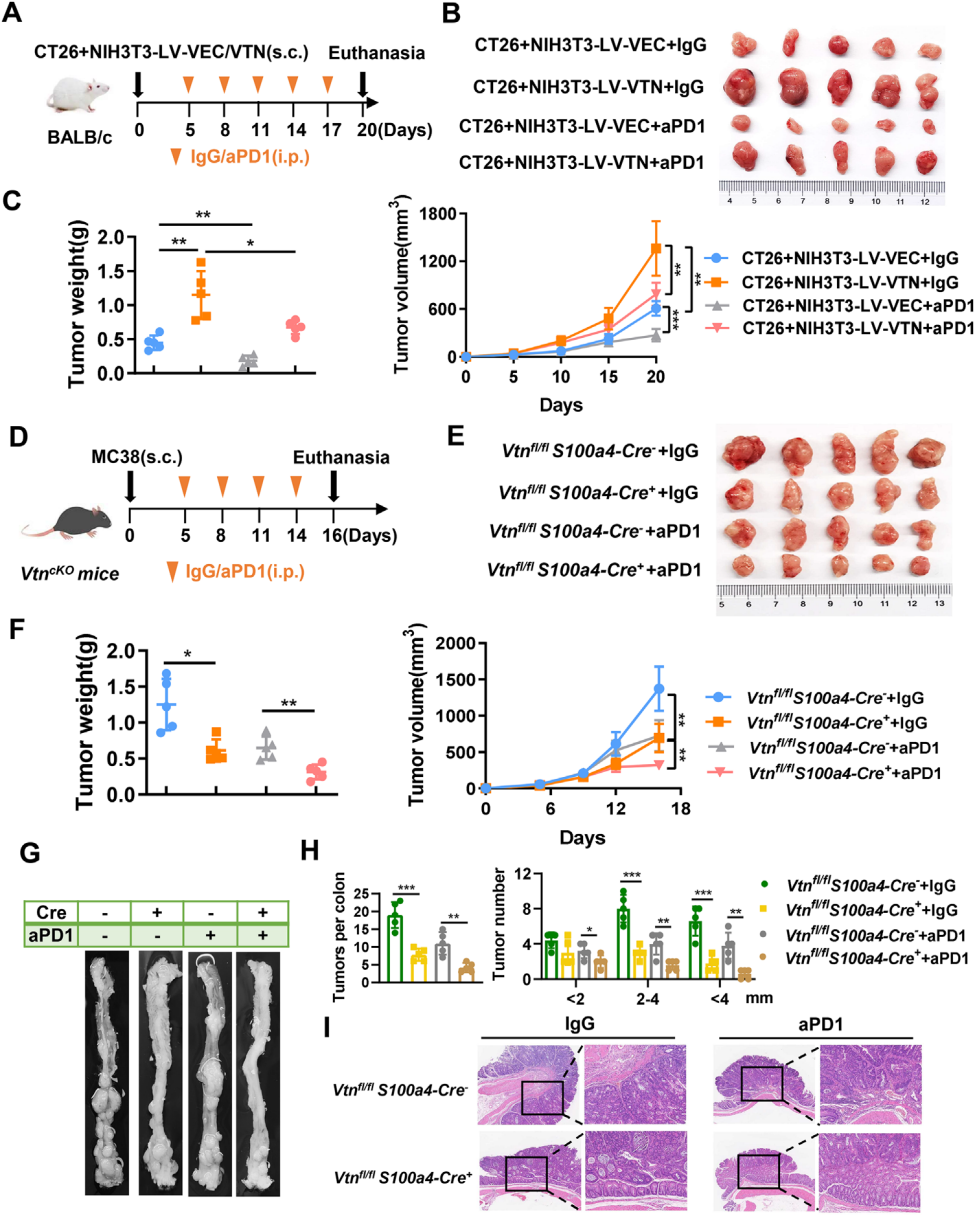
Augmentation of PD‐1 Inhibitor Therapy Efficacy in Colorectal Cancer Through Targeted VTN Knockout in Fibroblasts A,B,C) Subcutaneous tumor‐bearing mice were co‐injected with CT26 and NIH3T3 (LV‐VEC/LV‐VTN) cells and treated with either an isotype control (IgG) or an anti‐PD‐1 mAb (aPD1) (n = 5/group). A) Schematic representation of the treatment plan. B) Representative images depicting tumors from different treatment groups. C) Quantification of tumor growth (left) and tumor weight (right). D,E,F) Tumor growth of MC38 cells in *Vtn^fl/fl^ S100a4‐Cre^‐^* and *Vtn^fl/fl^ S100a4‐Cre^+^* mice treated with either an isotype control or an anti‐PD‐1 mAb was monitored (n = 5/group). D) Schematic representation of the treatment plan. E) Representative images illustrating tumors from various treatment groups. F) Measurement of tumor growth (left) and tumor weight (right). G,H,I) The AOM/DSS‐induced CRC model was performed on *Vtn^fl/fl^ S100a4‐Cre^‐^* and *Vtn^fl/fl^ S100a4‐Cre^+^* mice treated with either an isotype control or an anti‐PD‐1 mAb (n = 5/group). (G) The macroscopic appearance of the tumor‐bering colon is depicted for each treatment. (H) Tumor numbers in each group were counted (left), and tumor diameters were measured and categorized by size (<2 mm, 2–4 mm, and >4 mm) (right). I) Representative HE images showing tumor invasion in the mice with the indicated treatments. The difference between two groups was determined by two‐tailed Student's t‐test. *p<0.05; **p<0.01; ***p<0.001. s.c., subcutaneous; aPD1, anti‐programmed cell death protein 1; mAb, monoclonal antibody.

The advent of immunotherapy has revolutionized the treatment landscape for CRC. Our studies have shown that VTN secreted by CAFs not only enhances the proliferation and invasion of CRC cells but also facilitates M2 macrophage polarization, intensifying the immunosuppressive TME and accelerating tumor progression. Extensive research indicates that TAMs, crucial components of the TME, significantly influence the efficacy of programmed death‐1 (PD‐1) and programmed death ligand‐1 (PD‐L1) inhibitors.^[^ ^37^ ^]^ Consequently, we administered anti‐PD‐1 (aPD1) treatment to subcutaneous tumor‐bearing mice co‐injected with CT26 and NIH3T3 (LV‐VEC/LV‐VTN) cells (**Figure** **7**A). Our findings revealed that aPD1 therapy reduced tumor volume and weight. Importantly, the CT26+NIH3T3‐LV‐VTN group demonstrated a 12.93% lower tumor suppression rate than the CT26+NIH3T3‐LV‐VEC group (Figures 7B,C; Figure S9A, Supporting Information), indicating a relative resistance of VTN‐expressing tumors to aPD1 therapy in CRC.

Following the subcutaneous implantation of MC38 cells into VTN CKO mice, we treated the mice with aPD1. The combination of aPD1 treatment with the *Vtn^fl/fl^ S100a4‐Cre^+^* mice resulted in more pronounced reductions in tumor volume and weight than in the *Vtn^fl/fl^ S100a4‐Cre^‐^* group treated with aPD1 (Figure 7D‐F; Figure S9C, Supporting Information). Moreover, we evaluated the therapeutic impact of aPD1 in VTN CKO mice using the AOM/DSS‐induced method. VTN knockout or aPD1 treatment led to reductions in both the number and size of intestinal tumors. The *Vtn^fl/fl^ S100a4‐Cre^+^* group treated with aPD1 showed the most substantial tumor reductions, with statistically significant differences from the other groups (Figure 7G,H). HE staining further confirmed a decrease in tumor infiltration depth across the treated groups, with the *Vtn^fl/fl^ S100a4‐Cre^+^* +aPD1 group showing the most profound therapeutic effect (Figure 7I; Figure S9F, Supporting Information). Additionally, we analyzed the proportion of CD206+ TAMs within the TME across the aforementioned models (Figure S9B,D,E, Supporting Information). These results suggest that VTN knockout in fibroblasts could potentiate the efficacy of aPD1, highlighting VTN as a potential therapeutic target to enhance aPD1 therapy effectiveness in CRC.

## 4 Discussion

Beyond directly targeting neoplastic cells, burgeoning research is devoted to exploiting the therapeutic potential of targeting the TME. Among the heterogeneous cellular constituents of the tumor stroma, CAFs emerge as pivotal players, markedly shaping the oncogenesis and progression of tumors. Our investigation revealed VTN as a gene conspicuously overexpressed in CAFs within the stroma of CRC. Subsequent in vitro and in vivo assays have substantiated the dual functionality of VTN: directly driving the proliferation and metastasis of CRC, and concurrently promoting macrophage polarization toward the M2 phenotype—thus intensifying the immunosuppressive microenvironment. Hence, our study posits novel potential targets and combinatory therapeutic approaches focused on modulating the microenvironment to enhance CRC management.

Vitronectin (VTN) is a glycoprotein extensively distributed in the extracellular matrix and plasma, predominantly originating from the liver, with platelets, megakaryocytes, and monocytes/macrophages contributing to minor secretions.^[^ ^22^ ^]^ This study is the first to identify that VTN can also be secreted by CAFs in CRC, with the expression in CAFs being significantly higher than that in NFs. Elevated VTN levels in CRC serve as independent prognostic indicators for overall survival and recurrence, with concentrations in patient blood samples markedly higher than those in healthy controls. Prior research by Rebeca et al. has noted increased VTN expression in neuroblastoma stromal tissues, associated with poorer event‐free survival (EFS).^[^ ^38^ ^]^ Additionally, serum protein markers identified in clinical and animal model studies suggest the potential of VTN as an early biomarker for CRC.^[^ ^26^ ^]^ Yin et al. conducted a systematic proteomic analysis of stage I to IIIC rectal cancer tissues, revealing significantly upregulated VTN expression levels in metastatic CRC.^[^ ^39^ ^]^ Despite these insights, research into the role of VTN in CRC remains limited, with most findings derived from small‐scale clinical retrospective cohort studies. In contrast, our study extensively confirmed, through numerous in vitro and in vivo experiments, organoid models, and VTN CKO mice, the promoting effect of CAF‐derived VTN on CRC cell growth and metastasis.

Importantly, our findings elucidate that VTN plays a crucial role in driving the polarization of TAMs toward the M2 phenotype. M2‐type macrophages are known to promote tumor progression and suppress immune responses through a variety of mechanisms.^[^ ^37^ ^]^ In our experimental models, *Vtn^fl/fl^ S100a4‐Cre^+^* mice developed notably smaller and fewer colon tumors following AOM/DSS induction compared to controls. CyTOF analysis further revealed a reduced proportion of M2‐TAMs cells and decreased expression of exhaustion markers CX3CR1 and PD1 in the *Vtn^fl/fl^ S100a4‐Cre^+^* group. These results are consistent with the findings of Liza et al., who reported enrichment of M1 macrophage markers CD68 and CD163 in regions with elevated VTN expression within liver metastases of CRC.^[^ ^40^ ^]^ Similarly, Peng et al. found increased VTN secretion in IL‐4‐induced RAW264.7 macrophages, contrasting with its near absence following LPS/IFN‐γ treatment.^[^ ^41^ ^]^ While VTN alone did not significantly induce M2 polarization, we found it synergistically augmented the proportion of M2‐polarized cells in the presence of M2‐polarizing factors (IL4/IL13). Some studies have reported VTN as a pro‐inflammatory mediator, capable of inducing IL‐6 expression in female mouse microglia post‐stroke, whereas such effects were not observed in males.^[^ ^42^ ^]^ In addition, VTN supplementation exacerbated inflammation in BMDMs triggered by hyperosmolarity, leading to upregulation of mRNA expression of pro‐inflammatory cytokines TNF‐α, IL‐1β, and IL‐6.^[^ ^43^ ^]^ Conversely, no significant upregulation of IL‐1β was detected in THP‐1 cells induced by VTN.^[^ ^44^ ^]^ These divergent findings involving the pro‐inflammatory effects of VTN on macrophages, all of which are non‐oncological diseases and require specific conditions, are thought to promote up‐regulation of M1 marker expression mainly via the VTN‐integrin‐NF‐kB pathway.^[^ ^42^, ^43^ ^]^ We propose that in CRC, VTN promotes macrophage M2 polarization primarily through the upregulation of SLC6A8 via the VTN‐integrin‐FAK pathway. This discrepancy may be attributed to the various disease environments that modulate the specific signaling pathways activated by VTN.

S100A4 is widely recognized as a sensitive marker for fibroblasts and has been extensively used to generate fibroblast‐specific knockout models using the Cre‐loxP system.^[^ ^30^, ^45^, ^46^, ^47^, ^48^, ^49^ ^]^ In normal mice, S100A4 is considered to be fibroblast‐specific based on both genetic and protein evidence.^[^ ^50^ ^]^ However, recent studies have emphasized the heterogeneity of fibroblasts across different tumor types, and that no Cre driver line specific to CAFs is currently available. Even fibroblast‐specific Cre lines exhibit inherent complexities. While S100A4 has been reported in non‐fibroblast cell types, such as macrophages^[^ ^18^, ^51^ ^]^ this non‐absolute specificity is a characteristic shared by several other fibroblast markers, including α‐SMA/ACTA2, PDGFRA, and COL1A2.^[^ ^52^ ^]^ For instance, ACTA2‐Cre induces recombination in smooth muscle and myoepithelial cells, and PDGFRA‐Cre and COL1A2‐Cre are also expressed in neurons and osteoblasts, respectively.^[^ ^52^ ^]^One study observed that S100A4+ cells were predominantly found in stromal cells, with some overlap in tumor cells, but still classified them as an important CAF subtype.^[^ ^32^ ^]^ Similarly, our results demonstrate high expression of S100A4 in fibroblasts, with VTN exclusively co‐localizing with fibroblasts. Therefore, while the *Vtn^fl/fl^ S100a4‐Cre* model is not exclusively specific for fibroblasts, its use in this study is methodologically justified.

Our RNA‐seq analysis revealed a significant increase in SLC6A8 expression in response to VTN. SLC6A8 is an emerging oncogenic factor, with studies reporting its upregulation in various malignant tumors.^[^ ^53^, ^54^, ^55^ ^]^ Acting as a creatine transporter, SLC6A8 contributes to cancer progression by modulating the intracellular concentrations of creatine, phosphocreatine, and ATP, thereby influencing cellular energy dynamics and metabolic reprogramming in tumor cells.^[^ ^56^ ^]^ Kurth et al. demonstrated that RGX202, an inhibitor of SLC6A8 transporter, exerts anti‐tumor effects in diverse primary and metastatic CRC models, including patient‐derived xenografts.^[^ ^57^ ^]^ Our rescue experiments confirmed that, even with ongoing VTN treatment, significant reductions in creatine and ATP levels were observed alongside marked inhibition of CRC progression following the targeted suppression of SLC6A8 via siRNA or RGX202. The role of creatine in cancer is multifaceted, as it can either suppress tumor growth by modulating energy metabolism in cancer cells and CD8+T cells or facilitate tumor progression through conventional metabolic regulation and alternative mechanisms.^[^ ^58^, ^59^, ^60^, ^61^, ^62^ ^]^ Existing research consistently supports the involvement of creatine in CRC invasion and metastasis, suggesting that it promotes these processes by increasing intracellular phosphocreatine and ATP levels, or through the activation of the Smad2/3 pathway via MPS1.^[^ ^56^, ^61^ ^]^ Moreover, investigations have highlighted the crucial role of SLC6A8‐mediated extracellular creatine uptake in macrophage polarization.^[^ ^63^ ^]^ Creatine promotes M2 polarization by suppressing iNOS and enhancing arginase 1 expression, whereas specific loss of SLC6A8 markedly reduces creatine levels in macrophages, leading to the opposite effect.^[^ ^63^ ^]^ Our findings also suggest that downregulation of SLC6A8 expression in macrophages inhibits the expression of M2 polarization markers under VTN treatment, implicating VTN in promoting M2 polarization by upregulating SLC6A8 expression. However, modulation of SLC6A8 expression may represent only one of the mechanisms by which VTN influences macrophage polarization, with other mechanisms warranting further investigation.

Anti‐PD‐1/PD‐L1‐based therapies represent a widely employed approach in the treatment of cancer. Nevertheless, the response rate to these therapies in CRC remains modest.^[^ ^64^ ^]^ In our investigations, we observed that the targeted deletion of VTN in CAF markedly potentiated the therapeutic efficacy of PD‐1 inhibition in mouse models of both subcutaneous and AOM/DSS‐induced tumors. The efficacy of anti‐PD‐1/PD‐L1 therapy may be compromised by the polarization of TAMs toward M2‐type macrophages. M2‐TAMs facilitate tumor growth by releasing anti‐inflammatory cytokines and angiogenic factors that suppress anti‐tumor immune responses.^[^ ^65^ ^]^ Hence, we propose that targeting VTN may enhance the efficacy of anti‐PD‐1 therapy by inhibiting TAMs from polarizing toward M2‐type macrophages, thereby alleviating the pro‐cancer effects of the immunosuppressive microenvironment to some extent. The development of VTN‐targeted neutralizing antibodies may constitute a novel therapeutic avenue for CRC, with planned systematic preclinical evaluations to elucidate their mechanistic validation and translational potential in forthcoming studies.

In conclusion, our study has established that VTN is specifically and prominently expressed in the CAFs of CRC. By activating the integrin‐FAK signaling pathway, VTN significantly enhances SLC6A8 expression and creatine uptake, thereby facilitating CRC growth, metastasis, and M2 macrophage polarization. These findings illuminate a novel role for CAFs‐derived VTN in modulating tumor progression and the TME, offering novel insights that pave the way for innovative therapeutic strategies targeting CRC.

## Conflict of Interest

The authors declare no conflict of interest.

## Author Contributions

L.Y. and G.Y. were responsible for the concept and experimental design. Y.J. performed most of cell and animal experiments and analyzed the data. XM assisted cell biology and in vivo experiments. YJ and XM contributed to clinical sample collection. YS and CZ contributed to the bioinformatics analysis. YJ and LY wrote and edited the manuscript. All authors have read and approved the final manuscript. LY and GY are responsible for the overall content as the guarantor.

## Ethics approval

All human clinical samples used in this study were approved by the Medical Ethics Committees of Shanghai East Hospital, Tongji University (Granted Number: 2023–215). Informed consent was obtained from all participants involved in the study. All the animal experiments were conducted following the Guidelines on Care and Use of Laboratory Animals by the National Institutes of Health (NIH) and approved by the Institutional Animal Care and Use Committee at Tongji University (Granted Number: TJ‐HB‐LAC‐2023‐18).

## Supporting information



Supporting Information

Supporting Information

Supporting Information

Supporting Information

Supporting Information

Supporting Information

Supporting Information

Supporting Information

Supporting Information

Supporting Information

## Data Availability

The data that support the findings of this study are available in the supplementary material of this article.

## References

[1] E. Morgan , M. Arnold , A. Gini , V. Lorenzoni , C. J. Cabasag , M. Laversanne , J. Vignat , J. Ferlay , N. Murphy , F. Bray , Gut 2023, 72, 338. 36604116 10.1136/gutjnl-2022-327736

[2] C. C. Murphy , T. A. Zaki , Nat. Rev. Gastroenterol. Hepatol. 2024, 21, 25. 37723270 10.1038/s41575-023-00841-9

[3] L. H. Biller , D. Schrag , JAMA, J. Am. Med. Assoc. 2021, 325, 669. 10.1001/jama.2021.0106 33591350

[4] E. Van Cutsem , A. Cervantes , R. Adam , A. Sobrero , J. H. Van Krieken , D. Aderka , E. A. Aguilar , A. Bardelli , A. Benson , G. Bodoky , F. Ciardiello , A. D'Hoore , E. Diaz‐Rubio , J. Y. Douillard , M. Ducreux , A. Falcone , A. Grothey , T. Gruenberger , K. Haustermans , V. Heinemann , P. Hoff , C. H. Köhne , R. Labianca , P. Laurent‐Puig , B. Ma , T. Maughan , K. Muro , N. Normanno , P. Österlund , W. J. G. Oyen , et al., Ann. Oncol. 2016, 27, 1386. 27380959 10.1093/annonc/mdw235

[5] D. T. Le , J. N. Uram , H. Wang , B. R. Bartlett , H. Kemberling , A. D. Eyring , A. D. Skora , B. S. Luber , N. S. Azad , D. Laheru , B. Biedrzycki , R. C. Donehower , A. Zaheer , G. A. Fisher , T. S. Crocenzi , J. J. Lee , S. M. Duffy , R. M. Goldberg , A. de la Chapelle , M. Koshiji , F. Bhaijee , T. Huebner , R. H. Hruban , L. D. Wood , N. Cuka , D. M. Pardoll , N. Papadopoulos , K. W. Kinzler , S. Zhou , T. C. Cornish , et al., N. Engl. J. Med. 2015, 372, 2509. 26028255 10.1056/NEJMoa1500596 PMC4481136

[6] M. J. Overman , R. McDermott , J. L. Leach , S. Lonardi , H.‐J. Lenz , M. A. Morse , J. Desai , A. Hill , M. Axelson , R. A. Moss , M. V. Goldberg , Z. A. Cao , J.‐M. Ledeine , G. A. Maglinte , S. Kopetz , T. André , Lancet Oncol. 2017, 18, 1182. 28734759 10.1016/S1470-2045(17)30422-9 PMC6207072

[7] J. N. Kather , N. Halama , D. Jaeger , Semin. Cancer Biol. 2018, 52, 189. 29501787 10.1016/j.semcancer.2018.02.010

[8] L. Chen , X. Jiang , Y. Li , Q. Zhang , Q. Li , X. Zhang , M. Zhang , Q. Yu , D. Gao , Clin. Immunol. 2022, 237,, 108962. 35227870 10.1016/j.clim.2022.108962

[9] B. Cheng , Q. Yu , W. Wang , J Biomed Sci 2023, 30, 1. 36600243 10.1186/s12929-022-00894-z PMC9814473

[10] G. Caligiuri , D. A. Tuveson , Cancer Cell 2023, 41, 434. 36917949 10.1016/j.ccell.2023.02.015 PMC11022589

[11] P. Aurello , G. Berardi , D. Giulitti , A. Palumbo , S. M. Tierno , G. Nigri , F. D'Angelo , E. Pilozzi , G. Ramacciato , Surgeon 2017, 15, 329. 28629870 10.1016/j.surge.2017.05.007

[12] C. J. H. Kramer , K. M. H. Vangangelt , G. W. van Pelt , T. J. A. Dekker , R. A. E. M. Tollenaar , W. E. Mesker , Breast Cancer Res. Treat. 2019, 173, 55. 30302588 10.1007/s10549-018-4987-4 PMC6394568

[13] P. A. Adegboyega , R. C. Mifflin , J. F. DiMari , J. I. Saada , D. W. Powell , Arch Pathol Lab Med 2002, 126, 829. 12088453 10.5858/2002-126-0829-ISOMIN

[14] D. W. Powell , P. A. Adegboyega , J. F. Di Mari , R. C. Mifflin , Am J Physiol Gastrointest Liver Physiol 2005, 289, G2. 15961883 10.1152/ajpgi.00075.2005

[15] T.‐X. Huang , X.‐Y. Tan , H.‐S. Huang , Y.‐T. Li , B.‐L. Liu , K.‐S. Liu , X. Chen , Z. Chen , X.‐Y. Guan , C. Zou , L. Fu , Gut 2022, 71, 333. 33692094 10.1136/gutjnl-2020-322924 PMC8762012

[16] J. L. Hu , W. Wang , X. L. Lan , Z. C. Zeng , Y. S. Liang , Y. R. Yan , F. Y. Song , F. F. Wang , X. H. Zhu , W. J. Liao , W. T. Liao , Y. Q. Ding , L. Liang , Mol Cancer 2019, 18, 91. 31064356 10.1186/s12943-019-1019-x PMC6503554

[17] A. M. Nicolas , M. Pesic , E. Engel , P. K. Ziegler , M. Diefenhardt , K. B. Kennel , F. Buettner , C. Conche , V. Petrocelli , E. Elwakeel , A. Weigert , A. Zinoveva , M. Fleischmann , B. Häupl , C. Karakütük , H. Bohnenberger , M. H. Mosa , L. Kaderali , J. Gaedcke , M. Ghadimi , F. Rödel , M. C. Arkan , T. Oellerich , C. Rödel , E. Fokas , F. R. Greten , Cancer Cell 2022, 40, 168. 35120600 10.1016/j.ccell.2022.01.004

[18] E. Koncina , M. Nurmik , V. I. Pozdeev , C. Gilson , M. Tsenkova , R. Begaj , S. Stang , A. Gaigneaux , C. Weindorfer , F. Rodriguez , M. Schmoetten , E. Klein , J. Karta , V. S. Atanasova , K. Grzyb , P. Ullmann , R. Halder , M. Hengstschläger , J. Graas , V. Augendre , Y. E. Karapetyan , L. Kerger , N. Zuegel , A. Skupin , S. Haan , J. Meiser , H. Dolznig , E. Letellier , Nat. Commun. 2023, 14, 4251. 37460545 10.1038/s41467-023-39953-w PMC10352362

[19] P.‐J. Sung , N. Rama , J. Imbach , S. Fiore , B. Ducarouge , D. Neves , H.‐W. Chen , D. Bernard , P.‐C. Yang , A. Bernet , S. Depil , P. Mehlen , Cancer Res. 2019, 79, 3651. 31088838 10.1158/0008-5472.CAN-18-2952

[20] K. T. Preissner , U. Reuning , Semin Thromb Hemost 2011, 37, 408. 21805447 10.1055/s-0031-1276590

[21] D. I. Leavesley , A. S. Kashyap , T. Croll , M. Sivaramakrishnan , A. Shokoohmand , B. G. Hollier , Z. Upton , IUBMB Life 2013, 65, 807. 24030926 10.1002/iub.1203

[22] K. T. Preissner , Annu Rev Cell Biol 1991, 7, 275. 1725600 10.1146/annurev.cb.07.110191.001423

[23] D. S. Harburger , D. A. Calderwood , J. Cell Sci. 2009, 122, 159. 19118207 10.1242/jcs.018093 PMC2714413

[24] V. Paradis , F. Degos , D. Dargère , N. Pham , J. Belghiti , C. Degott , J.‐L. Janeau , A. Bezeaud , D. Delforge , M. Cubizolles , I. Laurendeau , P. Bedossa , Hepatology 2005, 41, 40. 15690480 10.1002/hep.20505

[25] M. Kadowaki , T. Sangai , T. Nagashima , M. Sakakibara , H. Yoshitomi , S. Takano , K. Sogawa , H. Umemura , K. Fushimi , Y. Nakatani , F. Nomura , M. Miyazaki , J Cancer Res Clin Oncol 2011, 137, 1105. 21253761 10.1007/s00432-010-0974-9 PMC11827956

[26] M. M. Ivancic , L. W. Anson , P. J. Pickhardt , B. Megna , B. D. Pooler , L. Clipson , M. Reichelderfer , M. R. Sussman , W. F. Dove , Proc Natl Acad Sci U S A 2019, 116, 8471. 30971492 10.1073/pnas.1813212116 PMC6486772

[27] M. Nejjari , Z. Hafdi , G. Gouysse , M. Fiorentino , O. Béatrix , J. Dumortier , C. Pourreyron , C. Barozzi , A. D'Errico , W. F. Grigioni , J.‐Y. Scoazec , Hepatology 2002, 36, 418. 12143051 10.1053/jhep.2002.34611

[28] H. A. Kenny , S. Kaur , L. M. Coussens , E. Lengyel , J. Clin. Invest. 2008, 118, 1367. 18340378 10.1172/JCI33775 PMC2267016

[29] M. Aaboe , B. V. Offersen , A. Christensen , P. A. Andreasen , Biochim. Biophys. Acta 2003, 1638, 72. 12757937 10.1016/s0925-4439(03)00059-0

[30] N. A. Bhowmick , A. Chytil , D. Plieth , A. E. Gorska , N. Dumont , S. Shappell , M. K. Washington , E. G. Neilson , H. L. Moses , Science 2004, 303, 848. 14764882 10.1126/science.1090922

[31] Z. Kahounová , D. Kurfürstová , J. Bouchal , G. Kharaishvili , J. Navrátil , J. Remšík , Š. Šimečková , V. Študent , A. Kozubík , K. Souček , Cytometry A 2018, 93, 941. 28383825 10.1002/cyto.a.23101

[32] G. Friedman , O. Levi‐Galibov , E. David , C. Bornstein , A. Giladi , M. Dadiani , A. Mayo , C. Halperin , M. Pevsner‐Fischer , H. Lavon , S. Mayer , R. Nevo , Y. Stein , N. Balint‐Lahat , I. Barshack , H. R. Ali , C. Caldas , E. Nili‐Gal‐Yam , U. Alon , I. Amit , R. Scherz‐Shouval , Nat Cancer 2020, 1, 692. 35122040 10.1038/s43018-020-0082-y PMC7617059

[33] M. Iwano , D. Plieth , T. M. Danoff , C. Xue , H. Okada , E. G. Neilson , J. Clin. Invest. 2002, 110, 341. 12163453 10.1172/JCI15518 PMC151091

[34] F. Strutz , H. Okada , C. W. Lo , T. Danoff , R. L. Carone , J. E. Tomaszewski , E. G. Neilson , J. Cell Biol. 1995, 130, 393. 7615639 10.1083/jcb.130.2.393 PMC2199940

[35] A. B. Pramod , J. Foster , L. Carvelli , L. K. Henry , Mol Aspects Med 2013, 34, 197. 23506866 10.1016/j.mam.2012.07.002 PMC3602807

[36] D. Liu , D. Zhou , B. Wang , W. E. Knabe , S. O. Meroueh , ACS Chem. Biol. 2015, 10, 1521. 25671694 10.1021/cb500832q PMC4654616

[37] H. Zhang , L. Liu , J. Liu , P. Dang , S. Hu , W. Yuan , Z. Sun , Y. Liu , C. Wang , Mol Cancer 2023, 22, 58. 36941614 10.1186/s12943-023-01725-x PMC10029244

[38] R. Burgos‐Panadero , I. Noguera , A. Cañete , S. Navarro , R. Noguera , BMC Cancer 2019, 19, 479,. 31117974 10.1186/s12885-019-5693-2 PMC6532218

[39] X. Yin , Y. Zhang , S. Guo , H. Jin , W. Wang , P. Yang , Sci. Rep. 2015, 5, 12120. 26175278 10.1038/srep12120 PMC4648419

[40] L. A. Wijler , B. J. Viergever , E. Strating , S. J. van Schelven , S. Poghosyan , N. C. Frenkel , H. Te Rietmole , A. Verheem , D. A. E. Raats , I. H. M. Borel Rinkes , J. Hagendoorn , O. Kranenburg , Cancers (Basel) 2024, 16, 1073. 38473429 10.3390/cancers16051073 PMC10930793

[41] Y. Peng , L. Li , J. Shang , H. Zhu , J. Liao , X. Hong , F. F. Hou , H. Fu , Y. Liu , Theranostics 2023, 13, 3897. 37441594 10.7150/thno.85250 PMC10334827

[42] C. Jia , M. P. Keasey , H. M. Malone , C. Lovins , T. Hagg , Exp Neurol 2020, 323, 113088. 31678139 10.1016/j.expneurol.2019.113088 PMC6901344

[43] T.‐C. Ho , S.‐I. Yeh , S.‐L. Chen , Y.‐P. Tsao , Int. J. Mol. Sci. 2021, 22, 8410.34445121

[44] S. Zhang , W. Pan , H. Wang , C. Zhi , Y. Lin , P. Wu , Q. Ren , P. Wei , R. Chen , F. Li , Y. Xie , C. K. Wong , H. Tang , Z. Cai , W. Xu , H. Zeng , Mediators Inflamm 2022, 2022, 8447675,. 35462789 10.1155/2022/8447675 PMC9020974

[45] Z. Koledova , X. Zhang , C. Streuli , R. B. Clarke , O. D. Klein , Z. Werb , P. Lu , Proc Natl Acad Sci U S A 2016, 113, E5731. 27621461 10.1073/pnas.1611532113 PMC5047180

[46] E. Peuhu , R. Kaukonen , M. Lerche , M. Saari , C. Guzmán , P. Rantakari , N. De Franceschi , A. Wärri , M. Georgiadou , G. Jacquemet , E. Mattila , R. Virtakoivu , Y. Liu , Y. Attieh , K. A. Silva , T. Betz , J. P. Sundberg , M. Salmi , M.‐A. Deugnier , K. W. Eliceiri , J. Ivaska , EMBO J. 2017, 36, 165. 27974362 10.15252/embj.201694387 PMC5239997

[47] A. J. Trimboli , C. Z. Cantemir‐Stone , F. Li , J. A. Wallace , A. Merchant , N. Creasap , J. C. Thompson , E. Caserta , H. Wang , J.‐L. Chong , S. Naidu , G. Wei , S. M. Sharma , J. A. Stephens , S. A. Fernandez , M. N. Gurcan , M. B. Weinstein , S. H. Barsky , L. Yee , T. J. Rosol , P. C. Stromberg , M. L. Robinson , F. Pepin , M. Hallett , M. Park , M. C. Ostrowski , G. Leone , Nature 2009, 461, 1084. 19847259 10.1038/nature08486 PMC2767301

[48] J. Zhang , L. Chen , M. Xiao , C. Wang , Z. Qin , Am J Pathol 2011, 178, 382. 21224075 10.1016/j.ajpath.2010.11.017 PMC3070559

[49] M. W. Pickup , L. D. Hover , E. R. Polikowsky , A. Chytil , A. E. Gorska , S. V. Novitskiy , H. L. Moses , P. Owens , Mol. Oncol. 2015, 9, 179. 25205038 10.1016/j.molonc.2014.08.004 PMC4277920

[50] M. Iwano , A. Fischer , H. Okada , D. Plieth , C. Xue , T. M. Danoff , E. G. Neilson , Mol. Ther. 2001, 3, 149. 11237671 10.1006/mthe.2000.0251

[51] C. H. Österreicher , M. Penz‐Österreicher , S. I. Grivennikov , M. Guma , E. K. Koltsova , C. Datz , R. Sasik , G. Hardiman , M. Karin , D. A. Brenner , Proc Natl Acad Sci U S A 2011, 108, 308. 21173249 10.1073/pnas.1017547108 PMC3017162

[52] E. Sahai , I. Astsaturov , E. Cukierman , D. G. DeNardo , M. Egeblad , R. M. Evans , D. Fearon , F. R. Greten , S. R. Hingorani , T. Hunter , R. O. Hynes , R. K. Jain , T. Janowitz , C. Jorgensen , A. C. Kimmelman , M. G. Kolonin , R. G. Maki , R. S. Powers , E. Puré , D. C. Ramirez , R. Scherz‐Shouval , M. H. Sherman , S. Stewart , T. D. Tlsty , D. A. Tuveson , F. M. Watt , V. Weaver , A. T. Weeraratna , Z. Werb , Nat. Rev. Cancer 2020, 20, 174. 31980749 10.1038/s41568-019-0238-1 PMC7046529

[53] Q. Li , M. Liu , Y. Sun , T. Jin , P. Zhu , X. Wan , Y. Hou , G. Tu , J Exp Clin Cancer Res 2021, 40, 168. 33990217 10.1186/s13046-021-01933-7 PMC8120850

[54] Y. Feng , X. Guo , H. Tang , Ann. Transl. Med. 2021, 9, 264. 33708891 10.21037/atm-20-5984 PMC7940877

[55] L. Yuan , X. J. Wu , W. C. Li , C. Zhuo , Z. Xu , C. Tan , R. Ma , J. Wang , J. Pu , Technol Cancer Res Treat 2020, 19, 1533033820983029. 33356959 10.1177/1533033820983029 PMC7780307

[56] J. M. Loo , A. Scherl , A. Nguyen , F. Y. Man , E. Weinberg , Z. Zeng , L. Saltz , P. B. Paty , S. F. Tavazoie , Cell 2015, 160, 393. 25601461 10.1016/j.cell.2014.12.018 PMC4312495

[57] I. Kurth , N. Yamaguchi , C. Andreu‐Agullo , H. S. Tian , S. Sridhar , S. Takeda , F. C. Gonsalves , J. M. Loo , A. Barlas , K. Manova‐Todorova , R. Busby , J. C. Bendell , J. Strauss , M. Fakih , A. J. McRee , A. E. Hendifar , L. S. Rosen , A. Cercek , R. Wasserman , M. Szarek , S. L. Spector , S. Raza , M. F. Tavazoie , S. F. Tavazoie , Sci. Adv. 2021, 7, abi7511. 10.1126/sciadv.abi7511 PMC8494442 34613776

[58] E. E. Miller , A. E. Evans , M. Cohn , Proc Natl Acad Sci U S A 1993, 90, 3304. 8475072 10.1073/pnas.90.8.3304 PMC46288

[59] S. Di Biase , X. Ma , X. Wang , J. Yu , Y.‐C. Wang , D. J. Smith , Y. Zhou , Z. Li , Y. J. Kim , N. Clarke , A. To , L. Yang , J. Exp. Med. 2019, 216, 2869. 31628186 10.1084/jem.20182044 PMC6888972

[60] O. A. Maguire , S. E. Ackerman , S. K. Szwed , A. V. Maganti , F. Marchildon , X. Huang , D. J. Kramer , A. Rosas‐Villegas , R. G. Gelfer , L. E. Turner , V. Ceballos , A. Hejazi , B. Samborska , J. F. Rahbani , C. B. Dykstra , M. G. Annis , J.‐D. Luo , T. S. Carroll , C. S. Jiang , A. J. Dannenberg , P. M. Siegel , S. A. Tersey , R. G. Mirmira , L. Kazak , P. Cohen , Cell Metab. 2021, 33, 499. 33596409 10.1016/j.cmet.2021.01.018 PMC7954401

[61] L. Zhang , Z. Zhu , H. Yan , W. Wang , Z. Wu , F. Zhang , Q. Zhang , G. Shi , J. Du , H. Cai , X. Zhang , D. Hsu , P. Gao , H.‐L. Piao , G. Chen , P. Bu , Cell Metab. 2021, 33, 1111. 33811821 10.1016/j.cmet.2021.03.009

[62] V. Papalazarou , T. Zhang , N. R. Paul , A. Juin , M. Cantini , O. D. K. Maddocks , M. Salmeron‐Sanchez , L. M. Machesky , Nat Metab 2020, 2, 62. 32694686 10.1038/s42255-019-0159-z PMC7617069

[63] L. Ji , X. Zhao , B. Zhang , L. Kang , W. Song , B. Zhao , W. Xie , L. Chen , X. Hu , Immunity 2019, 51, 272. 31399282 10.1016/j.immuni.2019.06.007

[64] E. Vilar , S. B. Gruber , Nat. Rev. Clin. Oncol. 2010, 7, 153. 20142816 10.1038/nrclinonc.2009.237 PMC3427139

[65] Y. W. Choo , M. Kang , H. Y. Kim , J. Han , S. Kang , J.‐R. Lee , G.‐J. Jeong , S. P. Kwon , S. Y. Song , S. Go , M. Jung , J. Hong , B.‐S. Kim , ACS Nano 2018, 12, 8977. 30133260 10.1021/acsnano.8b02446

