# Supplementary material for: The Role of CAF‐derived Vitronectin in Promoting Colorectal Cancer Progression and Immunosuppression
Source: Adv Sci (Weinh). 2025 Jun 20;12(33):e05769. doi: 10.1002/advs.202505769 (PMC12412609; doi:10.1002/advs.202505769)
Supplement: Supplementary file 1 — Supporting Information [file ADVS-12-e05769-s003.docx]

**Supplemental material**

**Supplementary Figure Legends**


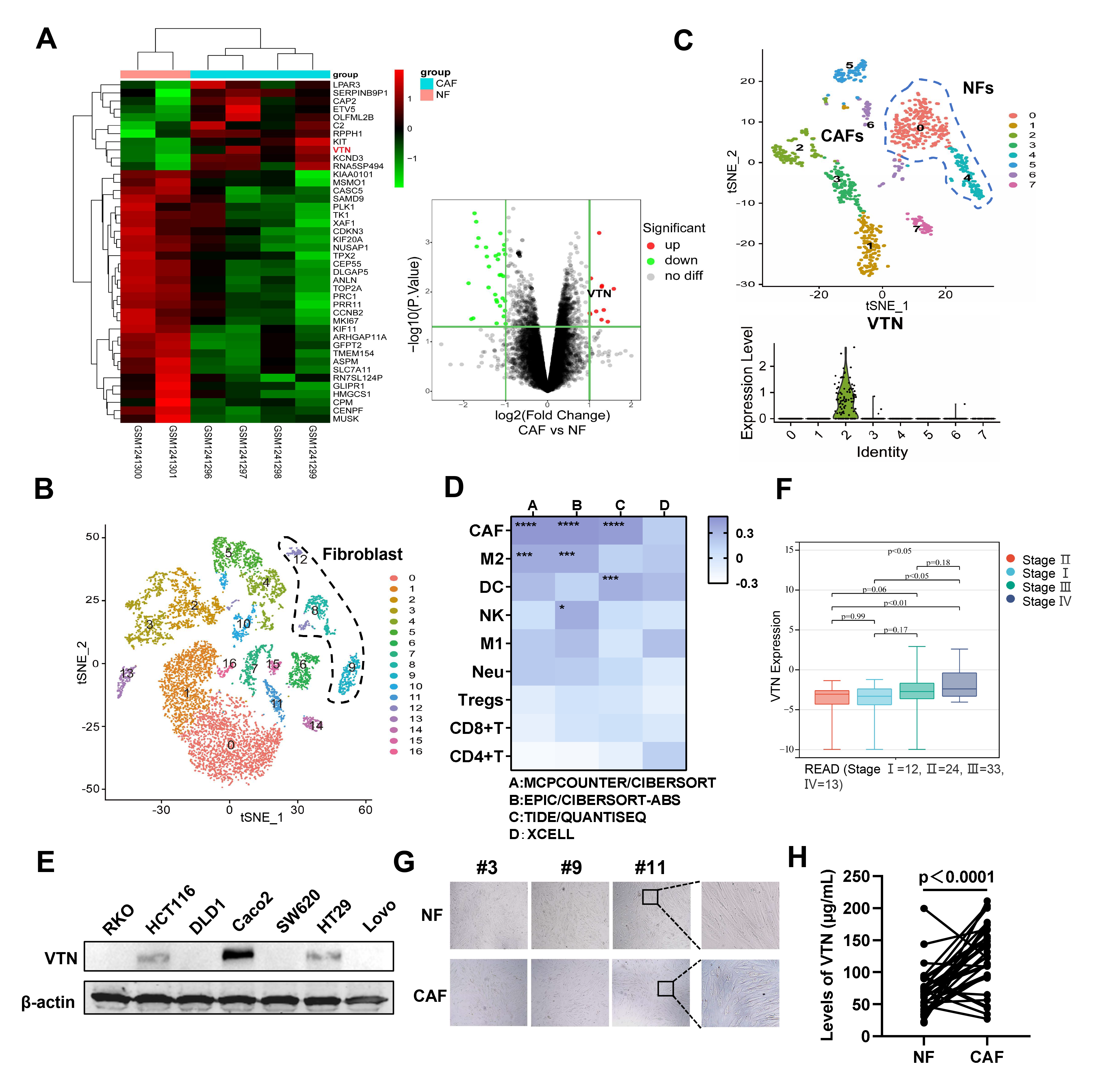
**Supplementary Figure 1. Specific Overexpression of VTN in Colorectal Cancer-Associated Fibroblasts.**

(A) Left: Heatmap illustrating the expression patterns of top differentially expressed genes between NF and CAF in dataset GSE51257. Right: Volcano plot presenting the distribution of fold change (log2FC) versus statistical significance (-log10 adjusted p-value) for all genes. The differentially expressed gene VTN is highlighted in red. (B) tSNE clustering visualization of single-cell RNA sequencing database GSE231559, comprising 6 CRC tissues and 3 adjacent normal tissues. (C) Upper: tSNE plot of total fibroblasts isolated and re-clustered from GSE231559, color-coded by clusters. Lower: Violin plot demonstrating the distribution of expression levels for VTN in fibroblast clusters. (D) Various algorithms in the TIMER2.0 database analyzed the correlation between VTN and infiltration of different cell components in CRC. *p<0.05; **p<0.01; ***p<0.001; ****p<0.0001. (E) Western blot analysis of VTN expression levels in different CRC cell lines. (F) Elevated VTN expression correlated with advanced clinical stage in CRC patients from the CRC database. (G) Microscopic observation of cell morphology of NFs and CAFs. (H) ELISA detection of VTN levels in the supernatant of 34 pairs of NFs and CAFs isolated from CRC patients. Paired two-tailed Student’s t-test was used to calculate p value.


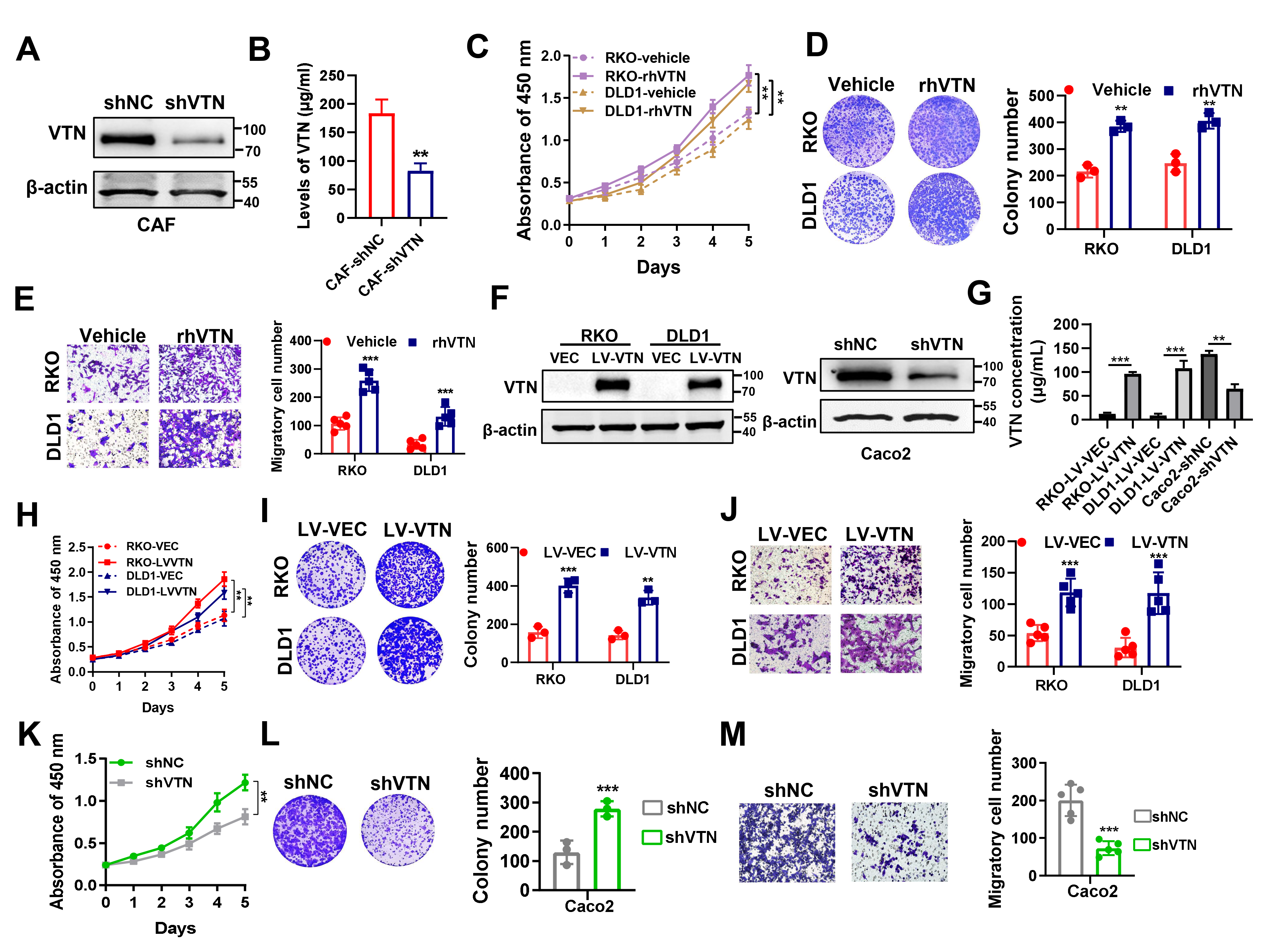
**Supplementary Figure 2.** **VTN Promotes Proliferation and Migration in Colorectal Cancer Cells.**

(A) The expression level of VTN in CAF-shNC and CAF-shVTN was detected using western blotting. (B) ELISA detection of VTN expression levels in conditioned media from CAF-shNC and CAF-shVTN. (C,D,E) Evaluation of the effects of exogenous rhVTN addition on CRC cell proliferation, colony formation, and migration using CCK-8 assay (C), colony formation assay(D), and transwell assay(E), respectively. (F) Validation of VTN overexpression and knockdown efficiency in CRC cell lines through Western blot analysis. (G) ELISA measurement of VTN expression levels in the supernatant of stable cell lines with VTN overexpression and knockdown.

(H,I,J) CCK-8 assay (H), colony formation assay(I), and transwell assay(J) were used to examine the impact of VTN overexpression on proliferation, colony formation, and migration in RKO and DLD1 cells. (K,L,M) Stable knockdown of VTN in Caco2 cells was used for CCK-8 assay (K), colony formation assay (L), and transwell assay (M), respectively.


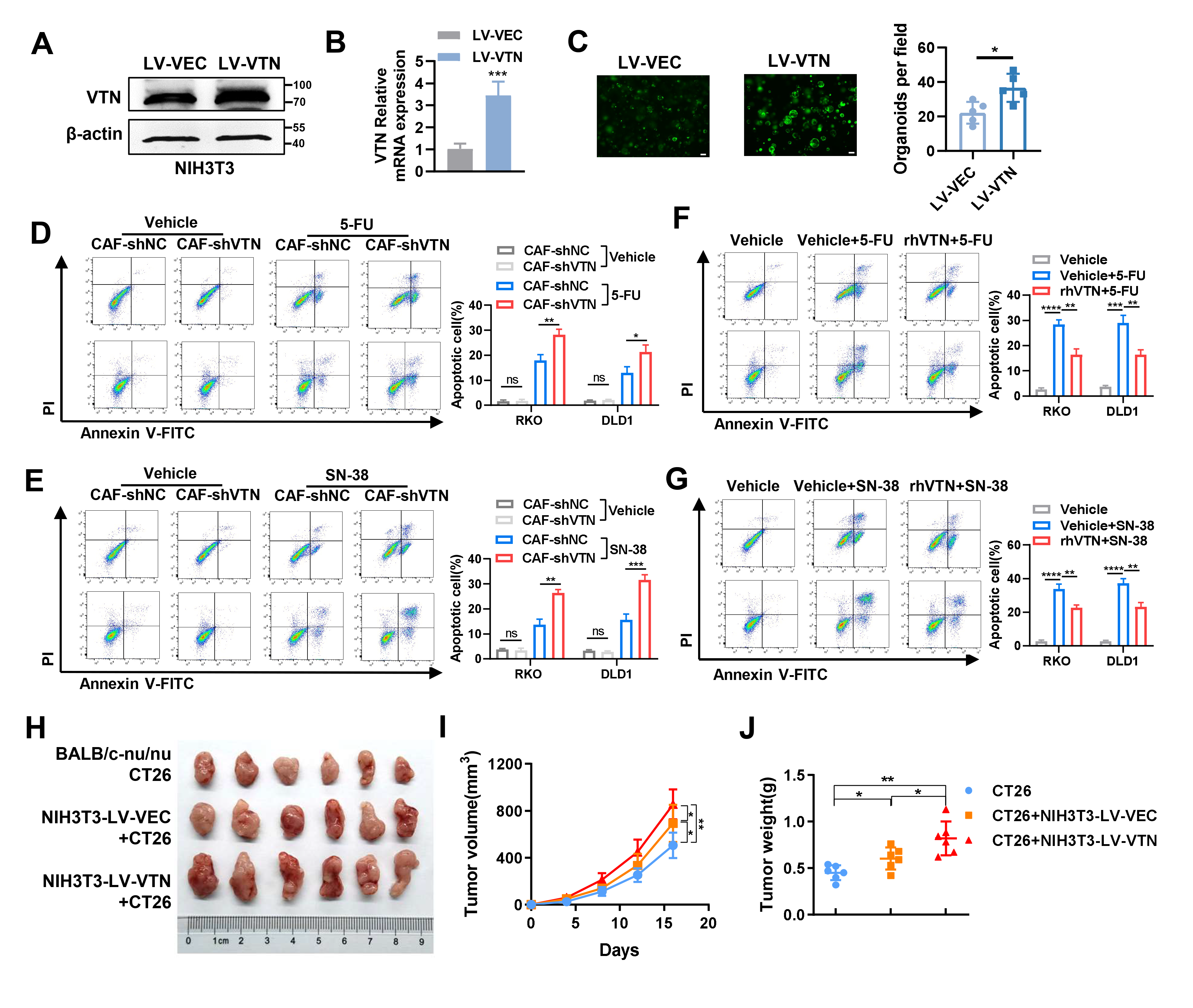
**Supplementary Figure 3. VTN Enhances Chemotherapy Resistance and Tumor Growth in Colorectal Cancer.**

(A) Western blot analysis of VTN expression in NIH3T3-LV-VEC and NIH3T3-LV-VTN cells. (B) qRT-PCR analysis of VTN mRNA levels in LV-VEC and LV-VTN organoids. (C) Representative images of organoids in LV-VEC and LV-VTN groups (left) and quantification of organoids with diameter >50 μm (right). (D,E) Flow cytometry analysis of apoptosis levels in RKO and DLD1 cells treated with 5-FU (D) or SN-38 (E) and cultured with conditioned media from CAF-shNC or CAF-shVTN. (F,G) Percentage of apoptotic cells in RKO and DLD1 cells after being cultured alone, treated with 5-FU or SN-38 alone, or treated with rhVTN (5μg/mL) and 5-FU (F) or SN-38 (G). (H,I,J) CT26 cells alone and CT26 cells together with NIH3T3-LV-VEC or NIH3T3-LV-VTN cells were diluted and subcutaneously implanted into BALB/c-nu mice (n=6/group). Photographs of subcutaneous tumors(H), tumor volume growth curves (I), and tumor weights (J). The difference between groups was determined by one-way analysis of variance or two-sided Student’s t-test. *p<0.05; **p<0.01; ***p<0.001.


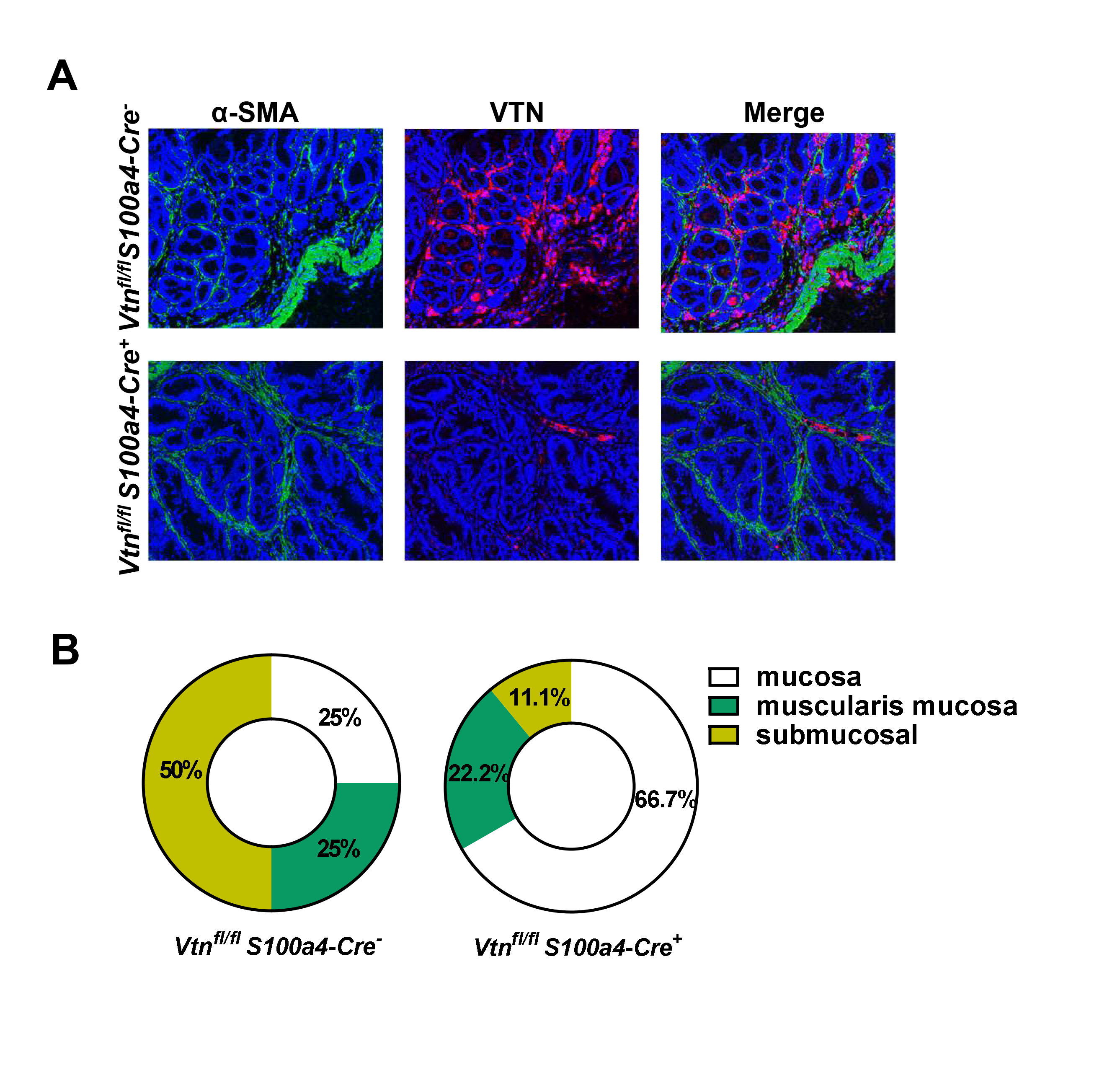


**Supplementary Figure 4. Immunofluorescence Staining and Tumor Invasion in Intestinal Tumors of *VTN^cKO^* Mice.**

(A) Immunofluorescence staining for α-SMA and VTN in intestinal tumor tissues from *Vtn^fl/fl^ S100a4-Cre^-^* and *Vtn^fl/fl^ S100a4-Cre^+^*mice. scale bar, 50 µm. (B) Statistical illustration of the degree of tumor invasion.


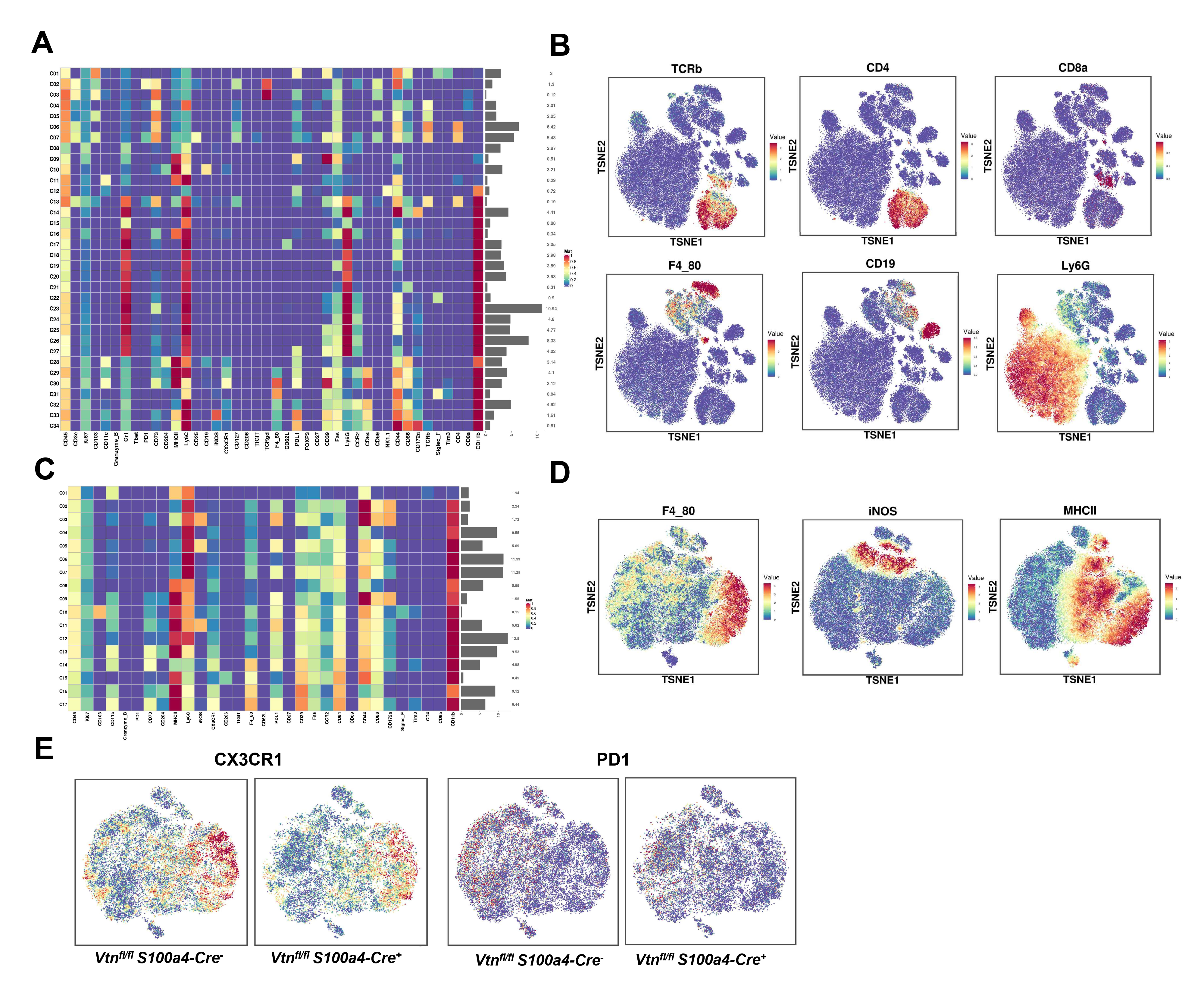
**Supplementary Figure 5. The Results of CyTOF Analysis from *Vtn^fl/fl^ S100a4-Cre^-^* and *Vtn^fl/fl^ S100a4-Cre^+^* Mice.**

(A) Heatmap of normalized expression for 42 markers expressed across each cell cluster identified in the CyTOF analysis from 6 AOM/DSS-induced CRC tissues. (B) The part of classical cell surface markers used for the annotation of various immune cells is presented. (C) Heatmap displaying the expression levels of immunomarkers within macrophage cell clusters following the extraction and re-clustering of TAMs. (D) Visualization of the t-SNE map showing part of the macrophage classical cell surface markers. (E) t-SNE plot of all TAMs colored by CX3CR1 and PD1 expression levels in *Vtn^fl/fl^ S100a4-Cre^-^* and *Vtn^fl/fl^ S100a4-Cre^+^* mice.


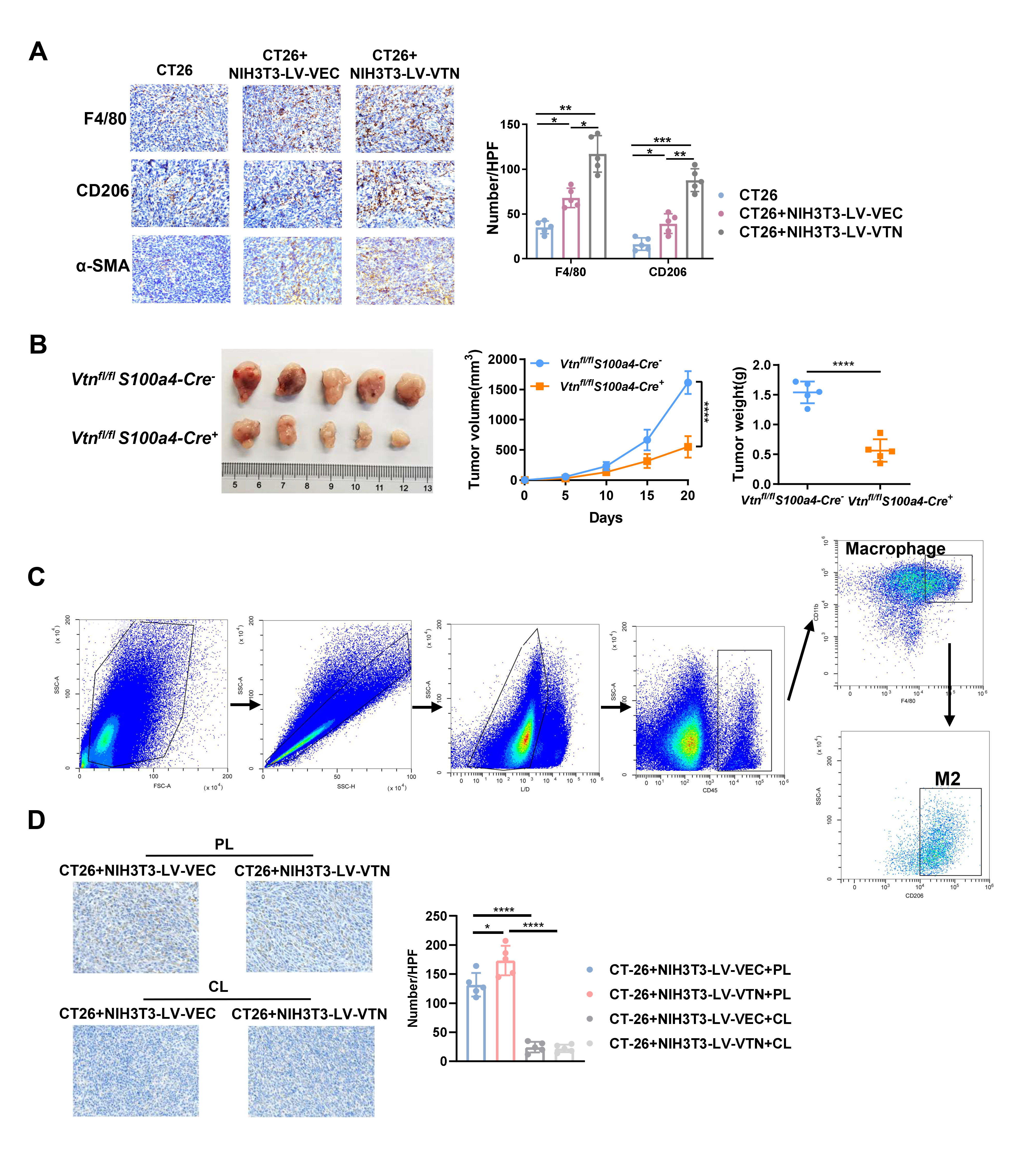
**Supplementary Figure 6. The Effect of VTN on Tumor Macrophage Polarization in Mice.**

(A) Immunohistochemistry staining of F4/80, CD206, and α-SMA in tumor tissues derived from the subcutaneous co-injection of CT26 cells with NIH3T3 (LV-VEC or LV-VTN) cells. Scale bar, 50 µm. (B) Representative images of tumors in mice from *Vtn^fl/fl^ S100a4-Cre^-^* and *Vtn^fl/fl^ S100a4-Cre^+^* groups (left), tumor volume growth curves (middle), and tumor weights (right). (C) Gating strategy for stepwise flow cytometry sorting of target cell populations. (D) Representative images and quantitative analysis of F4/80 staining in tumor samples derived from the subcutaneous co-injection of CT26 cells with NIH3T3 cells (LV-VEC or LV-VTN) treated with PBS liposome (PL) or clodronate liposome (CL).


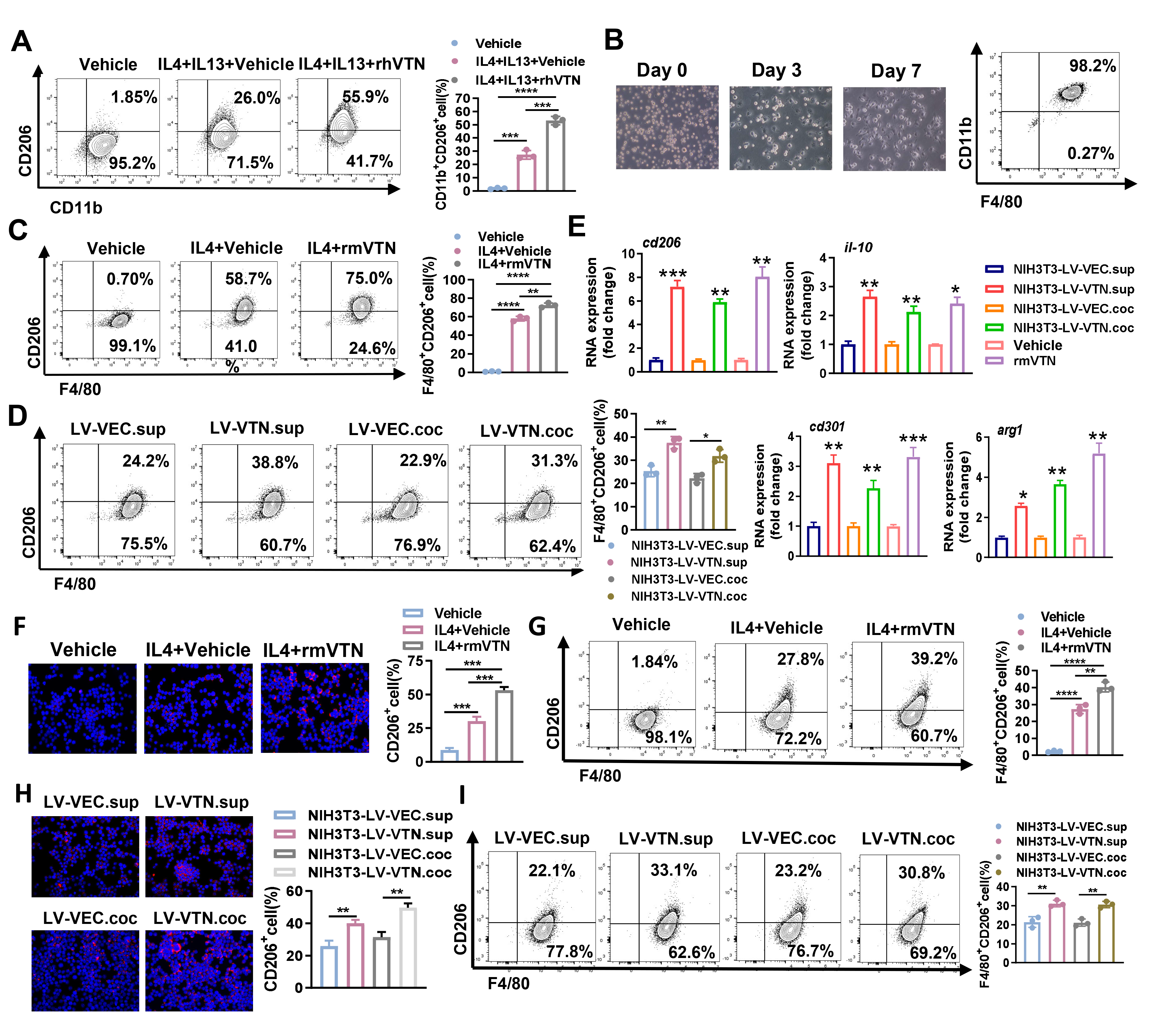
**Supplementary Figure 7. Analysis of the Impact of VTN on Macrophage Polarization in Vitro.**

(A) Flow cytometry analysis showing the proportion of CD206^+^CD11b^+^ THP-1-derived TAMs following treatment with vehicle or rhVTN (5μg/mL). (B) Left: Morphological changes of BMDMs at different induction time points were observed microscopically. Right: The percentage of CD11b^+^F4/80^+^ BMDMs induced by macrophage colony-stimulating factor (M-CSF) for 7 days was determined by flow cytometry. (C) Flow cytometry was used to quantify the percentage of F4/80^+^CD206^+^BMDMs treated with vehicle or rmVTN (5μg/mL). (D) The percentage of F4/80^+^CD206^+^BMDMs following supernatant culture or co-culture with NIH3T3-LV-VEC and NIH3T3-LV-VTN cells. (E) The mRNA expression levels of M2-like TAMs markers cd301, cd206, arg1, and il10 were examined by qRT-PCR in RAW264.7-derived TAMs. (F,G) Cellular immunofluorescence(F) and Flow cytometry (G) analyses were employed to evaluate the proportion of M2-like TAMs in RAW264.7-derived TAMs treated with vehicle or rmVTN (5μg/mL). (H) Cellular immunofluorescence staining demonstrated the proportion of CD206-positive cells in RAW264.7 cells treated with supernatant culture or co-culture with NIH3T3-LV-VEC and NIH3T3-LV-VTN cells. (I) Flow cytometry analysis of the percentage of F4/80^+^CD206^+^ RAW264.7-derived TAMs following treatment with supernatant culture or co-culture with NIH3T3-LV-VEC/VTN cells.

The statistical significance of the observed differences between groups was determined by one-way analysis of variance or two-sided Student’s t-test. *p<0.05; **p<0.01; ***p<0.001.


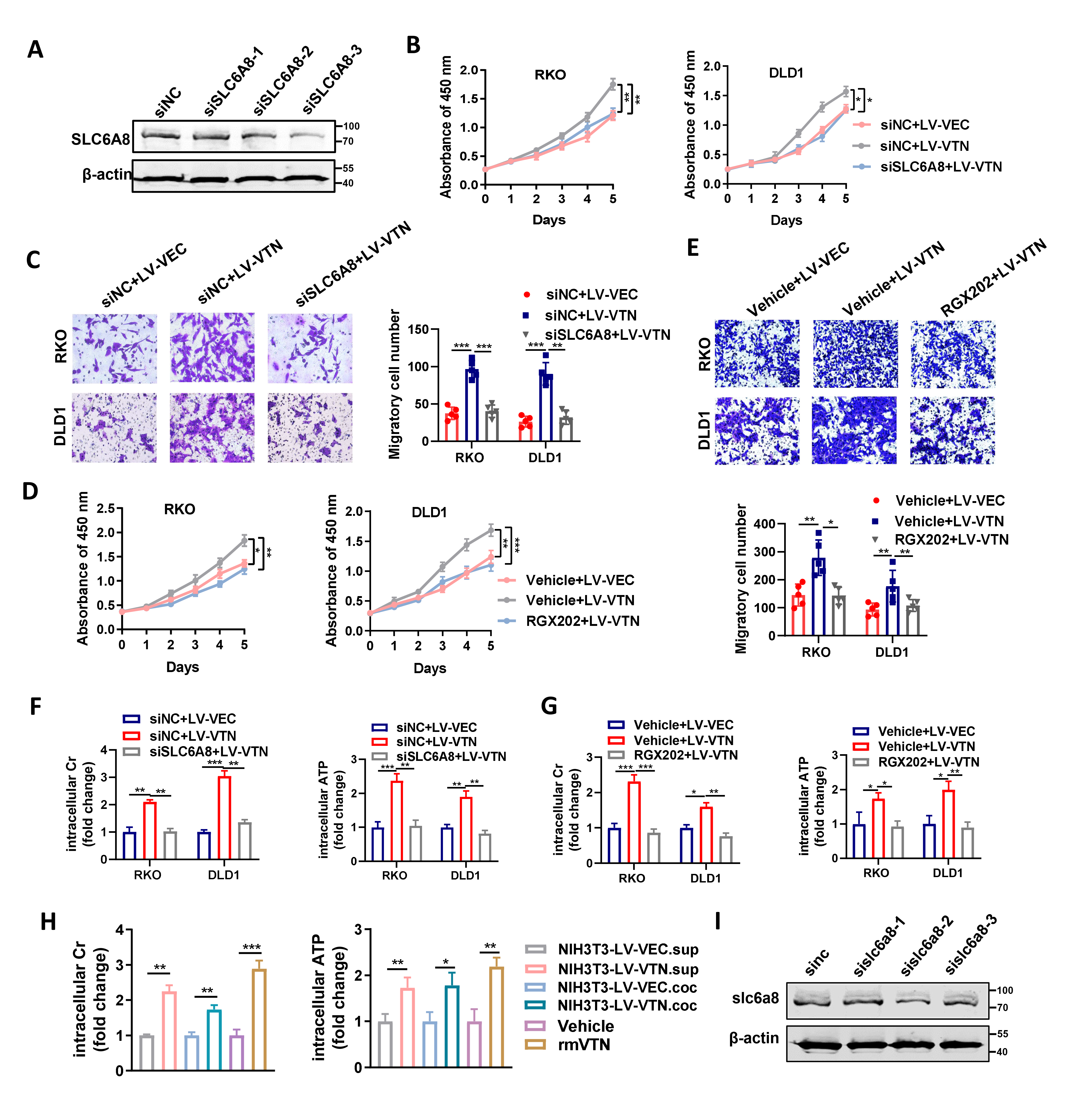
**Supplementary Figure 8.** **Effect of SLC6A8 Downregulation on VTN Pro-cancer Activity.**

(A) The efficacy of siRNAs targeting SLC6A8 was confirmed by western blot analysis. (B,C) Stable overexpression of VTN in RKO and DLD1 cells transfected with SLC6A8 siRNAs or mock controls were used for CCK-8 assays (B) and transwell migration assays (C). (D,E) CCK-8 assays(D) and transwell migration assays (E)were performed in RKO and DLD1 cells with stable overexpression of VTN treated with vehicle or SLC6A8 inhibitor RGX202. (F,G) The relative intracellular concentrations of creatine and ATP of RKO and DLD1 cells with VTN overexpression were treated with SLC6A8 knockdown (F) or RGX202(G). (H) Relative intracellular levels of creatine and ATP were assessed in RAW264.7 macrophages following supernatant culture or co-culture with NIH3T3-LV-VEC and NIH3T3-LV-VTN cells, supplemented with exogenous rmVTN. (I) Western blot analyses of SLC6A8 expression in RAW264.7 cells transfected with siRNAs targeting slc6a8. Group differences were analyzed for statistical significance using a one-way ANOVA or a two-sided Student's t-test. *p<0.05; **p<0.01; ***p<0.001.


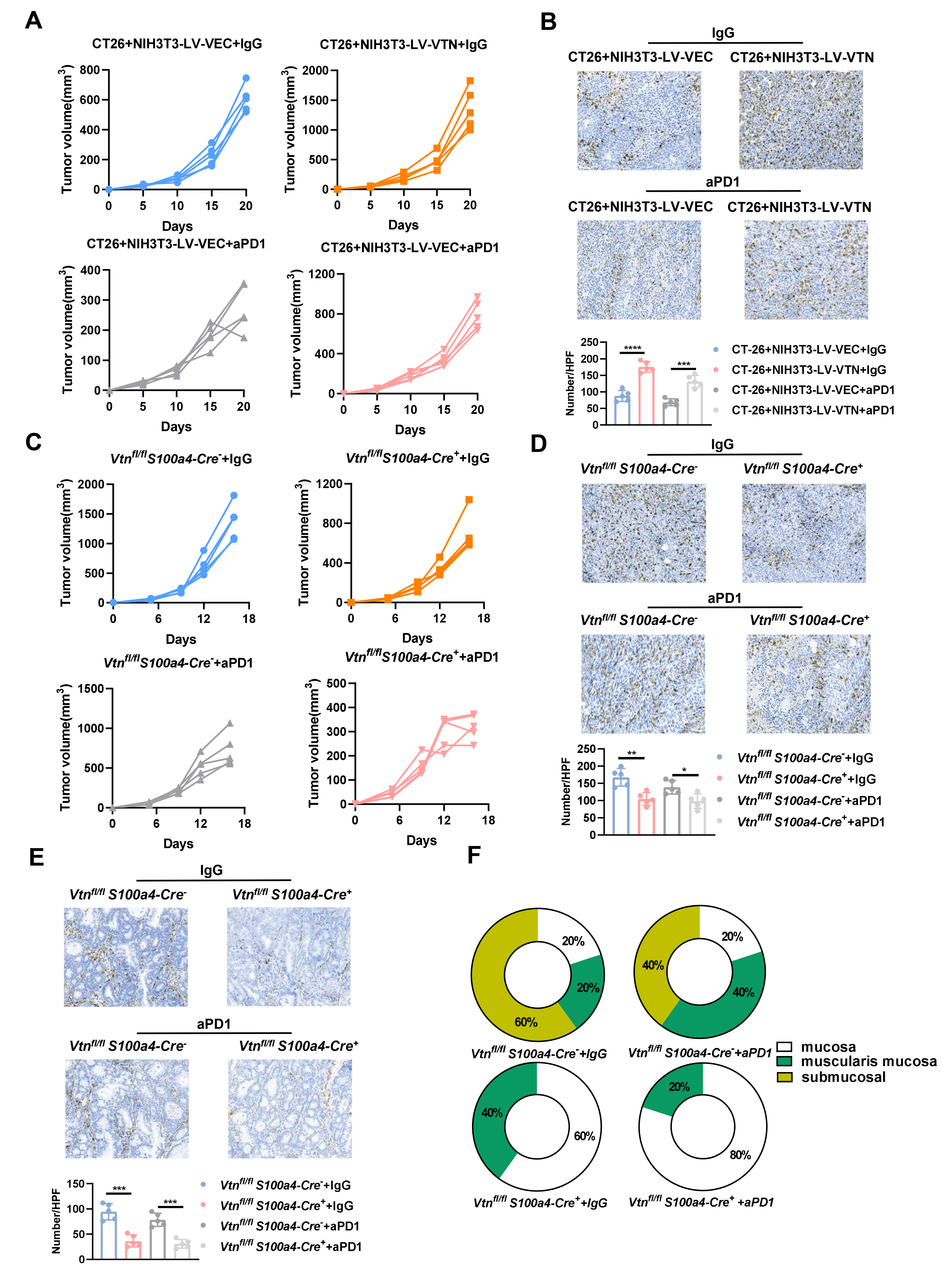
**Supplementary Figure 9.** **Immunohistochemical Analysis and Tumor Development and Invasion in Different Mouse Tumor Models.**

(A,B) Tumor growth (A) and representative IHC staining images and statistical analysis of CD206 expression (B) in subcutaneous tumor-bearing mice co-injected with CT26 and NIH3T3 (LV-VEC/LV-VTN) cells and treated with either isotype control (IgG) or anti-PD-1 monoclonal antibody (aPD1). (C,D) Tumor growth (C) and representative IHC staining and statistical analysis of CD206 expression (D) in subcutaneous tumors from *Vtn^fl/fl^ S100a4-Cre^-^* and *Vtn^fl/fl^ S100a4-Cre^+^* mice treated with isotype control or anti-PD-1 mAb. (E) CD206 expression in AOM/DSS-induced tumors was assessed by representative IHC staining and statistical analysis in *Vtn^fl/fl^ S100a4-Cre^-^* and *Vtn^fl/fl^ S100a4-Cre^+^* mice treated with isotype control or anti-PD-1 mAb. (F) Statistical illustration of the degree of tumor invasion.

**Supplementary Table**

Supplementary Table 1. The antibodies used in this study.

| Antibodies | Source | Identifier |
| --- | --- | --- |
| VTN | Abclonal | A1667 |
| VTN | Proteintech | 66398-1-Ig |
| FAP | Cell Signaling Technologies | #66562 |
| Vimentin | Cell Signaling Technologies | #5741 |
| α-SMA | Abclonal | A17910 |
| S100A4 | Proteintech | 16105-1-AP |
| F4/80 | Abclonal | A18637 |
| CD206 | Abclonal | A8301 |
| CD3 | Abcam | ab237707 |
| CD31 | Abcam | ab76533 |
| MPO | Proteintech | 22225-1-AP |
| CD11c | Proteintech | 17342-1-AP |
| CD68 | Abcam | Ab303565 |
| SLC6A8 | Proteintech | 20299-1-AP |
| FAK | Proteintech | 12636-1-AP |
| p-FAK | Cell Signaling Technologies | #3283 |
| β-actin | Proteintech | 66009-1-Ig |
| FITC anti-human CD11b | Biolegend | 301330 |
| PE anti-human CD206 | BD Pharmingen | 555954 |
| APC anti-mouse F4/80 | Biolegend | 123115 |
| PE anti-mouse CD206 | Biolegend | 141705 |
| APC-Cy7 anti-mouse CD45 | BD Pharmingen | 557659 |
| FITC anti-CD11b | BD Pharmingen | 557396 |
| APC anti-mouse F4/80 | BD Pharmingen | 566787 |
| Fixable Viability Stain 510 | BD Pharmingen | 564406 |
| Anti-mouse CD16/32 | Biolegend | 156603 |

Supplementary Table 2. List of Primers sequence used in the study for qRT-PCR.

| Gene | Species | Forward Primer | Reverse Primer |  |
| --- | --- | --- | --- | --- |
| VTN | human | TACCCCAAGCTCATCCGAGA | AGGACACCATCCTCAAAGCG |  |
| SLC6A8 | human | GATCACCATGGAGGCGTAG | TGATCGCCCTGGTTGGA |  |
| BEST1 | human | AGCCTGAACAAAGAGGAGATGG | CTGATACAGTGGGGCAGACTTG |  |
| IL17D | human | CAGGTACCTGCCTGAAGCCTACTGC | CAGCAGGAGCTTGGCGCCCTGTTTG |  |
| EGR1 | human | CTTCAACCCTCAGGCGGACA | GGAAAAGCGGCCAGTATAGGT |  |
| IL7R | human | TCCAACCGGCAGCAATGTAT | GATCCATCTCCCCTGAGCTA |  |
| PIK3AP1 | human | CCCAGAGGATGCGACATC | GACAGCAGCACCACGACA |  |
| CAVIN1 | human | GGCAGATCAAGAAGCTGGAGGT | CAGCGATTTGCTGATGCTCAGTT |  |
| NDGR1 | human | CGAGGACATGCAGGAGATCA | TCAGCCCAAACTGTTGAAGGA |  |
| IL36RN | human | TGGAAGCCAGTGCCTGTCATGT | AGCCGACTCGAAGCTGGAGGT |  |
| EGR1 | human | CTTCAACCCTCAGGCGGACA | GGAAAAGCGGCCAGTATAGGT |  |
| JUN | human | GGCTGGTGTTTCGGGAGTGT | CGCCGCCTTCTGGTCTTTAC |  |
| CFTR | human | TGCCCTTCGGCGATGTTT | GCGATAGAGCGTTCCTCCTTG |  |
| IL17C | human | TTGGAGGCAGACACCCACC | GATAGCGGTCCTCATCCGTG |  |
| IRF8 | human | AGTGGCTGATCGAGCAGATT | AAAGCACAGCGTAACCTCGT |  |
| IL33 | human | CTGCCTGTCAACAGCAGTCT | CTGGTCTGGCAGTGGTTTTT |  |
| β-actin | human | CCTGGCACCCAGCACAATG | GGGCCGGACTCGTCATACT |  |
| CD163 | human | TTTGTCAACTTGAGTCCCTTCAC | TCCCGCTACACTTGTTTTCAC |  |
| TGF-β1 | human | GGCCAGATCCTGTCCAAGC | GTGGGTTTCCACCATTAGCAC |  |
| ARG1 | human | TGCCCTTTGCTGACATCCCTAAT | CTTCTTGACTTCTGCCACCTT |  |
| IL10 | human | GAGAACCAAGACCCAGACATCA | AAGGCATTCTTCACCTGCTCCAC |  |
| cd301 | mouse | CAGCTTGCTCCCCTCTACCT | TCCAACGACCATCGTAAGAAAAG |  |
| cd206 | mouse | CTCTGTTCAGCTATTGGACGC | CGGAATTTCTGGGATTCAGCTTC |  |
| arg1 | mouse | CCAGAAGAATGGAAGAGTCAG | CAGATATGCAGGGAGTCACC |  |
| Il-10 | mouse | GCTCTTACTGACTGGCATGAG | CGCAGCTCTAGGAGCATGTG |  |
| gapdh | mouse | AGGTCGGTGTGAACGGATTTG | TGTAGACCATGTAGTTGAGGTCA |  |
|  | |  |  |  |

Supplementary Table 3. Antibody and metal isotopes needed for CyTOF.

| List | Label | Marker |
| --- | --- | --- |
| 1 | 89Y | CD45 |
| 2 | 115ln | CD3e |
| 3 | 139La | Ki67 |
| 4 | 141Pr | CD103 |
| 5 | 142Nd | CD11c |
| 6 | 143Nd | Granzyme_B |
| 7 | 144Nd | Gr1 |
| 8 | 145Nd | Tbet |
| 9 | 146Nd | PD1 |
| 10 | 147Sm | CD73 |
| 11 | 148Nd | CD204 |
| 12 | 149Sm | MHCII |
| 13 | 150Nd | Ly6C |
| 14 | 151Eu | CD25 |
| 15 | 152Sm | CD19 |
| 16 | 153Eu | iNOS |
| 17 | 154Sm | CX3CR1 |
| 18 | 155Gd | CD127 |
| 19 | 156Gd | CD206 |
| 20 | 157Gd | TIGIT |
| 21 | 158Gd | TCRgd |
| 22 | 159Tb | F4_80 |
| 23 | 160Gd | CD62L |
| 24 | 161Dy | PDL1 |
| 25 | 162Dy | FOXP3 |
| 26 | 163Dy | CD27 |
| 27 | 164Dy | CD39 |
| 28 | 165Ho | Fas |
| 29 | 166Er | Ly6G |
| 30 | 167Er | CCR2 |
| 31 | 168Er | CD64 |
| 32 | 169Tm | CD69 |
| 33 | 170Er | NK1.1 |
| 34 | 171Yb | CD44 |
| 35 | 172Yb | CD86 |
| 36 | 173Yb | CD172a |
| 37 | 174Yb | TCRb |
| 38 | 175Lu | Siglec_F |
| 39 | 176Yb | Tim3 |
| 40 | 197Au | CD4 |
| 41 | 198Pt | CD8a |
| 42 | 209Bi | CD11b |

Supplementary Table 4. List of siRNA sequences used in the study.

| Gene Target | Species | sense（5'-3'） |
| --- | --- | --- |
| siNC | human | UUCUCCGAACGUGUCACGUTT |
| si-VTN-1 | human | GGAAGACCUACCUCUUCAATT |
| si-VTN-2 | human | GCAGACACCUGUUCUGAAATT |
| si-SLC6A8-1 | human | GCUGGUCUACAACAACACCUA |
| si-SLC6A8-2 | human | CGCCACCAGUAUCAAUUGUTT |
| si-SLC6A8-3 | human | CACCAUGAGUGCUCACUAATT |
| si-slc6a8-1 | mouse | UACAACAACACCUACGUGUA |
| si-slc6a8-2 | mouse | CCACAACUAAGCUGGUAUUU |
| si-slc6a8-3 | mouse | GCAGCUACAAUCGCUUCAATT |

Supplementary Table 5. The reagents used in this study.

| Reagents | Source | Identifier |
| --- | --- | --- |
| Collagenase Type 1 | Thermofisher | 17100017 |
| Vitronectin Protein, Human | MedChemExpress | HY-P70485 |
| Vitronectin Protein, Mouse | MedChemExpress | HY-P73484 |
| RGD Peptides | Selleck | S8008 |
| SB273005 | Selleck | S7540 |
| IPR-803 | MedChemExpress | HY-111192 |
| PF-562271 | Selleck | S2890 |
| RGX-202 | Selleck | E1223 |
| CRC Organoid Complete Medium | K2103-CR | bioGenous |
| Organoid Culture ECM | M315066 | bioGenous |
| Organoid Dissociation Solution | E238001 | bioGenous |
| M-CSF Protein, Mouse | MedChemExpress | HY-P70263A |
| IL-4 Protein, Mouse | MedChemExpress | HY-P70644 |
| IL-4 Protein, Human | MedChemExpress | HY-P70445 |
| IL-13Protein, Human | MedChemExpress | HY-P70568 |
| Phorbol Myristate Acetate | MedChemExpress | HY-18739 |
| AOM | Sigma-Aldrich | A5486 |
| DSS | MP Biomedicals | 160110 |
| Human Vitronectin (VTN) ELISA Kit | Thermofisher | EHVTN |
| EnzyChromTM Creatine Assay Kit | BioAssay Systems | ECRT-100 |
| EnzyLightTM ATP Assay Kit | BioAssay Systems | EATP-100 |
| Clodronate Liposomes | Yeasen | 40337ES10 |
| Control Liposomes | Yeasen | 40338ES10 |
