# Supplementary figures and images for: The Role of CAF‐derived Vitronectin in Promoting Colorectal Cancer Progression and Immunosuppression
Source: Adv Sci (Weinh). 2025 Jun 20;12(33):e05769. doi: 10.1002/advs.202505769 (PMC12412609; doi:10.1002/advs.202505769)

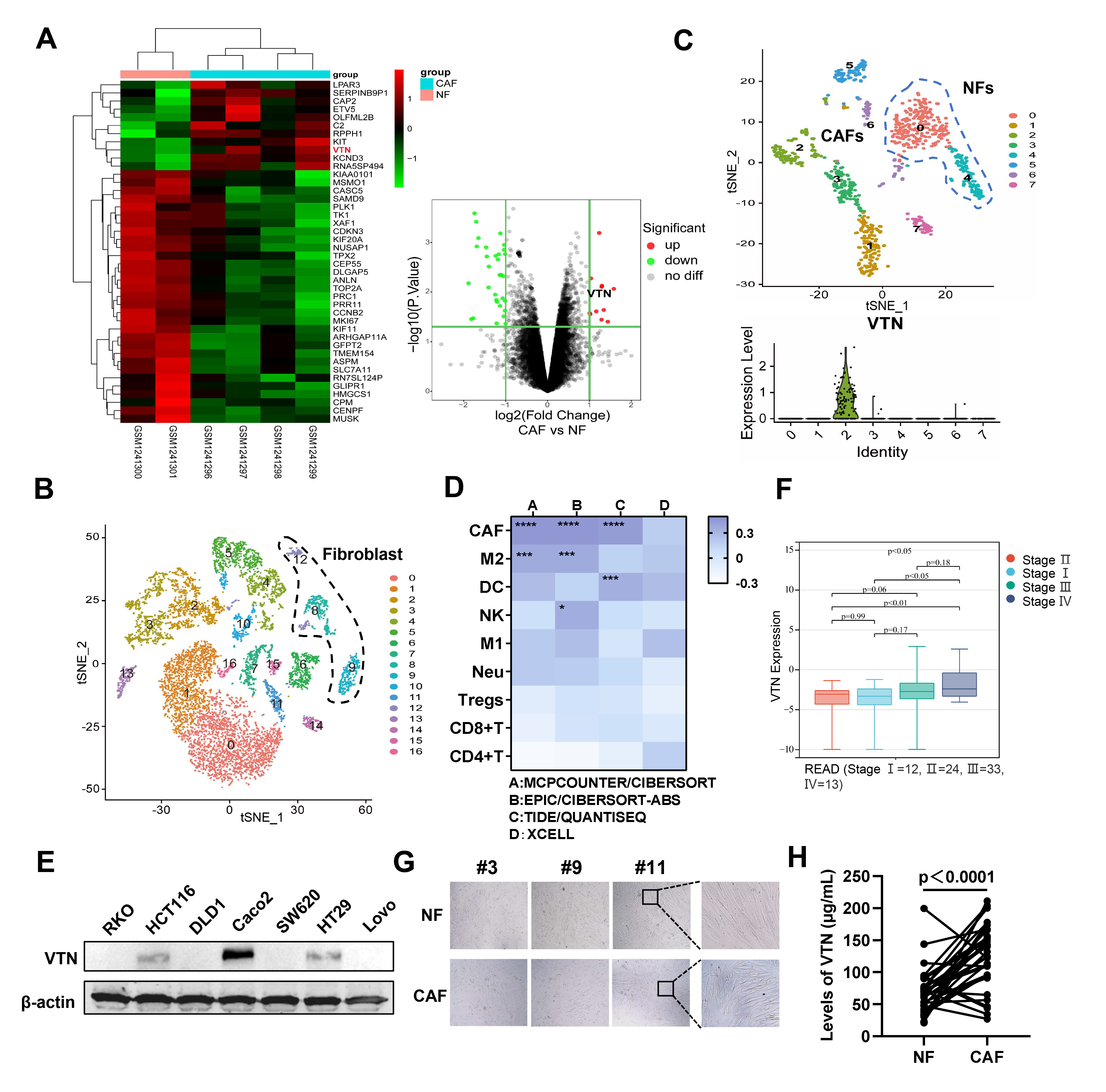

Supplement: Supplementary file 2 — Supporting Information [file ADVS-12-e05769-s006.tif]

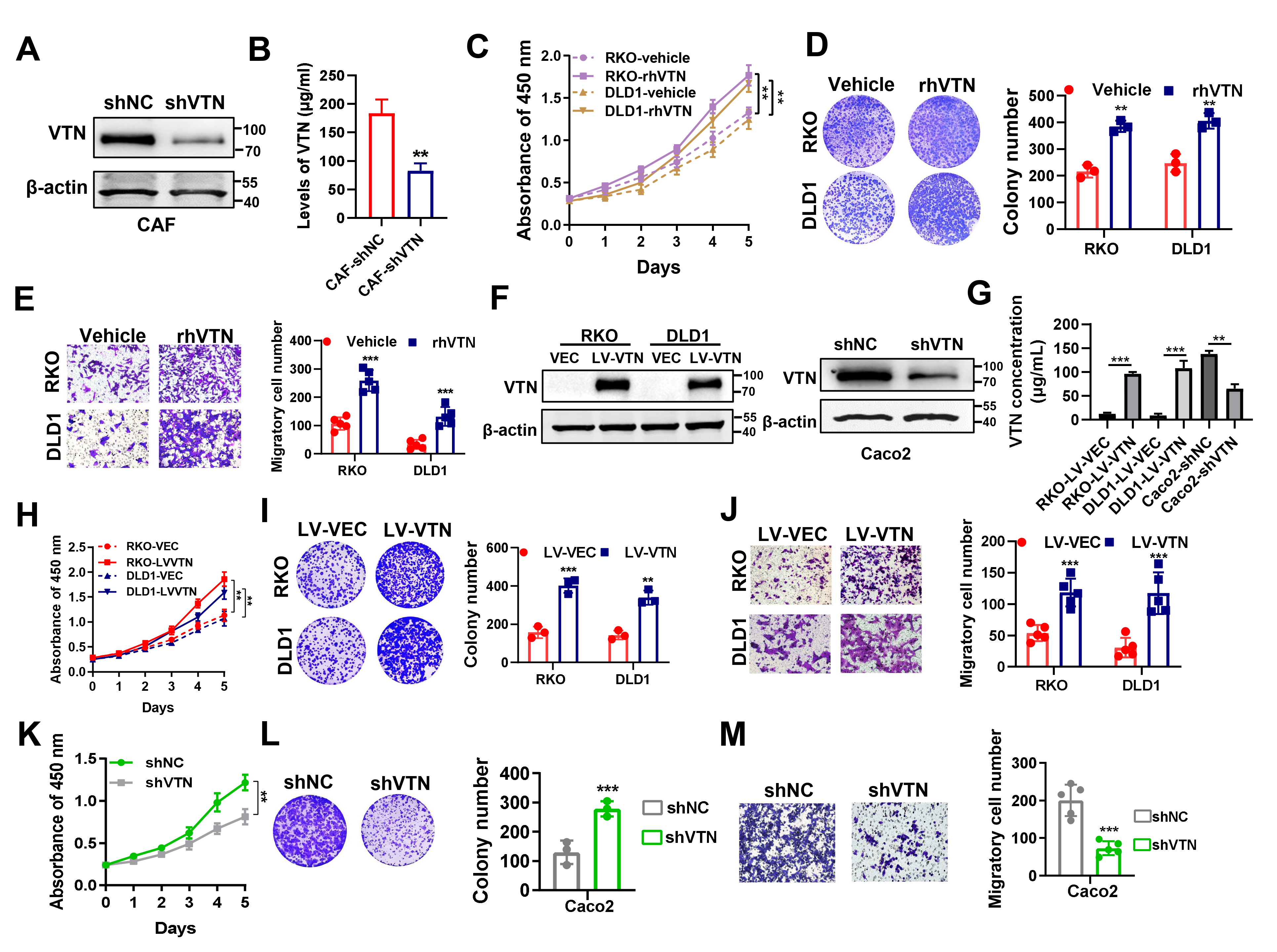

Supplement: Supplementary file 3 — Supporting Information [file ADVS-12-e05769-s005.tif]

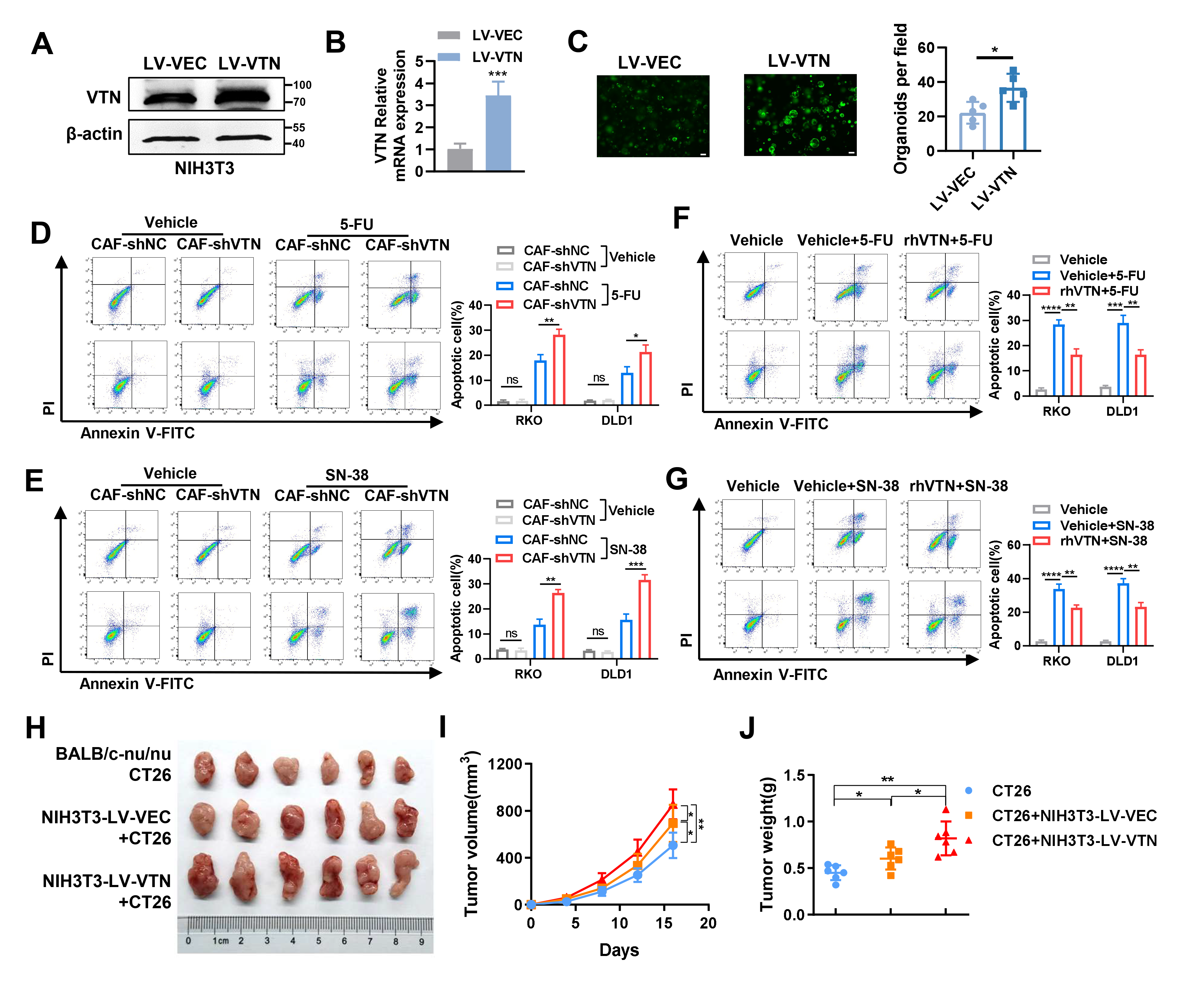

Supplement: Supplementary file 4 — Supporting Information [file ADVS-12-e05769-s001.tif]

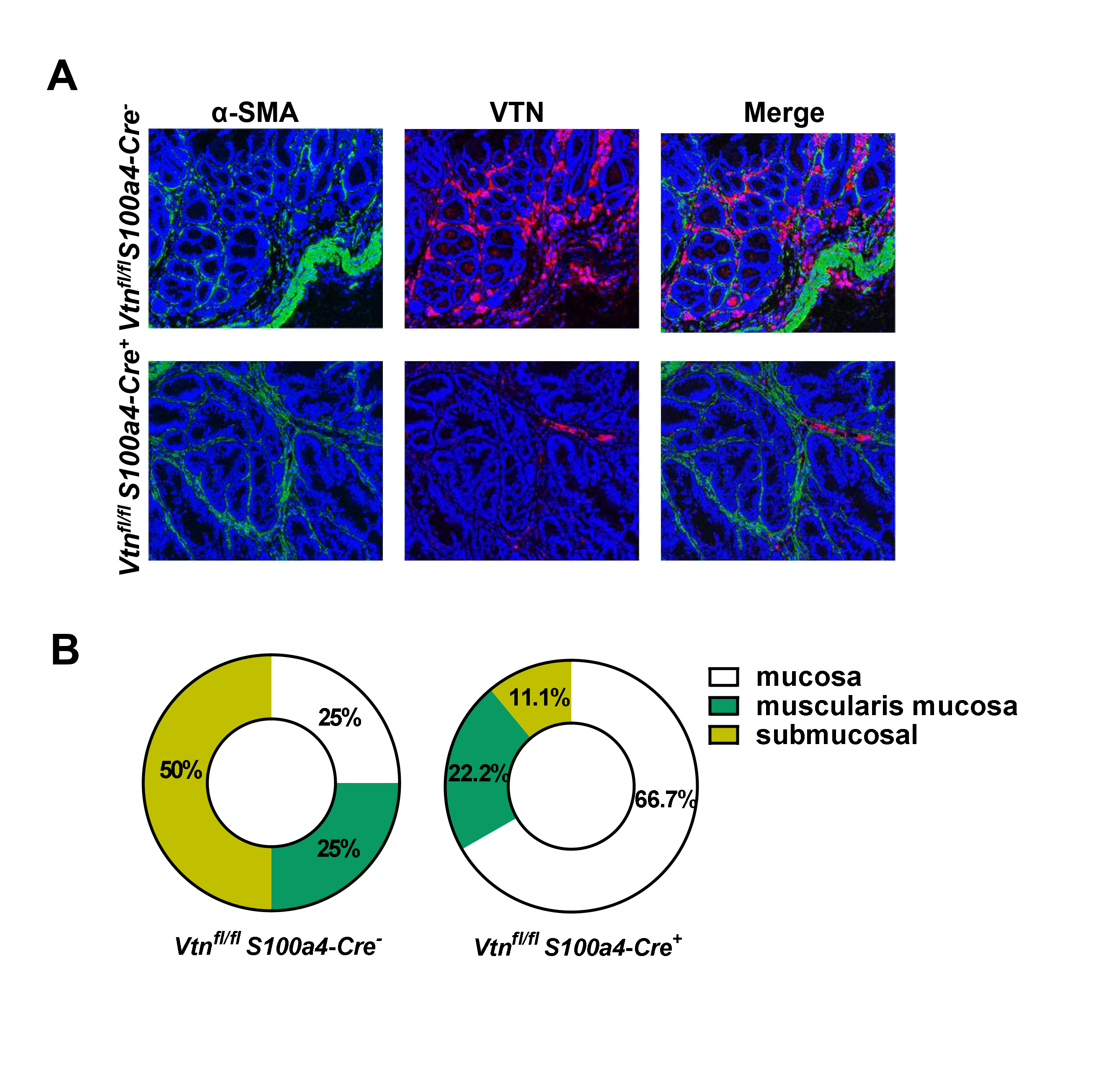

Supplement: Supplementary file 5 — Supporting Information [file ADVS-12-e05769-s002.tif]

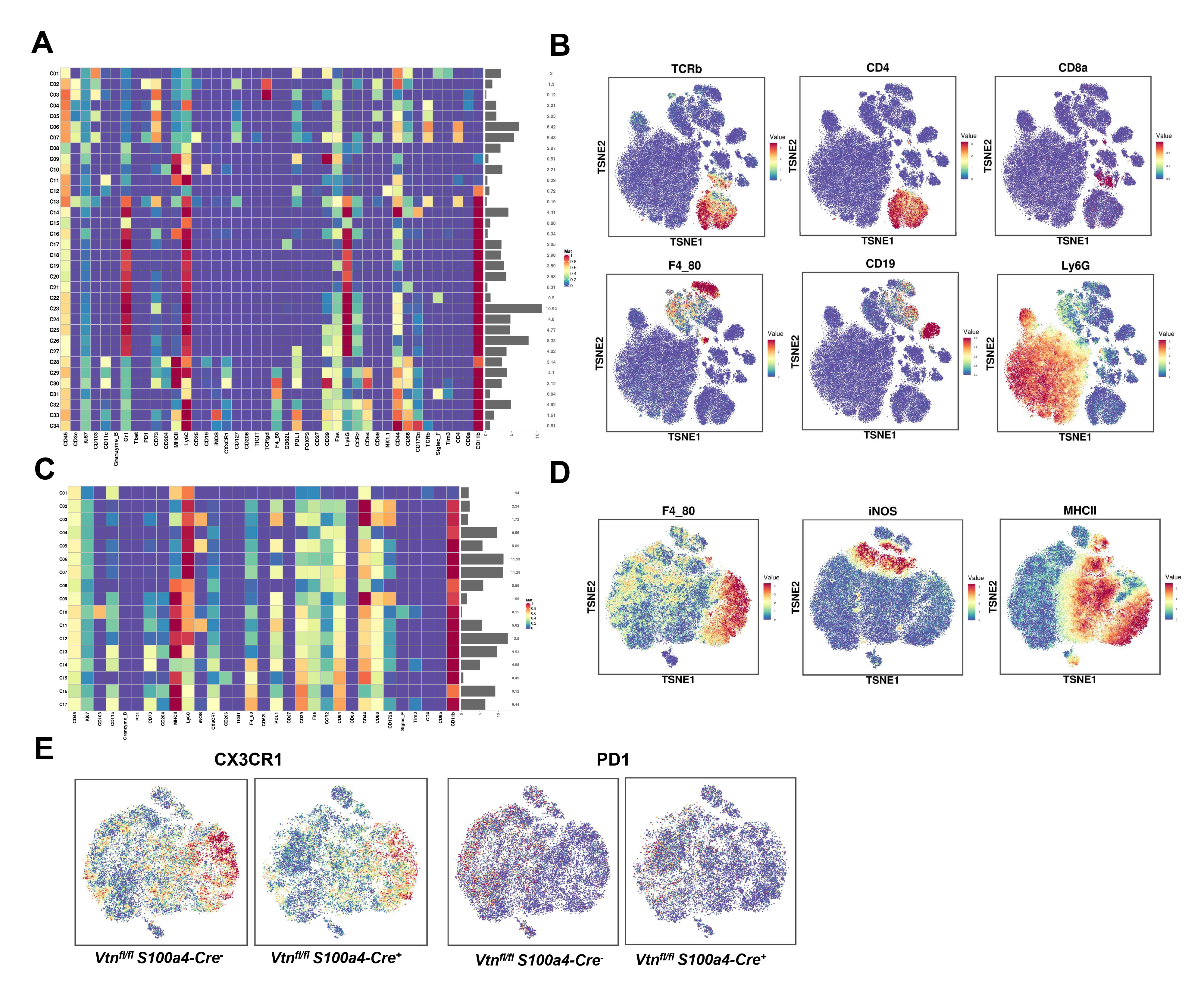

Supplement: Supplementary file 6 — Supporting Information [file ADVS-12-e05769-s009.tif]

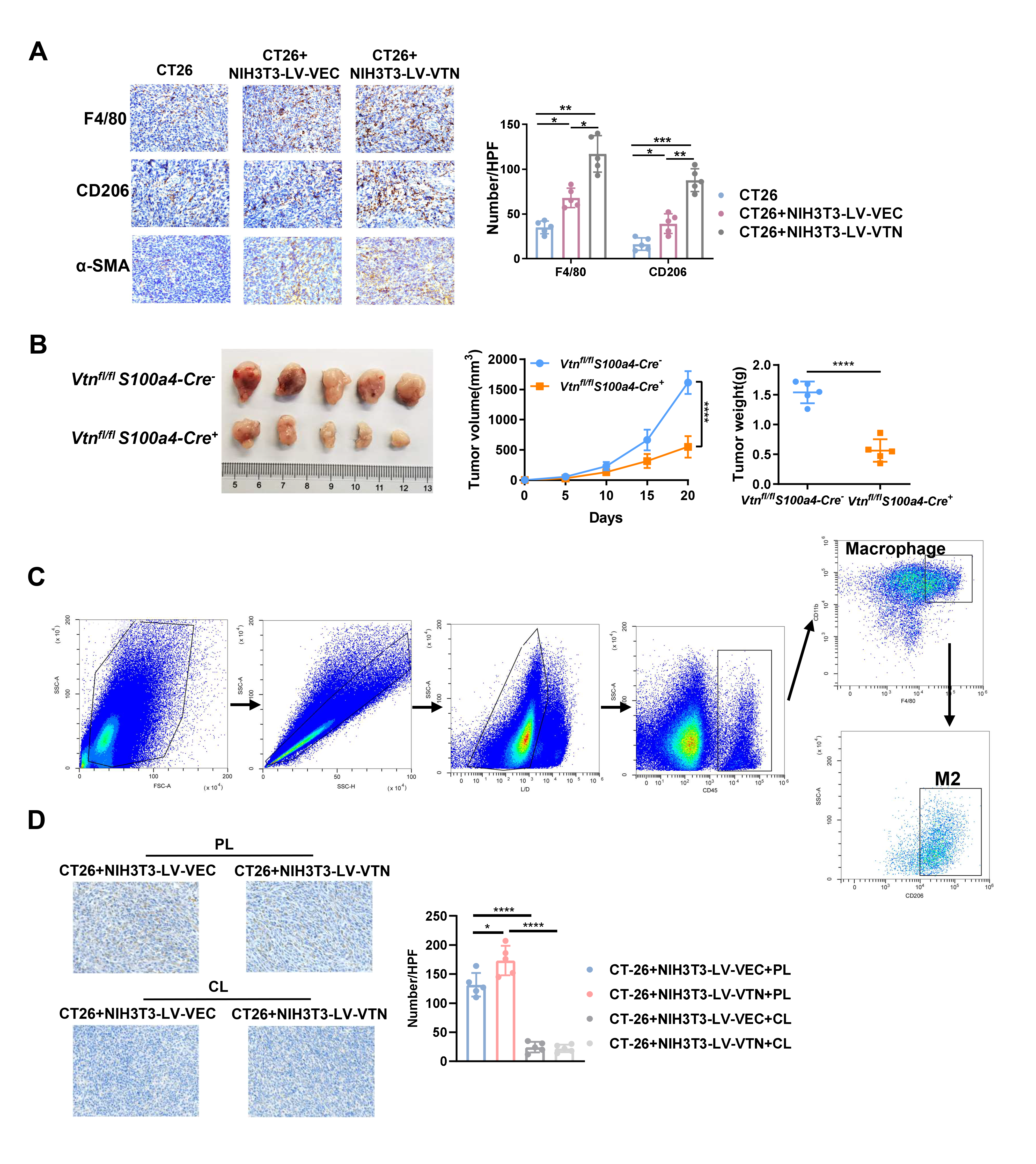

Supplement: Supplementary file 7 — Supporting Information [file ADVS-12-e05769-s010.tif]

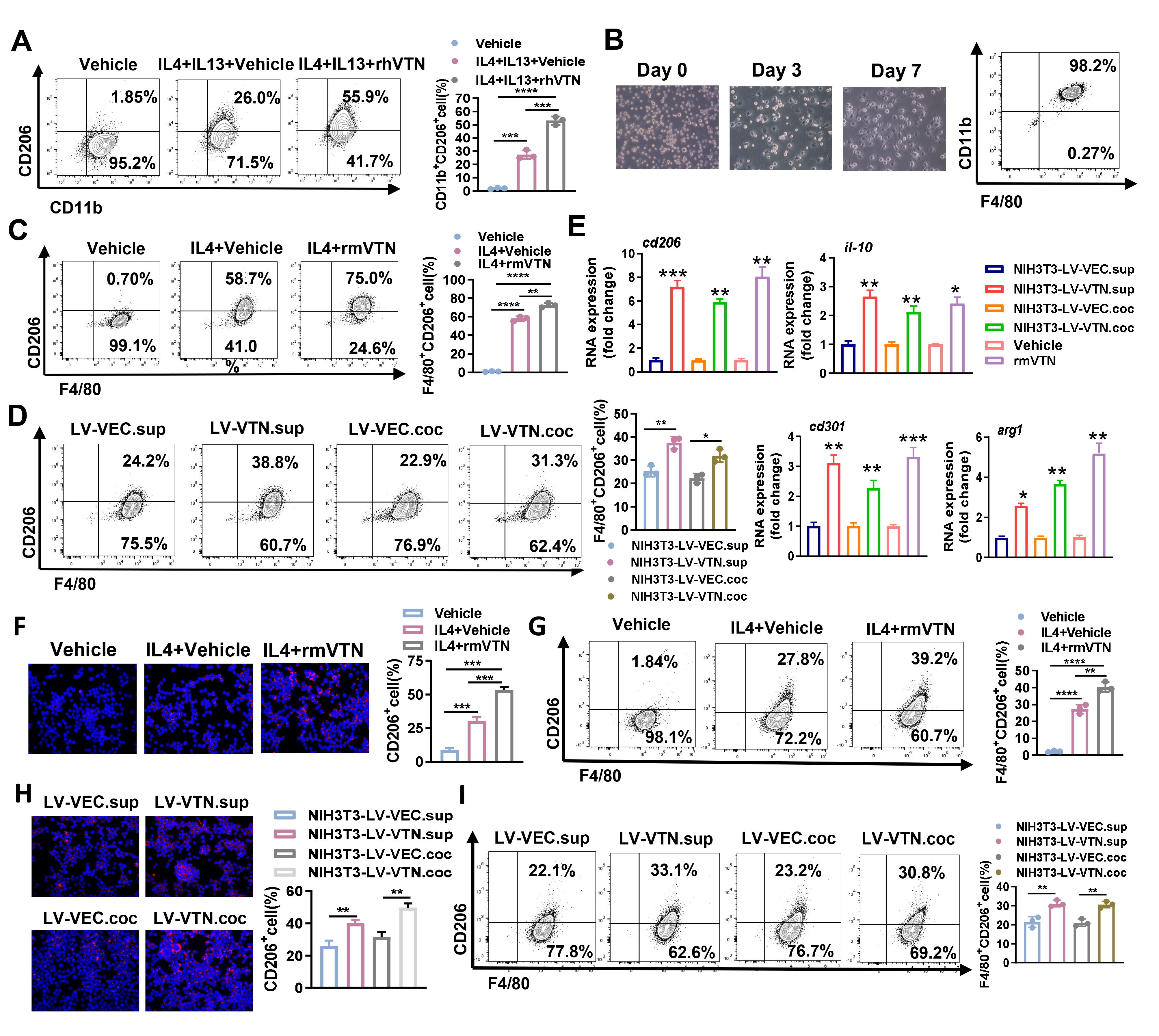

Supplement: Supplementary file 8 — Supporting Information [file ADVS-12-e05769-s004.tif]

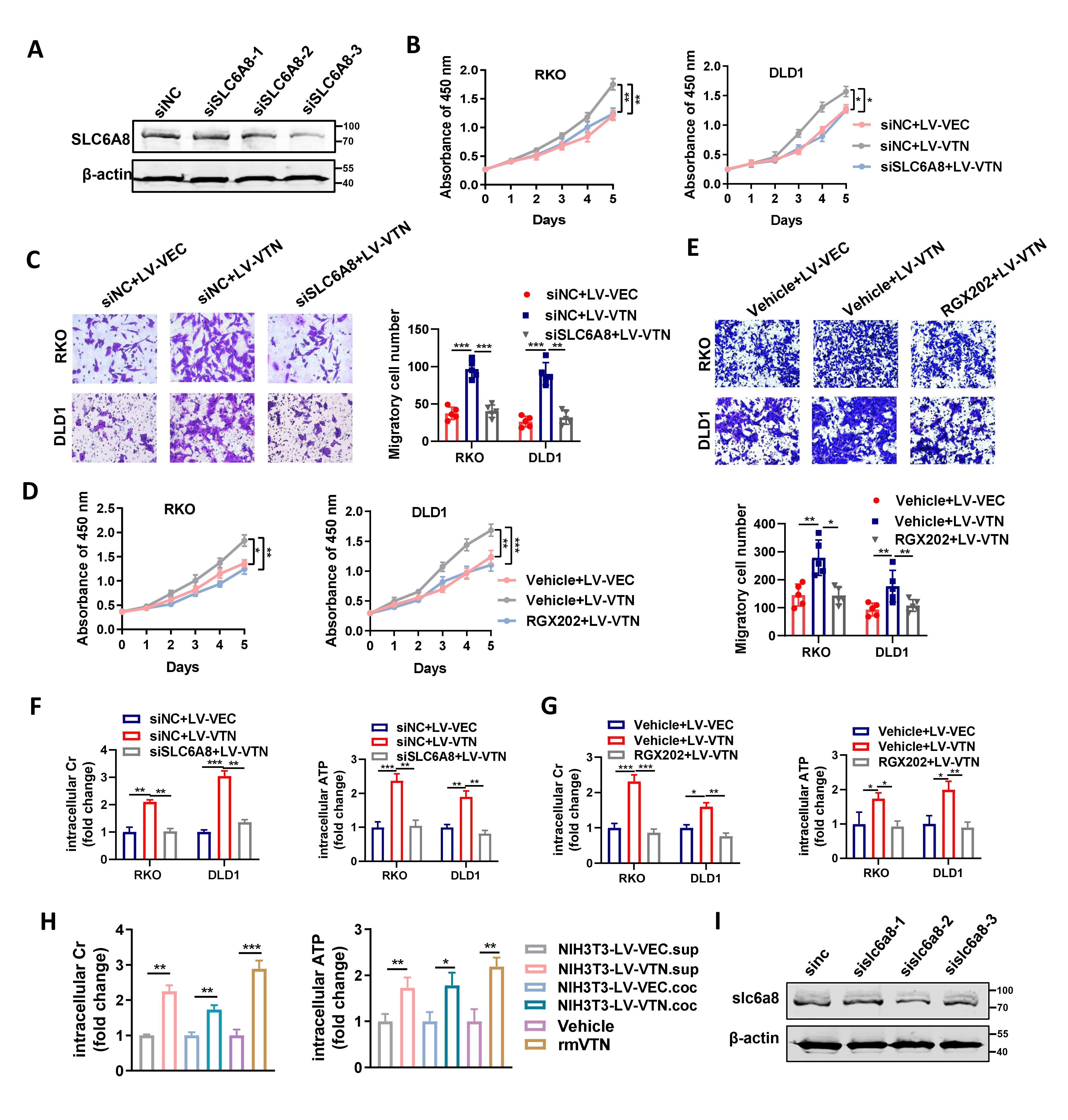

Supplement: Supplementary file 9 — Supporting Information [file ADVS-12-e05769-s008.tif]

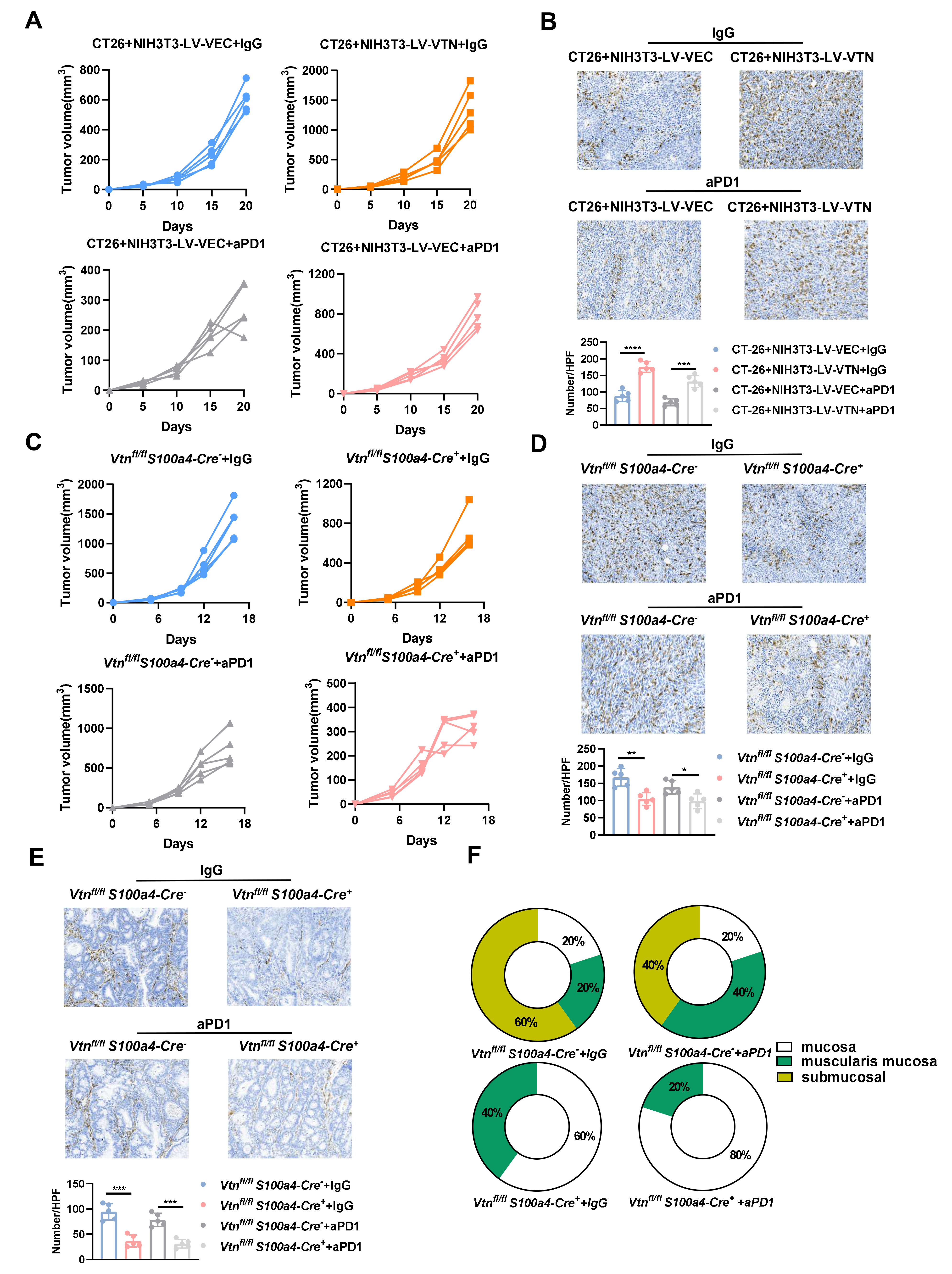

Supplement: Supplementary file 10 — Supporting Information [file ADVS-12-e05769-s007.tif]
